# Label3DMaize: toolkit for 3D point cloud data annotation of maize shoots

**DOI:** 10.1093/gigascience/giab031

**Published:** 2021-05-07

**Authors:** Teng Miao, Weiliang Wen, Yinglun Li, Sheng Wu, Chao Zhu, Xinyu Guo

**Affiliations:** College of Information and Electrical Engineering, Shenyang Agricultural University, Dongling Road, Shenhe District, Liaoning Province, Shenyang 110161, China; Beijing Research Center for Information Technology in Agriculture, 11#Shuguang Huayuan Middle Road, Haidian District, Beijing 100097, China; National Engineering Research Center for Information Technology in Agriculture, 11#Shuguang Huayuan Middle Road, Haidian District, Beijing 100097, China; Beijing Key Lab of Digital Plant, 11#Shuguang Huayuan Middle Road, Haidian District, Beijing 100097, China; National Engineering Research Center for Information Technology in Agriculture, 11#Shuguang Huayuan Middle Road, Haidian District, Beijing 100097, China; Beijing Key Lab of Digital Plant, 11#Shuguang Huayuan Middle Road, Haidian District, Beijing 100097, China; Beijing Research Center for Information Technology in Agriculture, 11#Shuguang Huayuan Middle Road, Haidian District, Beijing 100097, China; National Engineering Research Center for Information Technology in Agriculture, 11#Shuguang Huayuan Middle Road, Haidian District, Beijing 100097, China; Beijing Key Lab of Digital Plant, 11#Shuguang Huayuan Middle Road, Haidian District, Beijing 100097, China; College of Information and Electrical Engineering, Shenyang Agricultural University, Dongling Road, Shenhe District, Liaoning Province, Shenyang 110161, China; Beijing Research Center for Information Technology in Agriculture, 11#Shuguang Huayuan Middle Road, Haidian District, Beijing 100097, China; National Engineering Research Center for Information Technology in Agriculture, 11#Shuguang Huayuan Middle Road, Haidian District, Beijing 100097, China; Beijing Key Lab of Digital Plant, 11#Shuguang Huayuan Middle Road, Haidian District, Beijing 100097, China

**Keywords:** Label3DMaize, 3D point cloud, segmentation, maize shoot, data annotation

## Abstract

**Background:**

The 3D point cloud is the most direct and effective data form for studying plant structure and morphology. In point cloud studies, the point cloud segmentation of individual plants to organs directly determines the accuracy of organ-level phenotype estimation and the reliability of the 3D plant reconstruction. However, highly accurate, automatic, and robust point cloud segmentation approaches for plants are unavailable. Thus, the high-throughput segmentation of many shoots is challenging. Although deep learning can feasibly solve this issue, software tools for 3D point cloud annotation to construct the training dataset are lacking.

**Results:**

We propose a top-to-down point cloud segmentation algorithm using optimal transportation distance for maize shoots. We apply our point cloud annotation toolkit for maize shoots, Label3DMaize, to achieve semi-automatic point cloud segmentation and annotation of maize shoots at different growth stages, through a series of operations, including stem segmentation, coarse segmentation, fine segmentation, and sample-based segmentation. The toolkit takes ∼4–10 minutes to segment a maize shoot and consumes 10–20% of the total time if only coarse segmentation is required. Fine segmentation is more detailed than coarse segmentation, especially at the organ connection regions. The accuracy of coarse segmentation can reach 97.2% that of fine segmentation.

**Conclusion:**

Label3DMaize integrates point cloud segmentation algorithms and manual interactive operations, realizing semi-automatic point cloud segmentation of maize shoots at different growth stages. The toolkit provides a practical data annotation tool for further online segmentation research based on deep learning and is expected to promote automatic point cloud processing of various plants.

## Introduction

Plant structure and morphology are important features for expressing growth and development. At present many research studies underpin the significance of integrating the 3D morphological characteristics of plants when conducting genetic mapping, adaptability evaluation, and crop yield analysis [1, 2]. Using 3D data acquisition technology to obtain a 3D point cloud is the most effective way to perceive plant structure and morphology digitally. However, 3D point clouds are initially obtained in an unordered, unstructured manner and with little semantic information. Therefore, it is critical to use computer graphics technologies and plant morphology knowledge to convert the unstructured 3D point clouds into well-organized and structured data that contain rich morphological features with semantic information. Therefore, plant morphology research based on measured point clouds forms a critical component of 3D plant phenomics [3–6], 3D plant reconstruction [2, 7], and functional-structural plant models [8, 9].

The development of 3D data acquisition technology [10] has significantly enriched approaches for fine-scale 3D data acquisition of individual plants, including 3D scanning [11, 12], LiDAR [13], depth camera [14], time-of-flight reconstruction [15], and multi-view stereo (MVS) reconstruction [16, 17]. Owing to the low cost of sensors and better quality of reconstructed point clouds, MVS reconstruction has been widely adopted in many applications. Recently, multi-view image acquisition platforms that can realize semi-automatic and high-throughput 3D data acquisition for individual plants have been developed [18–21] and enable 3D data acquisition for the phenotypic analysis of large-scale breeding materials [22, 23]. However, how to efficiently and automatically process the acquired big data of 3D point clouds is a bottleneck in 3D plant phenotyping.

The key technologies for 3D point cloud data processing include data registration, extraction of the region of interest, denoising, segmentation, feature extraction, and mesh generation. Among these tasks, point cloud segmentation is challenging. Therefore, automatic and accurate point cloud segmentation could significantly affect subsequent results of phenotype extraction and 3D reconstruction. Point cloud segmentation can be classified as population-shoot or shoot-organ segmentation. Population-shoot segmentation allows for automatic segmentation of maize populations under low density [24] or at early growth stages [25, 26] with little overlap, which can be realized via the spatial distance between shoots. However, it is difficult to achieve automatic segmentation of high-density populations or those with many overlapping organs in late growth stages. Comparatively, more attention has been paid to shoot-organ segmentation. Though high-quality input point clouds and restricted connections between organs are required, color-based [27] and point clustering [28–30] approaches have also been widely used. For instance, Elnashef et al. [16] used the local geometric features of the organs to segment maize leaves and stems at the 6-leaf stage. Paulus et al. [31, 32] segmented grape shoot organs by integrating fast point feature histograms, support vector machine (SVM), and region growing approaches. However, these methods can only segment plant shoots with clear connection characteristics between stems and leaves [11] and have difficulty solving leaf-wrapping stem segmentation problems. For time-series 3D point clouds, the leaf multi-labeling segmentation method was used for organ segmentation and plant growth monitoring [33]. While plant organs could also be segmented through skeleton extraction and hierarchical clustering [34, 35], these methods need interactive manual correction for complex plants to guarantee the segmentation accuracy. Jin et al. [36] proposed a median normalized vector growth algorithm that can segment the stems and leaves of maize shoots. On this basis, an annotation dataset of maize shoots was constructed, and the deep learning method was introduced to improve the automatic segmentation level [37]. However, parameter interactions are still needed for different shoot architecture and cannot meet the needs of high realistic 3D reconstruction.

Owing to the complexity of plant morphology and structure, almost all 3D point cloud segmentation methods for plants need certain manual interactions, which is inconvenient for huge amounts of point cloud data processing, and substantially decreases the efficiency. Therefore, it is necessary to improve the automation of segmentation and increase the throughput of 3D point cloud data processing for plants. Deep learning approaches can effectively solve this problem [21, 38, 39], among which the construction of high-quality training datasets is a prerequisite. For example, LabelMe [40] can realize high-quality data annotation for image segmentation. However, 3D point cloud tools for data annotation are rare, especially for plants. Besides, current datasets used for point cloud segmentation are oriented to general segmentation tasks [41–44]. The existing datasets for 3D plant segmentation contain only small amounts of data [21, 45, 46], which cannot meet the data requirements for high-quality deep learning models.

Because point cloud annotation of plants is labor-intensive and time-consuming, deep learning approaches can be applied to segment plant point clouds. Hence, how to improve the efficiency of high-quality data annotation and develop supporting software tools is the key to automatic point cloud segmentation of plants by deep learning. To meet this data annotation demand, the present study used maize as an example and proposes a top-down point cloud segmentation algorithm. In addition, the toolkit Label3DMaize for point cloud annotation of maize shoots is developed, which could provide technical support for automatic and high-throughput processing of plant point clouds. The toolkit integrates clustering approaches and computer interactions supported through maize structural knowledge. Optimal transportation-based coarse segmentation is satisfactory for basic segmentation tasks, and fine segmentation offers users a way to calibrate the segmentation details. This plant-oriented tool could be used to segment point cloud data of various maize growth periods and provide a practical data-labeling tool for segmentation research based on deep learning.

## Materials and Methods

### Field experiment and data acquisition

Three maize cultivars, including MC670, Xianyu 335 (XY335), and NK815, were planted on 20 May 2019 at the Tongzhou experimental field of Beijing Academy of Agriculture and Forestry Sciences (116 70.863 E, 39 70.610 N). The planting density of all the plots was 6 plants/m^2^ with a row spacing of 60 cm. Morphological representative shoots of each cultivar at sixth leaf (V6), ninth leaf (V9), 13th leaf (V13), and blister (R2) stages [47] were selected and transplanted into pots. Then multi-view images were acquired using the MVS-Pheno platform [18], after which 3D point clouds of the shoots were reconstructed. For validation, 12 shoot point clouds at 4 growth stages (V3, V6, V9, and V12) were acquired using a 3D scanner (FreeScan X3, Tianyuan Inc., Beijing, China), to test the segmentation performance of a different data source.

### Overview of the segmentation pipeline

The point cloud of a maize shoot can be segmented into 5 instances: stem, leaf, tassel, ear, and pot. The stem, tassel, and pot on a shoot can be regarded as an instance for each. For each transplanted shoot at stage R2, assuming that it contains *n*_1_ ears and *n*_2_ leaves, the point cloud of this shoot can thus be segmented into *N* = 3 + *n*_1_+ *n*_2_ instances. ${\emptyset _{\mathrm{u}}}$ represents the point cloud to be segmented, and $\emptyset _{\mathrm{s}}^i\,\,( {i\,\, = \,\,1,\,\,2,\,\, \ldots \ldots ,{\mathrm{\,\,}}N} )$ represent the *i*th point cloud instance. In particular, $\emptyset _{\mathrm{s}}^1$ and $\emptyset _{\mathrm{s}}^N$ refer to the stem and pot (if exists) instance, respectively. Before the segmentation begins, ${\emptyset _{\mathrm{u}}}$ contains all the points of the shoot, and $\emptyset _{\mathrm{s}}^i$ are all empty. With the progression of segmentation, the points in ${\emptyset _{\mathrm{u}}}$ are gradually assigned to $\emptyset _{\mathrm{s}}^i$. The segmentation completes when ${\emptyset _{\mathrm{u}}}$ is empty.

**Figure 1:**
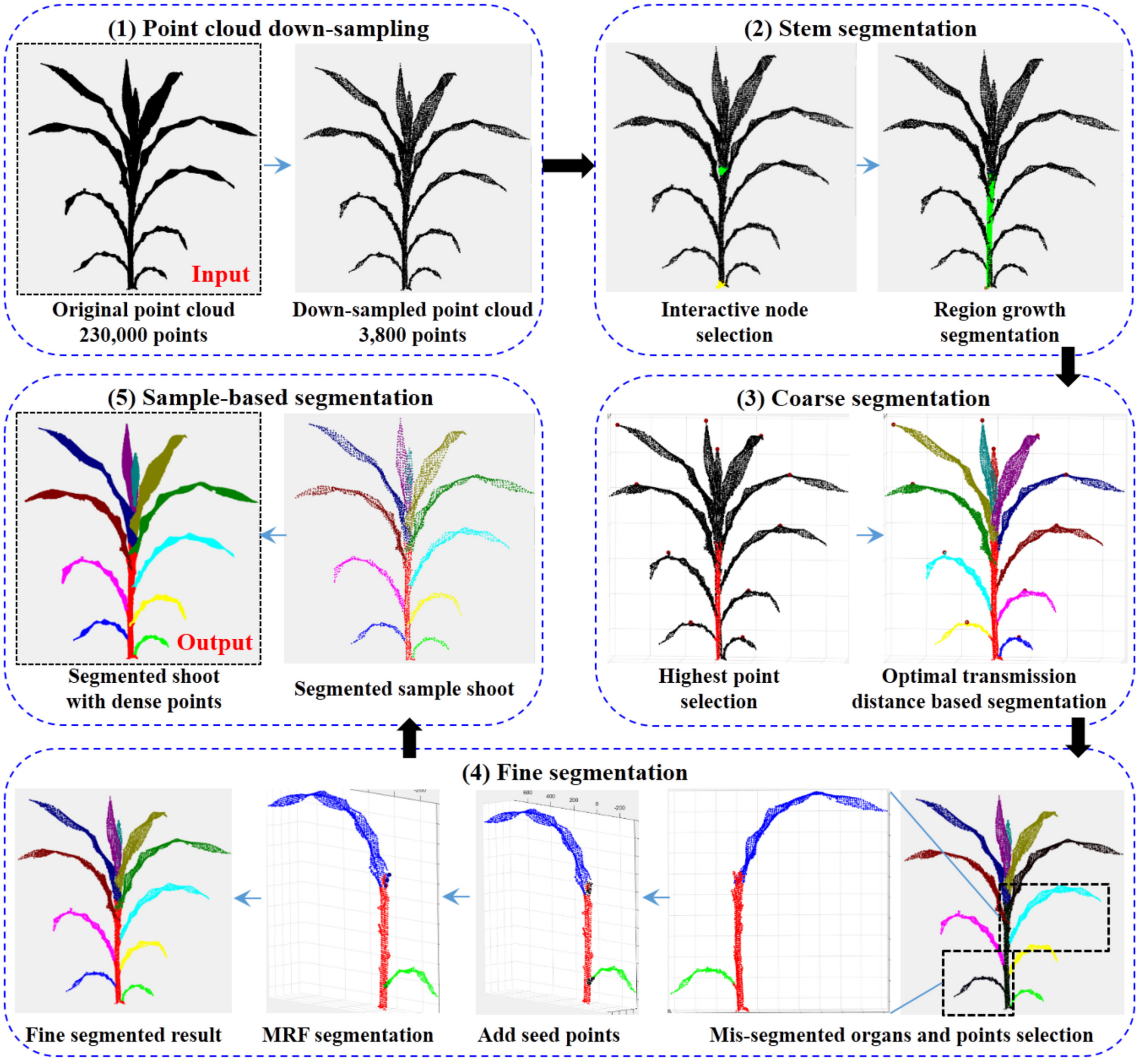
Workflow of the segmentation.

The segmentation pipeline includes 5 parts (Fig. 1): point cloud down-sampling, stem segmentation, coarse segmentation, fine segmentation, and sample-based segmentation. (1) Point cloud down-sampling: the original input point cloud is down-sampled to maintain the shoot morphological features, which improves the segmentation efficiency and quickens the entire segmentation process. (2) Stem segmentation: the top and bottom points of the stem are interactively selected, and the corresponding radius parameters are interactively adjusted. Subsequently, the median region growth is applied to segment the stem points from the shoot automatically. (3) Coarse segmentation: the highest points of each organ instance, except the stem, are obtained via manual interaction, after which all organ instances are segmented automatically on the basis of the optimal transportation distances. (4) Fine segmentation: the unsatisfactory segmentation point regions are selected interactively, and the seed points of organ instances are selected. Organs are then segmented by Markov random fields (MRF). (5) Sample-based segmentation: maize shoots with high-resolution point clouds are segmented on the basis of the fine segmentation result of low-resolution point clouds.

### Stem segmentation

Two seed points ${s_0}$ and ${s_n}$ at the bottom and top of each stem were selected interactively. Then, a median-based region growing algorithm [36] was applied to segment the stem points. This segmentation procedure updates the seed point iteratively along the direction from ${s_0}$ to ${s_n}$. Points around the seed points were classified into stem points. Supposing the algorithm is currently at the *k*th iteration and the seed point is ${s_k}$, the segmentation process was evaluated as follows:

Step1: Points lying in a sphere were classified as stem points, where ${s_k}$ is the center of the sphere, ${r_1}$ is its radius, and ${r_1}$ is a user-specified parameter.

Step 2: The growth direction $\vec v_k$ was determined according to:

\begin{equation*} \,\,\vec v_k = \,\,\left( {\alpha \vec v_1 + {\mathrm{\beta }}\vec v} \right)/{\left\| {\alpha \vec v_1 + {\mathrm{\beta }}\vec v} \right\|_2}, \end{equation*}

 

\begin{equation*} \,\,\vec v_1 = \,\,\mathrm{median}\left\{ {\left( {{p_A} - {s_k}} \right)/{{\left\| {{p_A} - {s_k}} \right\|}_2},{p_A} \in A} \right\}, \end{equation*}

 

\begin{equation*} \,\,\vec v = \left( {{s_n} - {s_k}} \right)/{\left\| {{s_n} - {s_k}} \right\|_2}. \end{equation*}

In this formula, ${\| {} \|_2}$ is L_2_ normal form and median {} represents the median operation. $\alpha $ and ${\mathrm{\beta }}$ are weight parameters set by users and $\vec v_1$ is the normalized vector from the median of already segmented points of the stem to the seed point ${s_k}$. Meanwhile, $\vec v$ is the normalized vector from ${s_k}$ to ${s_n}$, which corrects the growth direction to coincide with the stem. In practice, $\alpha \,\, = {\mathrm{\,\,}}0.2$ while ${\mathrm{\beta \,\,}} = {\mathrm{\,\,}}0.8$. This parameter setting ensures that the stem points can be correctly segmented under different ${r_1}$ values during the entire growing process.

Step 3: A new seed point ${s_{k + 1}}$ for the next iteration was estimated according to ${s_{k + 1}} = {s_k}\,\, + {r_1}\vec v_k$.

Step 4: Region growth finish condition judgement. Supposing *L* represents the line segment from ${s_0}$ to ${s_n}$, then project ${s_{k + 1}}$ on *L*. If the projection point was not on *L*, it indicates that the current regional growth was beyond the stem region and the iteration should be stopped. Otherwise, continue the *k*+ 1 times iteration and execute Step 1.

**Figure 2:**
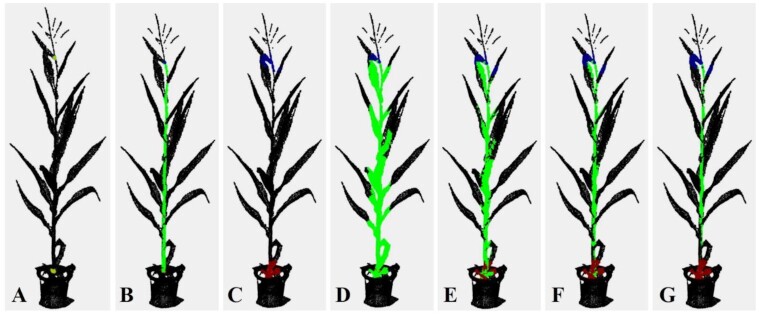
Stem point cloud segmentation. (A) Seed points at the bottom and top of the stem are interactively selected, and an appropriate segmentation radius is set. (B) Stem segmentation result based on (A). (C) A big radius is set. (D) Segmentation result based on (C). (E–G) Stem segmentation results with 1, 2, and 3 median operations based on (D).

Because the maize stem gradually thins from bottom to top, a uniform radius ${r_1}$ may generate over-segmentation, i.e., classifying the points of other organs into the stem. Besides, the region growing algorithm also over-segments points in some regions where the stem bends. Therefore, a simple median operation was adopted to eliminate the over-segmented points. First, the already segmented stem points were evenly divided into *M* segments along the direction of $( {{s_n} - {s_0}} )/{\| {{s_n} - {s_0}} \|_2}$, and the median axis of each segment was fitted using least squares. The average distance from each point to the central axis was then calculated. If the distance from a point to the central axis was less than the average distance, it was retained as the stem point; otherwise it was removed from the stem to the unsegmented point set. Users can perform the median operation several times in the toolkit to reduce the over-segmentation problem. Although multiple median operations cause an under-segmentation of the stem point cloud, the issue is resolved in the subsequent organ segmentation processes. $\emptyset _{\mathrm{s}}^1$ represents the segmented stem points, and these points are removed from ${\emptyset _{\mathrm{u}}}$. Subsequent organ segmentation is performed in the remaining point cloud. Stem point cloud segmentation is illustrated in Fig. 2.

### Shoot alignment

The shoot points were transformed into a regular coordinate system to access the position of each point in the cloud conveniently. The midpoint of the already segmented stem point cloud was taken as the origin *O* of the new shoot coordinate system. In contrast, the Z-axis of the new coordinate system was the middle axis estimated by the least-squares method from the stem point cloud. Then, the shoot point cloud was projected onto the plane using the Z-axis as its normal vector. The first and second principal component vectors of the projection points were determined by principal component analysis and assigned as the X- and Y-axis of the new shoot coordinate system, respectively. Subsequently, the original point cloud coordinates were transformed into the new shoot coordinate system, and the coordinates of their *z*-value judged the height of points in the shoot. Points are higher with greater *z*-values.

### Coarse segmentation of organs

**Figure 3:**
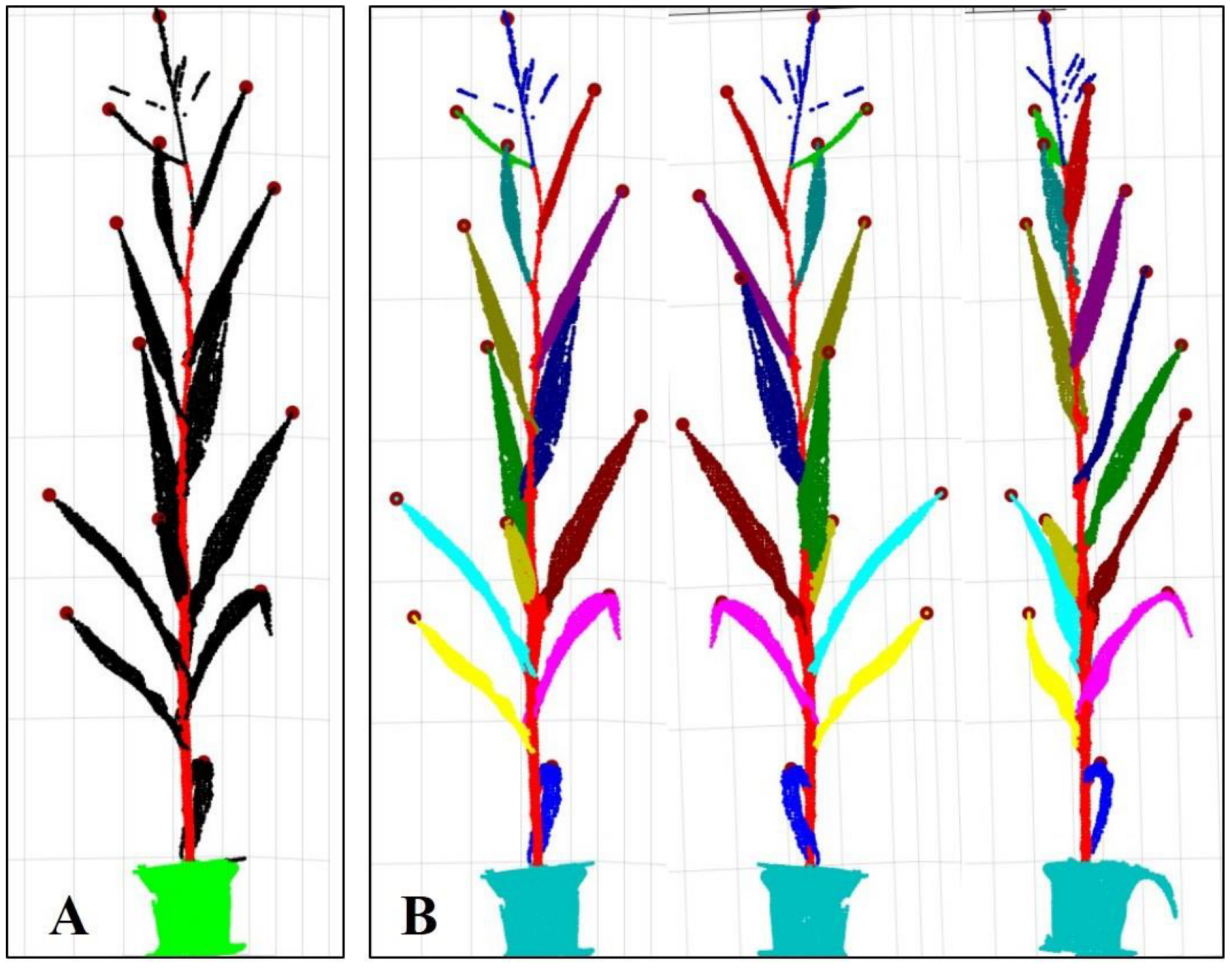
Illustration of coarse segmentation. (A) Highest point determination of each organ. (B) Visualization of segmented shoot from different angles of view.

A top-down point cloud segmentation algorithm for maize organs from a shoot was applied. The highest point of each organ was taken as the seed point of the organ (Fig. 3A). The other shoot points after stem segmentation were classified into corresponding organ instances from the top down by the optimal transportation distances (Fig. 3B).

#### Organ seed points determination

After stem segmentation, the point cloud of maize shoots was spatially divided into several relatively discrete point clouds (excluding the stem). However, the exact number of organs is always larger than the number of discrete point clouds, owing to the spatial organ connection, especially near the upper leaves. Thus, the seed point for each organ has to be determined for the next step segmentation. The highest point of each organ was regarded as the seed point (Fig. 3A). If a pot was involved in the point cloud, all points with a *z*-value less than the lowest point of the stem were directly classified as pot points. Usually, the highest point of a new leaf appears at the tip region; the middle and lower fully unfolded leaves are mostly curved. Meanwhile, the highest point lies in the middle of the leaf, and the highest points of a tassel or ear are at the top. Therefore, it was assumed that the distance between the highest points of any 2 organs was >5 cm. On this basis, the highest point of each organ was determined by searching for the point with the maximum *z*-value within the point cloud of the organ.

Owing to the complicated spatial points at the organ connection areas, automatic estimation of the highest points of instances may not be accurate. Label3DMaize provides a manual interaction module to determine the highest seed point of each organ. Simultaneously, this operation can also assign a serial number to each organ for further output. Because the number of maize organs is relatively small, this interactive correction operation is convenient and acceptable. The derived seed points of each organ are set into the corresponding instance point cloud $\emptyset _{\mathrm{s}}^i$. At this time, each leaf, tassel, and ear instance point cloud only contains the highest point, and there are multiple points in the pot and stem instances.

#### Coarse segmentation based on optimal transportation distances

After obtaining the seed points of all the instances, the left points in ${\emptyset _{\mathrm{u}}}$ were traversed 1 by 1 to determine the instance to which they belong. For each point to ${\emptyset _{\mathrm{u}}}$, the distance between the point and each other point cloud instance was evaluated, and it was classified into the nearest instance. The classified points were evaluated from top to bottom; i.e., the points with bigger *z*-coordinates were evaluated preferentially. The process was as follows:

Step 1: The points in the point set ${\emptyset _{\mathrm{u}}}$ were reordered from big to small according to their *z*-values.

Step 2: For point $p \in {\emptyset _{\mathrm{u}}}$, the organ instance it belongs to was determined. The distance ${d^i}$ from point *p* to the *i*th instance was defined as

\begin{equation*} \,\,{d^i} = {D_s}\,\,\left( {p,\widetilde {{p^i}}} \right), \end{equation*}

where ${D_s}$ is the optimal transportation distance between any 2 points calculated based on the Sinkhorn algorithm [48]. Then point *p* is assigned into the organ instance with the lowest ${d^i}$. $\widetilde {{p^i}}$, in the *i*th instance, is the nearest neighbor of point *p* under the optimal transportation distance.

Step 3: Move point *p* from ${\emptyset _{\mathrm{u}}}$ into the corresponding $\emptyset _{\mathrm{s}}^i$. Continue traversing the next point in ${\emptyset _{\mathrm{u}}}$, and perform Step 2 until ${\emptyset _{\mathrm{u}}}$ is empty.

The detailed description of ${D_s}$ in Step 2 is as follows. The optimal transportation strategy of point cloud *Q* to its identical set $Q^{\prime}$ is the one that transmits all the quality of any point $p \in Q$ to the same point $p^{\prime} \in Q^{\prime}$. The Sinkhorn algorithm [48] was used here to calculate the optimal transportation distances. It allocates the quality of any point $p \in Q$ to all points in $Q^{\prime}$. A point with higher allocation quality suggests the point is closer to *p* than any other points under the optimal transportation strategy. Suppose that point cloud *Q* contains ${N_Q}$ points. $Q^{\prime}$ represents the same point set of *Q*. ${p_u}$ is the *u*th point in *Q*, and ${M_u}$ indicates the quality of point ${p_u}$. Similarly, $p_v^{\prime}$ is the *v*th point in $Q^{\prime}$, and $M_v^{\prime}$ indicates the quality of point $p_v^{\prime}$. ${m_{uv}}$ represents the transported quality from ${p_u} \in Q$ to $p_v^{\prime} \in Q^{\prime}$. Then the optimal transportation energy from point cloud *Q* to point cloud $Q^{\prime}$ can be described as:

\begin{equation*} \mathop {{\mathrm{argmin}}}\limits_m \mathop \sum \nolimits_{u\,\, = \,\,1}^{{N_Q}} \mathop \sum \nolimits_{v\,\, = \,\,1}^{{N_Q}} {m_{uv}}\left\| {{p_u} - p_v^{\prime}} \right\| + \frac{1}{\varepsilon }\mathop \sum \nolimits_{u\,\, = \,\,1}^{{N_Q}} \mathop \sum \nolimits_{v\,\, = \,\,1}^{{N_Q}} {m_{uv}}\log {m_{uv}}, \end{equation*}

 

\begin{equation*} {\mathrm{s}}.{\mathrm{t}}.{\mathrm{\,\,}}{m_{uv}} > 0;{\mathrm{\,\,}}\mathop \sum \nolimits_{v\,\, = \,\,1}^{{N_Q}} \,\,{m_{uv}} = {M_u}\,\,;\,\,\mathop \sum \nolimits_{u\,\, = \,\,1}^{{N_Q}} \,\,{m_{uv}} = M_v^{\prime}\,\,. \end{equation*}

In this equation, $\varepsilon $ is the adjustment parameter, which was set to 5 in this article, and $\| {} \|$ is the L_2_ normal form. The above equation can be solved by the Sinkhorn matrix scaling algorithm [49], and the optimal transportation from *Q* to $Q^{\prime}$ can be derived; i.e., an ${N_Q} \times {N_Q}$ optimal transportation matrix *M* is obtained. The element ${m_{uv}}$ at *u* row and *v* column in the matrix is the transported quality from the *u*th to the *v*th point. A larger ${m_{uv}}$ indicates that the 2 points are closer. After obtaining the optimal transportation solution, the optimal transportation distance from the *u*th to the *v*th point in the point cloud can be defined as ${D_s}\,\,( {{p_u},\,\,{p_v}} ) = 1/m_{uv}$. The pseudocode for calculating the optimal transportation distance *M* is shown in Table 1.

**Table 1:** Pseudocode for calculating optimal transportation matrix

**Figure 4:**
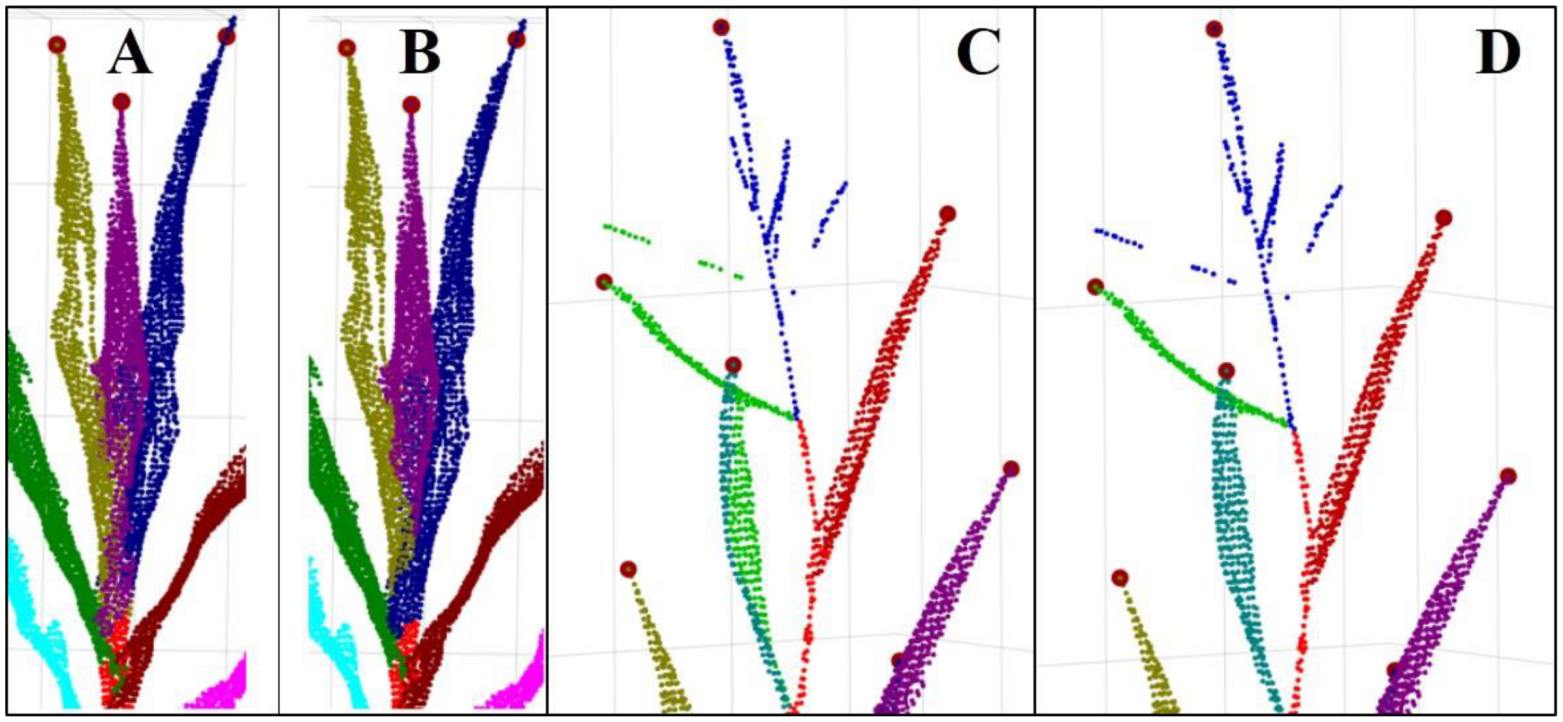
Organ segmentation comparison using optimal transportation distance and Euclidean distance. Point cloud segmentation result for big leaf wrapping small leaf base case using Euclidean distance (A) and optimal transportation distance (B). Point cloud segmentation result for close or slight organ adhesion case using Euclidean distance (C) and optimal transportation distance (D).

Algorithm 1. Computation of optimal transportation matrix *M*, using MATLAB syntax.
**Input:** Parameter *ε*; Point cloud matrix *Q_NQ_*_×3_; % *NQ* is the point number of the point cloud
*n* = *N*_Q_;
*H_n_*_×_ *_n_* **= pdist2(***Q, Q***);***H* **=** *H***./max**(*H*(:));
*K_n_*_×_ *_n_* = **exp**(-ε*H*);
*U_n_*_×_ *_n_* = *K*.*H;
*a_n_*_×1_ = **ones**(*n*,1)/*n*;
h*_n_*_×1_ = *a*;
*J_n_*_×_ *_n_* = **diag**(1./*a*)*K;
**while** *h* **changes or any other relevant stopping criterion do**
*h* = 1./(J*(*a*./(*h*'*K)'));
**end while**
*z _n_*_×1_ = *a*./((*h*'*K)');
*M _n_*_×_ *_n_* **= diag**(*h*(:,1)) * *K* * **diag**(*z*(:,1));

In the optimal transportation energy equation, when parameter $\varepsilon $ increases, the transportation strategy gets closer to the classical optimal transportation, and the segmentation result using optimal transportation distance ${D_s}$ is also closer to that using Euclidean distance. The same results can be derived using the 2 distances when the *ε* is >100. When *ε* is smaller, the solution becomes smoother, and the nearest neighbor calculated under the ${D_s}$ distance tends to the region with higher point density. Compared with the Euclidean distance, using the optimal transportation distance to estimate the distance between points can better deal with the challenge of big leaves wrapping on leaflets than using the Euclidean distance (Fig. 4A and B). When the adhesion area of the 2 organs is not significantly large, the segmentation result using the optimal transportation distance is better than that of the Euclidean distance (Fig. 4C and D).

### Fine segmentation of organs

Coarse segmentation can provide preliminary results, but false segmentation is frequently observed in the intersecting regions of organs. To obtain more precise segmentation results, this study developed a fine segmentation module for organs in Label3DMaize, which included the following processes:

Step 1: *n* (*n* > 1) organ instances to be fine segmented were selected, and $\emptyset _{{\mathrm{s^{\prime}}}}^i$ represents the *i*th instance.

Step 2: The region of interest was selected among the above instance point cloud, represented by $\emptyset _{{\mathrm{u^{\prime}}}}^i$.

Step 3: The seed point for the *i*th instance $\emptyset _{{\mathrm{s^{\prime}}}}^i$ was selected from region ${\emptyset _{{\mathrm{u^{\prime}}}}}$. The selected points were removed from ${\emptyset _{{\mathrm{u^{\prime}}}}}$ and stored in $\emptyset _{{\mathrm{s^{\prime}}}}^i$.

Step 4: The points in ${\emptyset _{{\mathrm{u^{\prime}}}}}$ were re-segmented using MRF.

The re-segment algorithm was detailed using MRF in Step 4, as explained in the following. The fine segmentation of the interest region mentioned above is a multi-classification problem. It allocates ${p_u} \in {\emptyset _{{\mathrm{u^{\prime}}}}}$ into *n* organ instances $\emptyset _{{\mathrm{s^{\prime}}}}^i$, i.e., search for the right organ tag for point ${p_u}$. Hence a mapping function ${f_n}( {{p_u}} )$ is defined for any point ${p_u}$. When a point ${p_u}$ is mapped to the *i*th instance, ${f_n}\,\,( {{p_u}} ) = \,\,i$, the energy function is defined as:

\begin{equation*} E\,\,\left( {{f_{\mathrm{n}}}} \right) = \,\,\gamma \mathop \sum \nolimits_{{p_u} \in {\emptyset _{{\mathrm{u^{\prime}}}}}} {D_{{p_u}}}\left( {{f_{\mathrm{n}}}\left( {{p_u}} \right)} \right) + \mathop \sum \nolimits_{\left( {{p_u},{q_u}} \right) \in \aleph \left( {{p_u}} \right)} V\left( {{f_{\mathrm{n}}}\left( {{p_u}} \right),{f_{\mathrm{n}}}\left( {{q_u}} \right)} \right), \end{equation*}

 

\begin{equation*} {D_{{p_u}}}\,\,\left( i \right) = \,\,D\left( {{p_u},\emptyset _{{\mathrm{s^{\prime}}}}^i} \right){\,\,i\,\,} = {\mathrm{\,\,}}\left[ {1,2 \ldots \ldots {n}} \right], \end{equation*}

 

\begin{equation*} V\,\,\left( {{f_{\mathrm{n}}}\left( {{p_u}} \right),{\mathrm{n}}\left( {{q_u}} \right)} \right) = {\left( {\frac{{d\left( {{p_u},{q_u}} \right)}}{{d^{\prime}}}} \right)^\tau }\,\,{\left( {\frac{{a\left( {{n_p},{n_u}} \right)}}{\pi }} \right)^\varphi }. \end{equation*}

In this function, $\aleph ( {{p_u}} )$ is the *k*-neighborhood of ${p_u} \in {\emptyset _{{\mathrm{u^{\prime}}}}}$. The data item ${D_{{p_u}}}( {{f_{\mathrm{n}}}( {{p_u}} )} )$ measures the loss of classifying ${p_u}$ to *n* instances $\emptyset _{{\mathrm{s^{\prime}}}}^i$. $D( {{p_u},{\mathrm{\,\,}}\emptyset _{{\mathrm{s^{\prime}}}}^i} )$ represents the distance from point ${p_u}$ to instance $\emptyset _{{\mathrm{s^{\prime}}}}^i$, which is the distance from ${p_u}$ to the nearest point in $\emptyset _{{\mathrm{s^{\prime}}}}^i$. $\gamma $ is a weight parameter that controls the proportion of distance term in the energy function. The smooth item $V( {{f_{\mathrm{n}}}( {{p_u}} ),\,\,{f_{\mathrm{n}}}( {{q_u}} )} )$ quantifies the corresponding loss when assigning the tag ${f_{\mathrm{n}}}( {{p_u}} )$ and ${f_{\mathrm{n}}}( {{q_u}} )$ for point ${p_u}$ and ${q_u}$, respectively. This smooth term encourages spatial consistency; i.e., the probability that adjacent points belong to the same class is higher. The smooth term is composed of the product of the distance term on the left and the angle term on the right. Meanwhile, $d( {{p_u},\,\,{q_u}} )$ is the Euclidean distance of the 2 points and $d^{\prime}$ is the maximum Euclidean distance between all points and their neighborhood points, regulating the distance term in the range of (0, 1]. ${n_p}$ and ${n_u}$ are the normal vectors of points ${p_u}$ and ${q_u}$, respectively. $a( {{n_p},\,\,{n_u}} )$ is the angle between the 2 normals. $\tau $ and $\varphi $ are the weight parameters for the distance and angle term, respectively, both with a default value of 1.0. The minimum solution of the energy function is solved by ${\mathrm{\alpha }}$-expansion MRF [50].

In addition, users cloud assign an organ label to the region of interest points after the aforementioned Step 2, which offers a more direct way for fine segmentation.

### Sample-based segmentation

It is suggested that the number of points per shoot should be <15,000 to ensure data processing efficiency. Therefore, Label3DMaize provides point cloud simplification and sample-based segmentation modules. Voxel-based simplification is adopted in the toolkit. Sample-based segmentation refers to the automatic segmentation of a dense point cloud via the segmentation result of the corresponding simplified point cloud. Specifically, suppose that point cloud *A* is the simplification of dense point cloud *B*, and *A* has already been segmented while *B* is to be segmented. The *k*-nearest neighbors in *A* of any point $p \in B$ are calculated, followed by counting how many points of these *k*-nearest neighbors belong to each instance. The instance with the maximum neighbor points is determined as the instance of point *p*.

## Results

### Interface and operations of Label3DMaize

**Figure 5:**
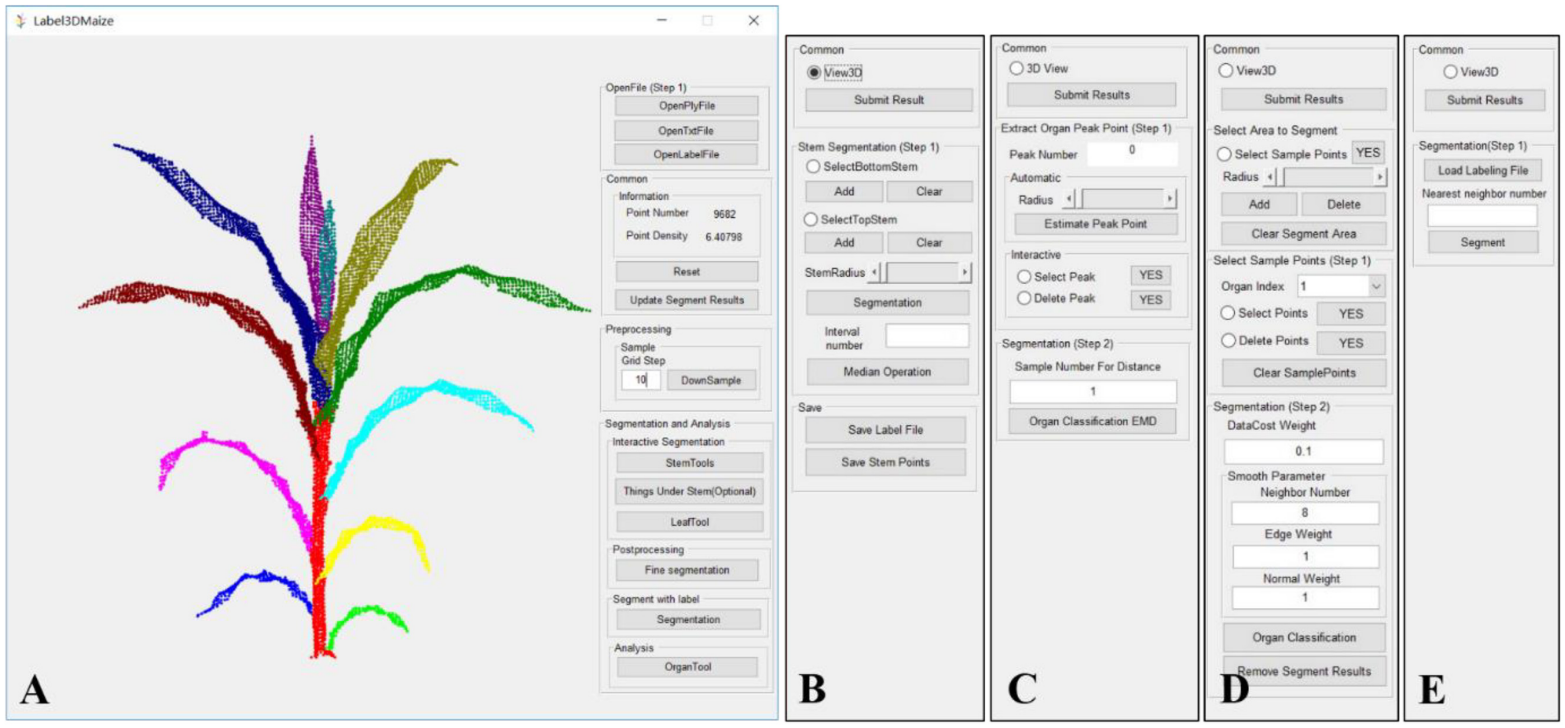
Interfaces of Label3DMaize. (A) The main interface of the toolkit, composed of a visualization window and an embedded dialog. (B–E) Dialog of stem segmentation, coarse segmentation, fine segmentation, and sample-based segmentation. The visualization window is not shown in these sub-interfaces.

The Label3DMaize toolkit was developed using MATLAB. The interface is composed of the main interface and multiple sub-interfaces, including stem segmentation, coarse segmentation, fine segmentation, and sample-based segmentation (Fig. 5). Each sub-interface pops up after the corresponding button on the main interface is triggered. The main interface and each sub-interface are composed of an embedded dialog and an interactive visual window (only the embedded dialog in each sub-interface is shown in Fig. 5). The interactive visual window enables the user to rotate, zoom, translate, select points of interest in the view, and improve the segmentation effect visually and interactively. The input of the toolkit includes point cloud files in text format, such as txt or ply. According to the operational process shown in Fig. 5, segmentation results can be refined step by step by inputting parameters and manually selecting points. The output of the toolkit is a text file with annotation information; i.e., each 3D coordinate point in the text has a classification identification number, and the points with the same identification number belong to the same instance. These format files are applicable for 3D deep learning of maize shoots. The executable program of Label3DMaize can be found in the attachment.

### Visualization and accuracy evaluation

**Figure 6:**
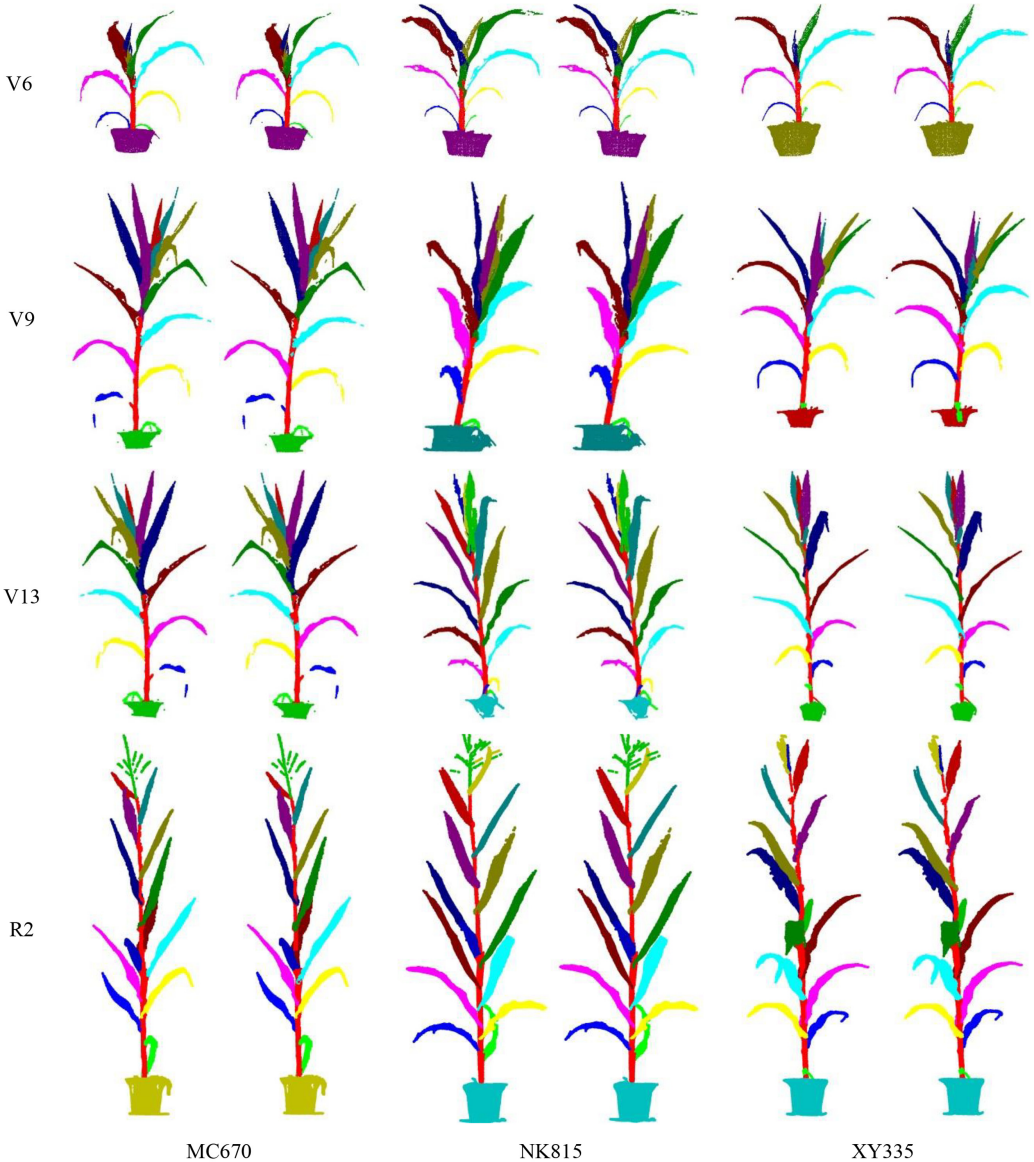
Visualization of maize shoot segmentation results of 3 cultivars at 4 growth stages. In each pair of images, the left and right are coarse and corresponding fine segmentation results, respectively.

To evaluate the accuracy of coarse and fine segmentation, the point clouds of 3 varieties in 4 different growth stages of maize shoots are segmented using Label3DMaize. Figure 6 shows the visualization results. According to the visualization results, no significant differences were observed between the coarse and fine segmentation. Yet, fine segmentation improved the segmentation effect of the details, especially near the connection region of organs.

The present study has further provided numerical accuracy results to quantitatively evaluate the difference between coarse and fine segmentation (Table 2). The precision, recall, and F1-score of each organ were estimated on the basis of fine segmentation as the ground truth. The averaged precision and recall of all shoot organs were taken as the precision and recall. Macro-F1 and micro-F1 are calculated using the precision and recall of the shoot and organs averaged value, respectively. It can be seen from Table 2 that although the accuracy of coarse and fine segmentation differed, the overall difference was not significant.

**Table 2:** Accuracy evaluation of coarse segmentation vs fine segmentation

	Overall accuracy (%)	Precision (%)	Recall (%)	Micro-F1 (%)	Macro-F1 (%)
Mean (range)	97.2 (89.8–99.4)	96.7 (92.0–99.2)	95.6 (84.1–99.1)	96.1 (87.9–99.2)	95.6 (85.3–99.1)

### Segmentation efficiency

The efficiency of plant point cloud segmentation is an essential indicator for the practicality of training data annotation tools for deep learning. Table 3 shows the time consumed in the different steps for maize shoot segmentation at 4 growth stages using Label3DMaize on a workstation (Intel Core i7 processor, 3.2 GHz CPU, 32 GB of memory, Windows 10 operating system), including the interactive manual operations and segmentation computations. It can be seen that point cloud segmentation takes ∼4–10 minutes per shoot, in which coarse segmentation takes ∼10–20% of the total time. In the whole segmentation process, the manual interaction time cost is significantly higher than that of automated computation. The segmentation efficiency is positively related to the number of leaves.

**Table 3:** Segmentation time of different steps on maize shoots at 4 growth stages using Label3DMaize

Growth period	No. points of a maize shoot		Time cost (s)									
	Input	After simplification	t_1_	t_2_	t_3_	t_4_	t_5_	t_6_	t_7_	t_8_	t_9_	Total
V6	45,833	13,196	10	0.2	16	4	30.2	60	0.05	0.5	100	**190.75**
V9	62,523	13,953	10	0.2	21	4	35.2	140	0.05	0.6	100	**275.85**
V13	70,873	12,102	14	0.2	32	5	51.2	260	0.05	0.6	100	**411.85**
R2	71,909	13,224	14	0.2	35	5	54.2	268	0.05	0.6	100	**422.85**

t_1_: Time for stem point selection and radius setting. t_2_: Time for segmentation computation of stem points. t_3_: Time for seed points selection of organ instances. t_4_: Time for organ segment computation. t_5_: Time for coarse segmentation, where t_5_ = t_1_ + t_2_ + t_3_ + t_4_. t_6_: Time for fine segmentation operations. t_7_: Time for fine segmentation computation. t_8_: Time for sample-based segmentation. t_9_: Time for other operations, e.g., the alternation between main and sub-interfaces. Underscored and non-underscored identifiers indicate the time cost for manual interactions and automated computation, respectively.

This study also analyzed the detailed time costs. (i) The time cost of stem segmentation. In the early growth stages of a maize shoot, the stem is relatively upright, so users only need to select the bottom and upper points of the stem and specify a suitable radius. However, in the late growth stages, the maize shoot height increases and the stem becomes thinner from bottom to top. Meanwhile, the upper part is curved, so interactive median segmentation is needed, which increases the segmentation time. (ii) The time cost of coarse segmentation. The major interactive operation of coarse segmentation is that the user selects or adjusts the highest organ points. As the maize shoot grows, the number of organs gradually increases, so the time costs for the interactive operation of picking points also increase. Meanwhile, the growth of shoot organs significantly increases the occlusion among organs. Thus, the appropriate angles of view for users have to be found to determine the highest organ points, which is time-consuming. (iii) The time cost of fine segmentation. An increase in the number of organs causes false segmentation of more organs at the connection regions. Therefore, the fine segmentation of maize shoots with more organs would take more time. Besides, the segmentation efficiency is related to the shoot architecture; the spatial distances between adjacent organs are much larger in flattened shoots than those of relatively compact ones, which increases the segmentation efficiency of flattened shoots.

### Comparison with other methods

**Figure 7:**
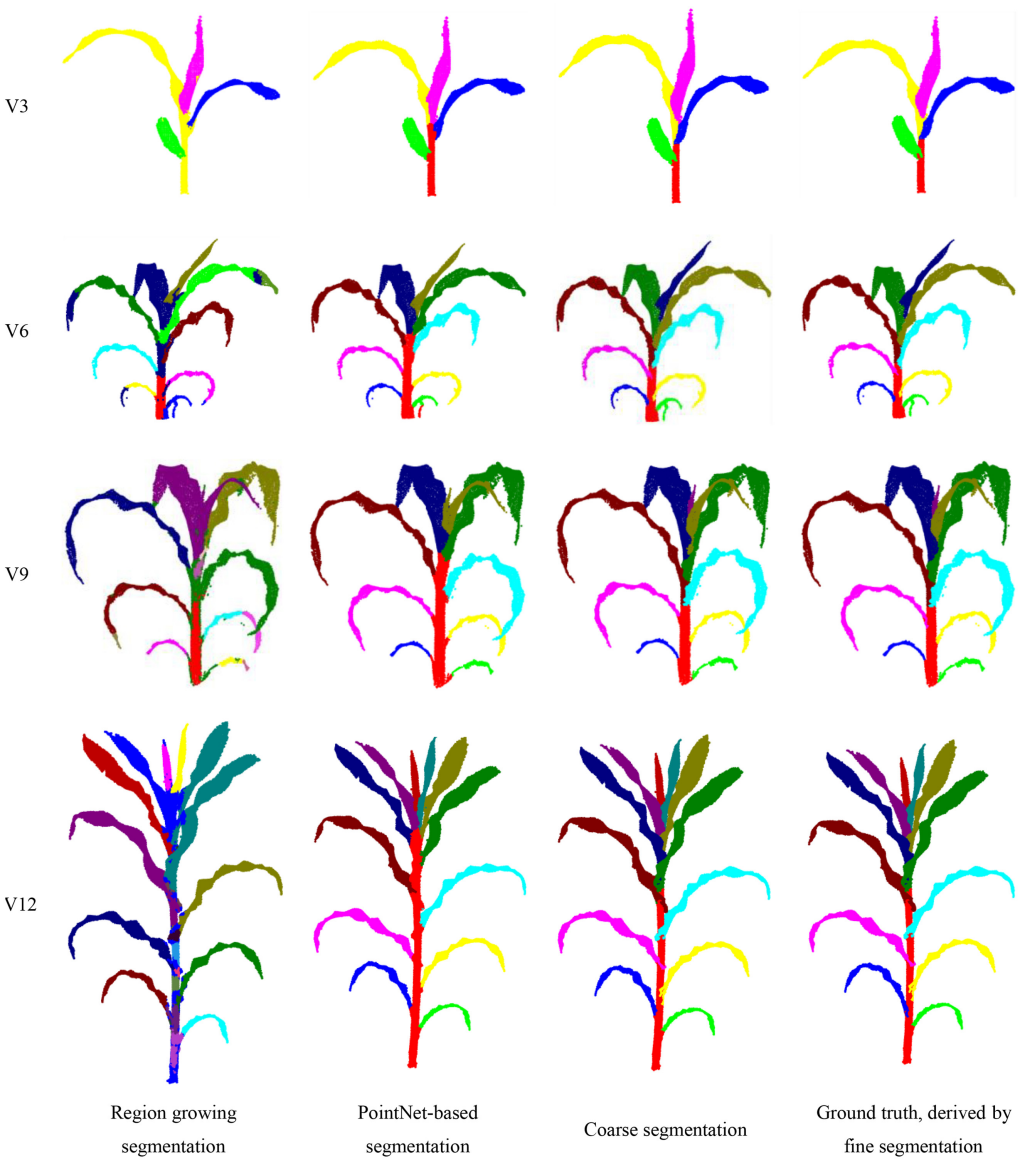
Visualization of segmentation results using region growing, PointNet, coarse segmentation, and fine segmentation.

Method comparison was conducted to evaluate the algorithm performance in coarse segmentation. The point cloud data used here consisted of 12 shoots obtained from the 3D scanner (mentioned in the data acquisition section). Region growing in Point Cloud Library (PCL) [51] and PointNet-based segmentation are considered the state-of-the-art methods for comparison. The best segmentation result was obtained through parameter exhaustion for each shoot using region growing. For PointNet-based segmentation [52], a training dataset containing 1,000 labeled maize shoots was built using Label3DMaize. The PointNet model was then trained, and the segmentation model was derived. The segmentation accuracy is reported in Table 4, and representative results of each growth stage are shown in Fig. 7. The fine segmentation results derived using Label3DMaize were regarded as the well-segmented reference for comparison. Results showed that Label3DMaize could deal with MVS reconstructed point clouds and also handle the point cloud derived using 3D scanner. Region growing is oriented to solve general segmentation problems; the segmentation effect is obviously different from the other 2 methods in maize point cloud segmentation. Thus, the efficiency of region growing is less than that of PointNet and coarse segmentation. The segmentation result of coarse segmentation presented in this article is more accurate than that of PointNet. Although the PointNet model can realize automatic segmentation compared with the rough segmentation containing interaction in this article, dealing with many details could be challenging. For instance, it is difficult to accurately extract the point cloud at the stem and leaf boundary, segmenting a big leaf wrapping a small leaf at the shoot top could be challenging, and it always ignores the newly emerged leaves.

**Table 4:** Accuracy comparison of region growing, PointNet, and coarse segmentation

	Overall accuracy (%)	Precision (%)	Recall (%)	Micro-F1 (%)	Macro-F1 (%)
Region growing	79.1	74.7	75.3	76.8	80.5
PointNet	92.6	92.6	92.6	91.7	90.7
Coarse segmentation	99.2	99.0	99.1	99.0	99.0

### Performance on other plants

This study determined the performance of Label3DMaize in segmenting other plants with only 1 main stem, including tomato, cucumber, and wheat.

**Figure 8:**
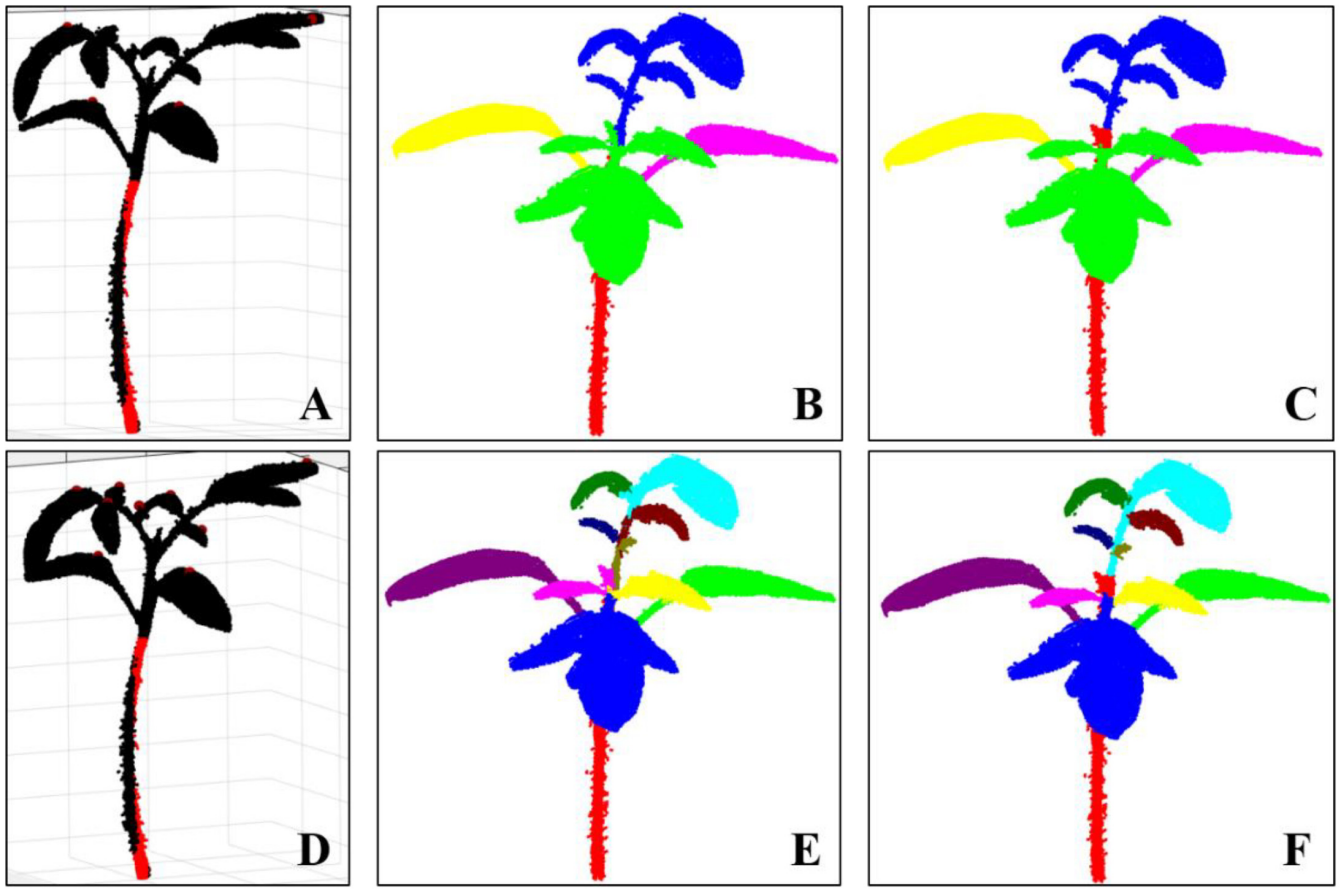
Performance evaluation of Label3DMaize on tomato for 2 types of segmentation. Type I: leaf cluster segmentation (A–C). Type II: individual leaf segmentation (D–F). A and D illustrate the highest point selection in the 2 forms of coarse segmentation. B and E show the coarse segmentation results. C and F are fine segmentation results

Two types of segmentation have been conducted on tomato in the literature [11]; the first (Type I) treats a big leaf with several small leaves as a cluster leaf, while the second (Type II) treats each big or small leaf as independent. This study aimed to realize these 2 type forms using Label3DMaize. The Type I segmentation result (Fig. 8B) was derived by selecting the highest point of each leaf cluster (Fig. 8A) in the coarse segmentation procedure and details were enhanced by fine segmentation (Fig. 8C). For Type II segmentation, the highest points of all the leaves have to be specified (Fig. 8D). Consequently, coarse and fine segmentation could be derived (Fig. 8E and F). The segmentation method used by Ziamtsov and Navlakha [11] is based on a machine learning model; thus, it can only segment trained plants. In contrast, Label3DMaize has better generality.

**Figure 9:**
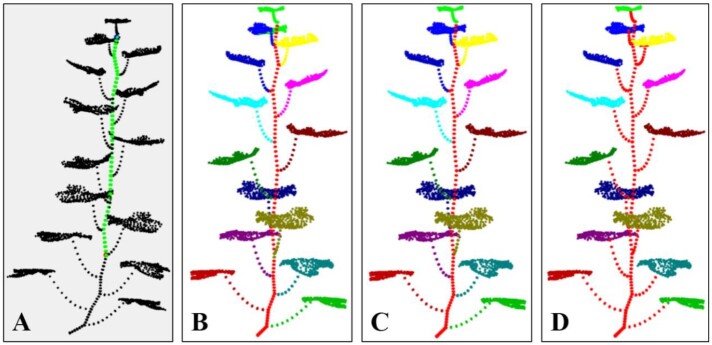
Visualization of cucumber point cloud segmentation. (A) Illustration of the lowest and highest selection points in stem segmentation. (B) Coarse segmentation result. (C) Fine segmentation. Each leaf and corresponding petiole are classified as an instance. (D) Fine segmentation. All the petioles and the main stem are classified as an instance.

Cucumber was selected as a plant representative to test the segmentation performance of Label3DMaize on plants with a soft stem. Different from the topological structure of maize, cucumber has larger stem curvature and has leaf petioles. Thus, the interactive end point selection for stem segmentation of cucumber differs from that in maize. Selection of the highest point of cucumber stem is similar to that in maize. When selecting the other stem end point, we could find the lowest point that coincides with the straight-line direction from the stem top to bottom (Fig. 9A). Although the unselected stem part will be missing, it can be completed in the subsequent coarse segmentation (Fig. 9B). The coarse segmentation and directly fine segmentation tend to segment each leaf and corresponding petiole into an individual organ (Fig. 9C). The separated petiole and leaf can be obtained by fine segmentation, which segments all the petioles and a single stem as a whole (Fig. 9D).

**Figure 10:**
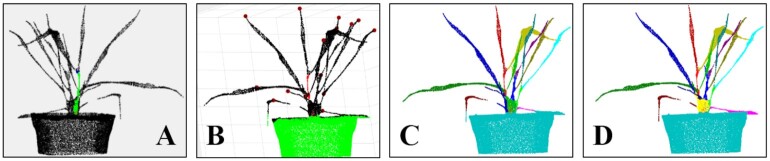
Visualization of wheat shoot segmentation using Label3DMaize. (A) Stem point selection. (B) Selection of highest points in leaves and tillers. (C) Coarse segmentation. (D) Fine segmentation.

A point cloud of wheat shoot at the early growth stage was acquired using the MVS-Pheno platform. Because the wheat shoot is small with a thin stem, the tiller points are fused together near the shoot base. However, the tiller tops could be identified, which enables segmentation of the wheat shoot by Label3DMaize. For plants with tillers, only 1 stem is selected in the stem segmentation procedure (Fig. 10A). When selecting the organ's highest points in coarse segmentation, not only the highest point of each leaf but also the highest point of each tiller has to be selected (Fig. 10B). Coarse segmentation can ensure a better effect of leaf segmentation (Fig. 10C). However, tillers and stem are prone to undersegmentation, which needs to be adjusted by fine segmentation (Fig. 10D).

## Discussion

### Shoot-organ point cloud segmentation

In representative shoot-organ segmentation approaches [36], leaf overlap challenges shoot segmentation, especially for upper leaves in compact shoot architecture. Once the segmentation is complete, it is difficult to correct the false segmentation points. Although commercial software, such as Geomagic Studio, can solve this problem, it is complicated and time-consuming. In contrast, the Label3DMaize toolkit integrates a top-down segmentation algorithm and interactive operations according to the morphological structure of maize shoots, which can realize semi-automatic fine point cloud segmentation. The top-down coarse segmentation ensures topological accuracy, and the interactive operations improve the segmentation accuracy and details. Although coarse segmentation can meet the basic demand for phenotype extraction, it is not satisfactory for high-precision phenotypic analysis and 3D reconstruction based on point clouds. In contrast, fine segmentation is more satisfactory for the latter demands. The toolkit can solve the point cloud segmentation problem of compact architecture or organ-overlapping shoots. Although skeleton extraction methods [34, 35] also provide an interactive way to improve the segmentation accuracy, they offer skeleton interaction, which hardly improves the segmentation point details.

Because 3D point cloud annotation tools for plants are lacking, researchers segment plants through multi-view image labeling, deep learning–based image segmentation, MVS reconstruction, and a voting strategy [53]. However, these methods cause a lot of organ occlusion from different view angles; thus, it is hard to segment plants with multiple organs through image labeling and MVS reconstruction. Jin et al. [37] transformed point cloud data into a voxel format, constructed a training set containing 3,000 maize shoots via data enhancement, and proposed a voxel-based convolutional neural network to segment stem and leaf point cloud of maize shoots. Label3DMaize enables researchers to directly handle 3D point cloud segmentation and data annotation without transforming point cloud data into the voxel form. Meanwhile, using the acquired data directly improves the diversity of training set data, rather than by data enhancement, and can thus improve the robustness of the learned model. In addition, label3DMaize can separate the tassel and ear except for the stem and leaf, facilitating phenotype extraction of the tassel (such as the number of tassel branches or the compactness of the tassel) and ears (such as the ear height).

### Practicability of Label3DMaize

In our recent works, the MVS-Pheno platform [18] was used to obtain high-throughput 3D point cloud data of maize shoots at different ecological sites for various genotypes and growth stages. However, the underlying knowledge about genotypes and the differences in cultivation management have not been fully explored, indicating that high-throughput phenotypic acquisition is far from practical application. Therefore, it is urgent to establish automatic and online data analysis approaches [54]. However, owing to the complexity of plant morphological structure, it is difficult to realize automatic 3D segmentation from the plant morphological characteristics and regional growth method only. Deep learning is a feasible way to realize automatic segmentation by mining deep features of plant morphology. The greatest challenge in 3D point cloud segmentation by deep learning is the lack of high-precision and efficient data annotation tools. Most of the existing 3D data annotation methods are for voxel data [37, 55], not 3D point clouds. Thus, Label3DMaize provides a practical tool for 3D point cloud data annotation for maize and could be a reference for other plants. It has been demonstrated that the toolkit can segment or label other plants, such as tomato, cucumber, and wheat. Coarse segmentation, i.e., the top-down point cloud segmentation algorithm using optimal transportation distance, suits plants with a single stem. Meanwhile, if a plant has too many organs, selecting all the highest points of each organ is rather complicated. Above all, interactive operations in fine segmentation enable extension of the toolkit to other specific plants. Specifically, Label3DMaize does not depend on data generated through MVS-Pheno. Any point cloud of maize shoot can be the toolkit input, including data acquired using 3D scanners (Fig. 7), or reconstructed from multi-view images acquired by handheld cameras.

Unlike RGB image data annotation [40], data enhancement does not significantly improve the model robustness of 3D point cloud segmentation models. Thus high-quality data annotation is important. It takes 4–10 minutes to label a maize shoot point cloud by Label3DMaize, and this labeling efficiency can meet the needs of constructing a training dataset for deep learning. The fine segmentation module in Label3DMaize ensures accurate segmentation of detailed features at the organ connections and is thus satisfactory for organ-level 3D reconstruction. Of note, coarse segmentation results can be used as the annotation data if high precision of the annotation is not required, thus saving a lot of time.

Label3DMaize is designed for individual shoots and does not support segmentation of multiple maize shoots. Thus, point clouds containing multiple shoots have to be preprocessed into individual shoot point clouds first, through spatial connection property of points, or interactively separated using commercial software (such as CloudCompare or Geomagic Studio). This shoot separation preprocess is easy for scenarios without cross organs. Thus, point cloud data acquisition is important for subsequent segmentation. Point clouds with less noise are required when using Label3DMaize. For shoots with much random noise [35], point cloud denoising should be performed first and then set as the toolkit input for segmentation. Compared with image annotation, the data annotation efficiency of Label3DMaize is still lower, and fine segmentation requires more manual interaction, which has higher requirements for user experience and concentration. Thus the algorithm for Label3DMaize needs improvement to raise the automation level of point cloud segmentation.

### Future work

At present, a large amount of 3D point cloud data of maize shoots has been obtained using MVS-Pheno. In our future study, representative data will be selected and annotated by Label3DMaize, then a 3D maize shoot annotation dataset will be constructed. A deep learning–based point cloud segmentation model will then be developed to realize the automatic segmentation of maize shoots. In addition, ted -aize organ data could be used to build a 3D shape model of maize. All the above technologies or data will conversely simplify the segmentation and labeling processes of the toolkit. Subsequently, online phenotypic extraction and 3D reconstruction of maize shoot algorithms will be studied using the well-segmented point clouds. The segmentation algorithm and this toolkit will be extended to other crops according to their morphological characteristics, which will promote the automatic 3D point cloud segmentation of plants.

## Availability of Supporting Source Code and Requirements

- Project name: Label3DMaize Toolkit
- Project home page: https://github.com/syau-miao/Label3DMaize.git
- Source code and executable program: [57]
- Operating systems: Windows
- Programming languages: MATLAB
- License: GNU General Public License (GPL)
- RRID:SCR_021029
- biotools ID: label3dmaize

## Data Availability

The data underlying this article and snapshots of our code are available in the GigaDB repository [56].

## Additional Files

**Supplementary Program**. Executable program of Label3DMaize, which requires that MATLAB runtime (Version 9.2 or above) be installed.

**Supplementary Data S1:** The point clouds of maize shoots described in Fig. 6, including the point clouds acquired using MVS-Pheno, coarse segmentation results, fine segmentation results, and sample-based segmentation results.

**Supplementary Data S2:** Point cloud data described in Fig. 7. These point clouds are acquired using a 3D scanner.

**Supplementary Data S3:** Segmentation results on other plants, including tomato data described in Fig. 8, cucumber data described in Fig. 9, and wheat data described in Fig. 10.

## Abbreviations

CPU: central processing unit; MRF: Markov random fields; MVS: multi-view stereo; PCL: Point Cloud Library; R2: blister stage; SVM: support vector machine; V6: sixth leaf stage; V9: ninth leaf stage; V13: 13th leaf stage.

## Competing Interests

The authors declare that they have no competing interests.

## Funding

This work was partially supported by Construction of Collaborative Innovation Center of Beijing Academy of Agricultural and Forestry Sciences (No. KJCX201917), Science and Technology Innovation Special Construction Funded Program of Beijing Academy of Agriculture and Forestry Sciences (No. KJCX20210413), the National Natural Science Foundation of China (No. 31,871,519, No. 32,071,891), Reform and Development Project of Beijing Academy of Agricultural and Forestry Sciences, China Agriculture Research System (No. CARS-02).

## Authors' Contributions

T.M. performed the major part of methodology in Label3DMaize and developed the toolkit. W.W. improved the methodology. W.W. wrote and revised the manuscript. S.W. and C.Z. acquired the point cloud data and performed methodology comparison. Y.L. evaluated the performance of the toolkit and conducted PointNet–based segmentation for comparison. W.W. and X.G. applied for funding support. X.G. proposed the demand and designed this study, and participated in writing the manuscript. All authors read and approved the final manuscript.

## Supplementary Material

giab031_GIGA-D-20-00335_Original_Submission

giab031_GIGA-D-20-00335_Revision_1

giab031_GIGA-D-20-00335_Revision_2

giab031_Response_to_Reviewer_Comments_Original_Submission

giab031_Response_to_Reviewer_Comments_Revision_1

giab031_Reviewer_1_Report_Original_Submission Chris Armit -- 12/18/2020 Reviewed

giab031_Reviewer_2_Report_Original_Submission Dong Chen -- 12/30/2020 Reviewed

giab031_Reviewer_3_Report_Original_Submission Xiaopeng Zhang -- 12/31/2020 Reviewed

giab031_Reviewer_4_Report_Original_Submission Luis Diaz-Garcia -- 1/4/2021 Reviewed

giab031_Reviewer_4_Report_Revision_1 Luis Diaz-Garcia -- 3/24/2021 Reviewed

giab031_Supplemental_Files
