# Supplementary material for: Label3DMaize: toolkit for 3D point cloud data annotation of maize shoots
Source: Gigascience. 2021 May 7;10(5):giab031. doi: 10.1093/gigascience/giab031 (PMC8105162; doi:10.1093/gigascience/giab031)
Supplement: giab031_GIGA-D-20-00335_Original_Submission [file giab031_giga-d-20-00335_original_submission.pdf]

GigaScience

Label3DMaize: toolkit for 3D point cloud data annotation of maize shoots

--Manuscript Draft--

**Manuscript Number:**	GIGA-D-20-00335
**Full Title:**	Label3DMaize: toolkit for 3D point cloud data annotation of maize shoots
**Article Type:**	Research

Funding Information:

Construction of Collaborative Innovation Center of Beijing Academy of Agricultural and Forestry Sciences (KJCX201917)	prof. Xinyu Guo
Science and Technology Innovation Special Construction Funded Program of Beijing Academy of Agriculture and Forestry Sciences	Dr. Weiliang Wen
National Natural Science Foundation of China (31871519)	prof. Xinyu Guo
Reform and Development Project of Beijing Academy of agricultural and Forestry Sciences	Not applicable
China Agriculture Research System (CARS-02)	Not applicable
Construction of Scientific Research and Innovation Platform in Beijing Academy of Agriculture and Forestry Sciences (PT2020-24)	prof. Xinyu Guo

Abstract:

Background

Three-dimensional (3D) point cloud is the most direct and effective data form for studying plant structure and morphology. In point cloud studies, the point cloud segmentation of individual plants to organs directly determines the accuracy of organ-level phenotype estimation and the 3D plant reconstruction reliability. However, highly accurate, automatic, and robust point cloud segmentation approaches for plants are unavailable. Thus, the high-throughput segmentation of many shoots is challenging. Although deep learning can feasibly solve this issue, software tools for 3D point cloud annotation to construct the training dataset are lacking.

Results

In this paper, a top-to-down point cloud segmentation algorithm using optimal transportation distance for maize shoots is proposed. On this basis, a point cloud annotation toolkit, Label3DMaize, for maize shoot is developed. Further, the toolkit was applied to achieve semi-automatic point cloud segmentation and annotation of maize shoots at different growth stages, through a series of operations, including stem segmentation, coarse segmentation, fine segmentation, and sample-based segmentation. The toolkit takes about 4 to 10 minutes to segment a maize shoot, and consumes 10%-20% of the total time if only coarse segmentation is required. Fine segmentation is more detailed than coarse segmentation, especially at the organ connection regions. The accuracy of coarse segmentation can reach 97.2% of the fine segmentation.

Conclusion

Label3DMaize integrates point cloud segmentation algorithms and manual interactive operations, realizing semi-automatic point cloud segmentation of maize shoots at different growth stages. The toolkit provides a practical data annotation tool for further online segmentation researches based on deep learning and is expected to promote automatic point cloud processing of various plants.

Corresponding Author: Xinyu Guo, Ph.D.
Beijing Research Center for Information Technology in Agriculture: National

Powered by Editorial Manager® and ProduXion Manager® from Aries Systems Corporation

	Engineering Research Center for Information Technology in Agriculture<br>Beijing, CHINA
**Corresponding Author Secondary Information:**	
**Corresponding Author's Institution:**	Beijing Research Center for Information Technology in Agriculture: National Engineering Research Center for Information Technology in Agriculture
**Corresponding Author's Secondary Institution:**	
**First Author:**	Teng Miao
**First Author Secondary Information:**	
**Order of Authors:**	Teng Miao
	Weiliang Wen
	Sheng Wu
	Chao Zhu
	Yinglun Li
	Xinyu Guo, Ph.D.
**Order of Authors Secondary Information:**	
**Additional Information:**	
**Question**	**Response**
Are you submitting this manuscript to a special series or article collection?	No
**Experimental design and statistics**<br><br>Full details of the experimental design and statistical methods used should be given in the Methods section, as detailed in our Minimum Standards Reporting Checklist. Information essential to interpreting the data presented should be made available in the figure legends.<br><br>Have you included all the information requested in your manuscript?	Yes
**Resources**<br><br>A description of all resources used, including antibodies, cell lines, animals and software tools, with enough information to allow them to be uniquely identified, should be included in the Methods section. Authors are strongly encouraged to cite Research Resource Identifiers (RRIDs) for antibodies, model	Yes

Powered by Editorial Manager® and ProduXion Manager® from Aries Systems Corporation

organisms and tools, where possible.<br><br>Have you included the information requested as detailed in our Minimum Standards Reporting Checklist?	
**Availability of data and materials**<br><br>All datasets and code on which the conclusions of the paper rely must be either included in your submission or deposited in publicly available repositories (where available and ethically appropriate), referencing such data using a unique identifier in the references and in the "Availability of Data and Materials" section of your manuscript.<br><br>Have you have met the above requirement as detailed in our Minimum Standards Reporting Checklist?	Yes

Manuscript Click here to access/download;Manuscript;Label3DMaize.docx

Label3DMaize: toolkit for 3D point cloud data annotation of maize shoots

Teng Miao[1, †], Weiliang Wen[2, 3, 4, †], Sheng Wu[2, 3, 4], Chao Zhu[1], Yinglun Li[3, 4], Xinyu Guo[2, 3, 4, *]

[1] *College of Information and Electrical Engineering, Shenyang Agricultural University, Shenyang 110161, China.*
[2] *Beijing Research Center for Information Technology in Agriculture, Beijing 100097, China.*
[3] *National Engineering Research Center for Information Technology in Agriculture, Beijing 100097, China.*
[4] *Beijing Key Lab of Digital Plant, Beijing 100097, China.*
* For correspondence. Email: guoxy73@163.com (Xinyu Guo)
† Co-first authors.

Abstract

Background: Three-dimensional (3D) point cloud is the most direct and effective data form for studying plant structure and morphology. In point cloud studies, the point cloud segmentation of individual plants to organs directly determines the accuracy of organ-level phenotype estimation and the 3D plant reconstruction reliability. However, highly accurate, automatic, and robust point cloud segmentation approaches for plants are unavailable. Thus, the high-throughput segmentation of many shoots is challenging. Although deep learning can feasibly solve this issue, software tools for 3D point cloud annotation to construct the training dataset are lacking. **Results**: In this paper, a top-to-down point cloud segmentation algorithm using optimal transportation distance for maize shoots is proposed. On this basis, a point cloud annotation toolkit, Label3DMaize, for maize shoot is developed. Further, the toolkit was applied to achieve semi-automatic point cloud segmentation and annotation of maize shoots at different growth stages, through a series of operations, including stem segmentation, coarse segmentation, fine segmentation, and sample-based segmentation. The toolkit takes about 4 to 10 minutes to segment a maize shoot, and consumes 10%-20% of the total time if only coarse segmentation is required. Fine segmentation is more detailed than coarse segmentation, especially at the organ connection regions. The accuracy of coarse segmentation can reach 97.2% of the fine segmentation. **Conclusion**: Label3DMaize integrates point cloud segmentation algorithms and manual interactive operations, realizing semi-automatic point cloud segmentation of maize shoots at different growth stages. The toolkit provides a practical data annotation tool for further online segmentation researches based on deep learning and is expected to promote automatic point cloud processing of various plants.

Key words: Label3DMaize, three-dimensional point cloud, segmentation, maize shoot, data annotation.

1 Introduction

The plant structure and morphology are important features for expressing growth and development. At present many research studies underpin the significance of integrating the three-dimensional (3D) morphological characteristics of plants when conducting genetic mapping, adaptability evaluation, and crop yield analysis [1, 2]. Using the 3D data acquisition technology to obtain a 3D point cloud is the most effective way to perceive the plant structure and morphology digitally. However, 3D point clouds are initially obtained in an unordered, unstructured manner and with little semantic information. Therefore, it is critical to use computer graphics technologies and plant morphology knowledge to convert the unstructured 3D point clouds into well-organized and structured data that contains rich morphological features with semantic information. Therefore, plant morphology research based on measured point clouds forms a critical

component of 3D plant phenomics [3-5], 3D plant reconstruction [2, 6], and functional-structural plant models (FSPMs) [7, 8].

The development of 3D data acquisition technology has significantly enriched approaches for fine-scale 3D data acquisition of individual plants, including 3D scanning [9, 10], LiDAR [11], depth camera [12], time of flight (ToF) reconstruction [13], and multi-view stereo (MVS) reconstruction [14, 15]. Owing to the low cost of sensors and better quality of reconstructed point clouds, MVS reconstruction have been widely adopted in many applications. Recently, multi-view image acquisition platforms that can realize semi-automatic and high-throughput 3D data acquisition for individual plants have been developed [16] and enable 3D data acquisition for the phenotypic analysis of large-scale breeding materials [17, 18]. However, how to efficiently and automatically process the acquired big data of 3D point clouds is a bottleneck in 3D plant phenotyping.

The key technologies for 3D point cloud data processing include data registration, the region of interest extraction, denoising, segmentation, feature extraction, and mesh generation. Among these tasks, point cloud segmentation is challenging. Therefor, automatic and accurate point cloud segmentation could significantly impact subsequent results of phenotype extraction and 3D reconstruction. Point cloud segmentation can be classified as population-shoot or shoot-organ segmentation. Population-shoot segmentation allows for automatic segmentation of maize population under low density [19] or at early growth stages [20, 21] with little overlap, which can be realized via the spatial distance between shoots. However, it is difficult to achieve automatic segmentation of high density populations or with many overlapping organs in late growth stages. Comparatively, more attention has been paid to shoot-organ segmentation. Though high-quality input point clouds and restricted connections between organs are required, color-based [22] and point clustering [23-25] approaches have also been widely used. For instance, Elnashef et al. [14] used the local geometric features of the organs to segment maize leaves and stems at six-leaf stage. Paulus et al. [26, 27] segmented the grape shoot organs by integrating fast point feature histograms (FPFH), support vector machine (SVM), and region growing approaches. However, these methods can only segment plant shoots with clear connection characteristics between stems and leaves [9] and can hardly solve leaf wrapping stem segmentation problems. For time-series 3D point clouds, the leaf multi-labeling segmentation method was used for organ segmentation and plant growth monitoring [28]. While plant organs could also be segmented through skeleton extraction and hierarchical clustering [29, 30], these methods need interactive manual correction for complex plants to guarantee the segmentation accuracy. Jin et al. [31] proposed a median normalized vector growth algorithm that can segment the stems and leaves of maize shoots. On this basis, an annotation dataset of maize shoots was constructed, and the deep learning method was introduced to improve the automatic segmentation level [32]. However, few parameter interactions are still needed for different shoot architecture and cannot meet the needs of high realistic 3D reconstruction.

Due to the complexity of plant morphology and structure, almost all 3D point cloud segmentation methods for plants need certain manual interaction, which is inconvenient for huge amounts of point cloud data processing, and substantially decreases the efficiency. Therefore, it is necessary to improve the automation of segmentation and increase the throughput of 3D point cloud data processing for plants. Deep learning approaches can effectively solve this problem [33, 34], among which the construction of high-quality training data set is a prerequisite. For example, LabelMe [35] can realize high-quality data annotation for image segmentation. However, 3D point cloud tools for data annotation are rare, especially for plants. Besides, the existing datasets for 3D plant segmentation contain only little data [34, 36, 37], which cannot meet the data requirements for high-quality deep learning models.

Since point cloud annotation of plants is labor-intensive and time-consuming, deep learning approaches

can be applied to segment plant point clouds. Hence, how to improve the efficiency of high-quality data annotation and develop supporting software tools is the key to automatic point cloud segmentation of plants by deep learning. To meet this data annotation demand, this study used maize as an example and proposes a top-to-down point cloud segmentation algorithm. Besides, a toolkit for point cloud annotation of maize shoots is developed, which could provide technical support for automatic and high-throughput processing of plant point clouds.

2 Materials and Methods

2.1 Field experiment and data acquisition

Three maize cultivars, including MC670, Xianyu 335 (XY335), and NK815, were planted on May 20th, 2019, at the Tongzhou experimental field of Beijing Academy of Agriculture and Forestry Sciences (116.70°E, 39.71°N). The planting density of all the plots was six plants/m^2 with a row spacing of 60 cm. Morphological representative shoots of each cultivar at 6th leaf (V6), 9th leaf (V9), 13th leaf (V13), and blister (R2) stages [38], were selected and transplanted into pots. Then multi-view images were acquired using the MVS-Pheno platform [16], after which 3D point clouds of the shoots were reconstructed.

2.2 Overview of the segmentation pipeline

The point cloud of a maize shoot can be segmented into five kinds of instances: stem, leaf, tassel, ear, and pot. The stem, tassel, and pot on a shoot can be regarded as an instance for each. For each transplanted shoot at stage R2, assuming that it contains n_1 ears and n_2 leaves, the point cloud of this shoot can thus be segmented into N=3+n_1+n_2 instances. $\emptyset_{\mathrm{u}}$ represents the point cloud to be segmented, and $\emptyset_{\mathrm{s}}^{i}$ $(i = 1, 2, \dots\dots, N)$ represent the ith point cloud instance. In particular, $\emptyset_{\mathrm{s}}^{1}$ and $\emptyset_{\mathrm{s}}^{N}$ refer to the stem and pot (if exists) instance, respectively. Before the segmentation begins, $\emptyset_{\mathrm{u}}$ contains all the points of the shoot, and $\emptyset_{\mathrm{s}}^{i}$ are all empty. With the progression of segmentation, the points in $\emptyset_{\mathrm{u}}$ are gradually assigned to $\emptyset_{\mathrm{s}}^{i}$. The segmentation completes when $\emptyset_{\mathrm{u}}$ is empty.

The segmentation pipeline includes five parts (Figure 1): point cloud down-sampling, stem segmentation, coarse segmentation, fine segmentation, and sample-based segmentation. (1) Point cloud down-sampling. The original input point cloud is down sampled to maintain the shoot morphological features, which improves the segmentation efficiency and quickens the entire segmentation process. (2) Stem segmentation. The top and bottom points of the stem are interactively selected, and the corresponding radius parameters are interactively adjusted. Subsequently, the median region growing is applied to segment the stem points from the shoot automatically. (3) Coarse segmentation. The highest points of each organ instance, except the stem, are obtained via automatic calculation or manual interaction, after which all organ instances are segmented automatically based on the optimal transportation distances. (4) Fine segmentation. The unsatisfactory segmentation point regions are selected interactively, and the seed points of organ instances are selected. Organs are then segmented by Markov random fields (MRF). (5) Sample-based segmentation. Maize shoots with high-resolution point clouds are segmented based on the fine segmentation result of low-resolution point clouds.

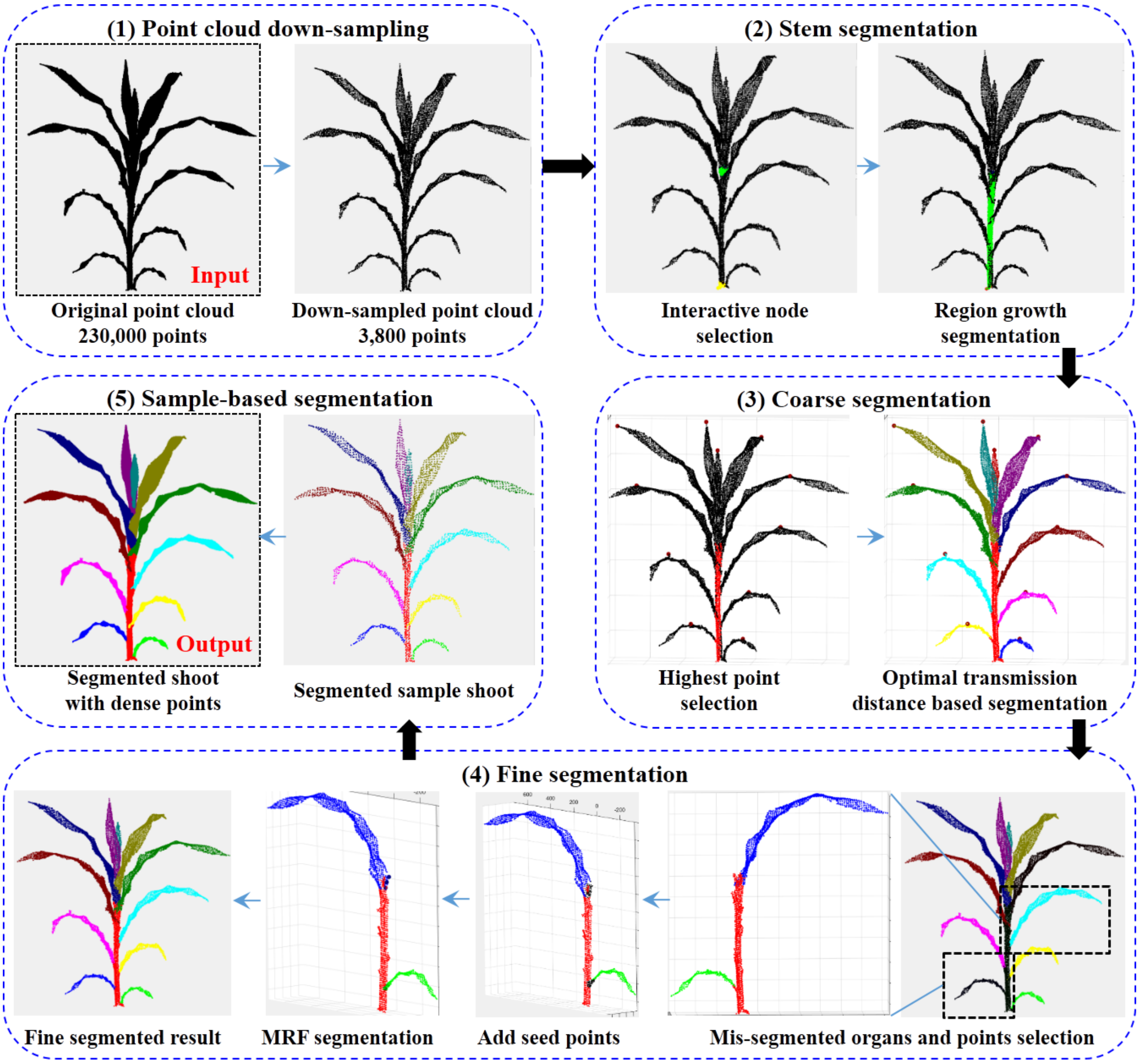

Figure 1: workflow of the segmentation

2.3 Stem segmentation

Two seed points s_0 and s_n at the bottom and top of each stem, were selected interactively. Then, a median-based region growing algorithm [31] was applied to segment the stem points. This segmentation procedure update the seed point iteratively along the direction from s_0 to s_n. Points around the seed points were classified into stem points. Suppose the algorithm is currently at the kth iteration, and the seed point is s_k, the segmentation process was evaluated as follows:

Step1: Points lying in a sphere were classified as stem points, where s_k is the center of the sphere, r_1 is its radius, and r_1 is a user-specified parameter.

Step 2: The growth direction $\vec{v_k}$ was determined according to:

$$\vec{v_k} = (\alpha\vec{v_1} + \beta\vec{v})/\|\alpha\vec{v_1} + \beta\vec{v}\|_2$$

$$\vec{v_1} = median\{(p_A - s_k)/\|p_A - s_k\|_2, p_A \in A\}$$

$$\vec{v} = (s_n - s_k)/\|s_n - s_k\|_2$$

In this formula, $\|\ \|_2$ is L_2 normal form, and median{} represents the median operation. α and β are weight parameters set by users and $\vec{v_1}$ is the normalized vector from the median of already segmented points of the stem to the seed point s_k. Meanwhile, $\vec{v}$ is the normalized vector from s_k to s_n, which

corrects the growth direction to coincide with the stem. In practice, $\alpha = 0.2$ while $\beta = 0.8$. This parameter setting ensures that the stem points can be correctly segmented under different r_1 values, during the entire growing process.

Step 3: A new seed point s_{k+1} for the next iteration was estimated according to $s_{k+1} = s_k + r_1\overrightarrow{v_k}$.

Step 4: Region growing finish condition judgement. Supposing L represents the line segment from s_0 to s_n, then project s_{k+1} on L. If the projection point was not on L, it indicated that the current regional growth was beyond the stem region, and the iteration should be stopped. Otherwise, continue the k+1 times iteration and execute step 1.

Because the maize stem gradually thins from bottom to top, a uniform radius r_1 may generate over segmentation, i.e., classifying the points of other organs into the stem. Besides, the region growing algorithm also over segments points in some regions at the bending of the stem. Therefore, a simple median operation was adopted to eliminate the over segmented points. First, the already segmented stem points were evenly divided into M segments along the direction of $(s_n - s_0)/\|s_n - s_0\|_2$, and the median axis of each segment was fitted using the least squares. The average distance from each point to the central axis was then calculated. If the distance from a point to the central axis was less than the average distance, it was retained as the stem point; otherwise it was removed from the stem to the unsegmented point set. Users can perform the median operation several times in the toolkit to reduce the over-segmentation problem. Although multiple median operations cause an under segmentation of stem point cloud, the issue is resolved in the subsequent organ segmentation processes. $\emptyset_s^1$ represents the segmented stem points, and these points are removed from $\emptyset_u$. Subsequent organ segmentation is performed in the remaining point cloud. Stem point cloud segmentation is illustrated in Figure 2.

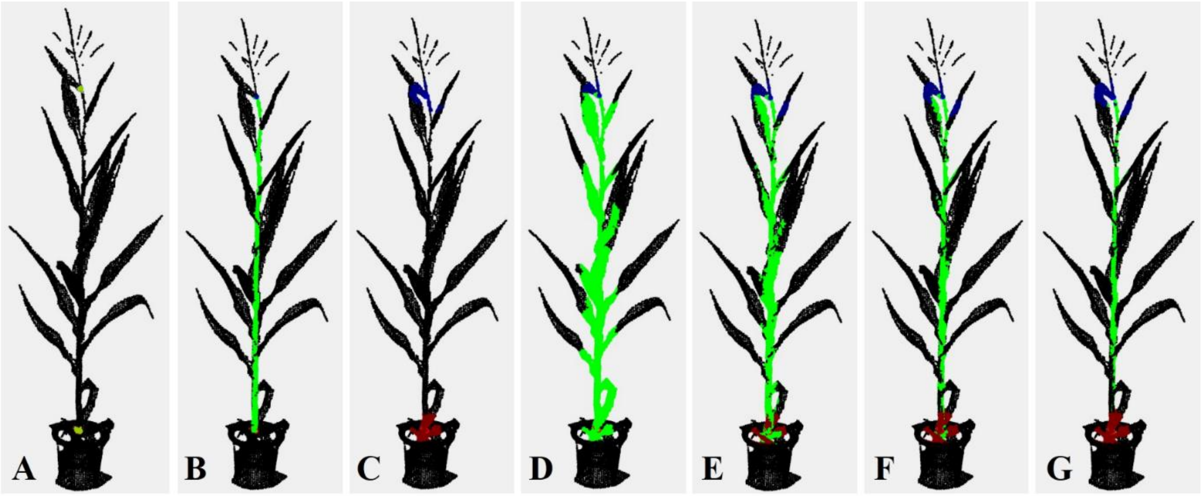

Figure 2: Stem point cloud segmentation. (A) Seed points at the bottom and top of the stem are interactively selected, and an appropriate segmentation radius is set. (B) Stem segmentation result based on (A). (C) A big radius is set. (D) Segmentation result based on (C). (E)-(G) Stem segmentation results with 1, 2, and 3 median operations based on (D).

2.4 Shoot alignment

The shoot points were transformed into a regular coordinate system to access the position of each point in the cloud conveniently. The midpoint of the already segmented stem point cloud was taken as the origin O of the new shoot coordinate system. In contrast, the Z-axis of the new coordinate system was the middle axis estimated by the least squares method from the stem point cloud. Then, the shoot point cloud was projected onto the plane using the Z-axis as its normal vector. The first and second principal component vectors of the projection points were determined by principal component analysis (PCA) and assigned as the

X and Y-axis of the new shoot coordinate system, respectively. Subsequently, the original point cloud coordinates were transformed into the new shoot coordinate system, and the coordinates of their *z* value judged the height of points in the shoot. Points are higher with greater *z* values.

2.5 Coarse segmentation of organs

A top-to-down point cloud segmentation algorithm for maize organs from a shoot was applied. The highest point of each organ was taken as the seed point of the organ (Figure 3A). The other shoot points after stem segmentation were classified into corresponding organ instances from top to down by the optimal transportation distances (Figure 3B).

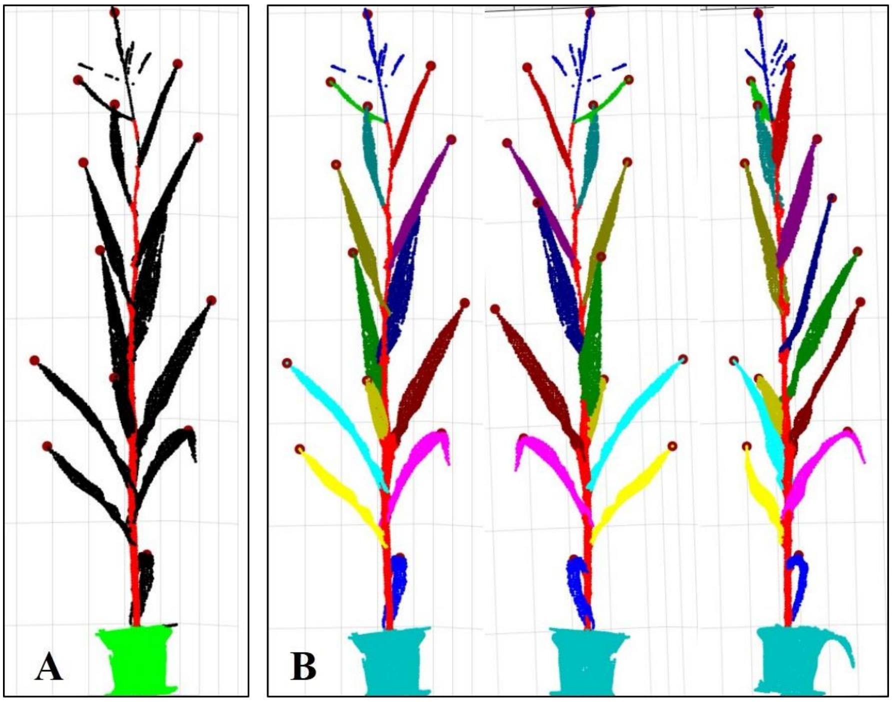

Figure 3: Illustration of coarse segmentation. (A) Highest point determination of each organ. (B) Visualization of segmented shoot from different angles of view.

2.5.1 Organ seed points determination

After stem segmentation, the point cloud of maize shoots was spatially divided into several relatively discrete organs (excluding the stem). The highest point of each organ was regarded as the seed point (Figure 3A). If a pot was involved in the point cloud, all points with a *z* value less than the lowest point of the stem were directly classified as pot points. Usually, the highest point of a new leaf appears at the tip region; the middle and lower fully unfolded leaves are mostly curved. Meanwhile, the highest point lies in the middle of the leaf, and the highest points of a tassel or ear are at the top. Therefore, the highest point of each organ was determined by searching for the point with the maximum *z* value.

For any point *p* of an organ, we searched its neighbors within a radius of r_2. If the *z* value of point *p* was greater than that of its neighboring points, the point was regarded as the highest in the organ. The parameter r_2 actually affects the recognition of the highest point of an organ. Too small r_2 may cause the highest point found being the local highest point, rather than the global highest point of the current organ. Therefore, r_2 was set 1.5 times of the leaf width by default, which allows users to set interactively according to the morphological characteristics of the target shoot.

Numerical experiments show that the highest points of most organs can be derived by setting the appropriate r_2. However, the algorithm still has two problems. (1) When the distance between the highest points of adjacent organs in the shoot vary significantly, it is difficult to find a suitable r_2 to calculate all complete and accurate highest points. For example, in some shoots, the highest points distance between new emerging leaves is relatively close, while this distance between other leaves is relatively far. (2) Due to the tassel branching structure, each branch has the highest point; multiple highest points of a tassel will be detected using the same algorithm and settings, to ensure the highest points are correctly estimated in other organs. If to ensure only one highest point is calculated in the tassel, the highest point of other organs may be lost.

To solve the problem that the calculation of the highest point of organs may not be accurate, Label3DMaize provides a manual interaction module to modify the highest seed point of each organ. Simultaneously, this operation can also assign a serial number to each organ for further output. Because the number of maize organs is relatively small, this interactive correction operation is convenient and acceptable. The derived seed points of each organ are set into the corresponding instance point cloud $\emptyset_s^i$. At this time, each leaf, tassel, and ear instance point cloud only contains the highest point, and there are multiple points in the pot and stem instances.

2.5.2 Coarse segmentation based on optimal transportation distances

After obtaining the seed points of all the instances, the left points in $\emptyset_u$ were traversed one by one to determine the instance to which they belong. For each point to $\emptyset_u$, the distance between the point and each other point cloud instance were evaluated, and it was classified into the nearest instance. The classified points were evaluated from top to bottom; that is, the points with bigger *z* coordinates were evaluated preferentially. The process was as follows:

Step 1: The points in the point set $\emptyset_u$ were reordered from big to small according to their *z* values.

Step 2: For point $p \in \emptyset_u$, the organ instance it belongs to was determined. The distance d^i from point *p* to the ith instance was defined as

$$d^i = D_s(p, \widetilde{p^i})$$

Where D_s is the optimal transportation distance between any two points calculated based on the sinkhorn algorithm [39] . Then point *p* is assigned into the organ instance with the lowest d^i. $\widetilde{p^i}$, in the ith instance, is the nearest neighbor of point *p* under the optimal transportation distance.

Step 3: Move point *p* from $\emptyset_u$ into the corresponding $\emptyset_s^i$. Continue traversing the next point in $\emptyset_u$, and perform step 2 until $\emptyset_u$ is empty.

Detailed description of D_s in step 2 is explained here. The optimal transportation strategy of point cloud Q to its identical set Q' is that transmit all the quality of any point $p \in Q$ to the same point $p' \in Q'$. The Sinkhorn algorithm [39] was used here to calculate the optimal transportation distances. It allocates the quality of any point $p \in Q$ to all points in Q'. A point with higher allocation quality suggests the point is closer to *p* than any other points under the optimal transportation strategy. Suppose that point cloud Q contains N_Q points. Q' represents the same point set of Q. p_u is the uth point in Q, and M_u indicates the quality of point p_u. Similarly, p'_v is the vth point in Q', and M'_v indicates the quality of point p'_v. m_{uv} represents the transported quality from $p_u \in Q$ to $p'_v \in Q'$. Then the optimal transportation energy from point cloud Q to point cloud Q' can be described as:

$$\underset{m}{\operatorname{argmin}} \sum_{u=1}^{N_Q} \sum_{v=1}^{N_Q} m_{uv} \| p_u - p'_v \| + \frac{1}{\varepsilon} \sum_{u=1}^{N_Q} \sum_{v=1}^{N_Q} m_{uv} \log m_{uv}$$

$$\text{s.t. } m_{uv} > 0; \sum_{v=1}^{N_Q} m_{uv} = M_u ; \sum_{u=1}^{N_Q} m_{uv} = M'_v$$

In this equation, ε is the adjusting parameter, which was set to 5 in this paper, and $\| \quad \|$ is the L_2 normal form. The above equation can be solved by Sinkhorn's matrix scaling algorithm [40], and the optimal transportation from Q to Q' can be derived, that is, an $N_Q \times N_Q$ optimal transportation matrix M is obtained. The element m_{uv} at *u* row and *v* column in the matrix is the transported quality from the uth to the vth point. A larger m_{uv} indicates that the two points are closer. After obtaining the optimal transportation solution, the optimal transportation distance from the uth to the vth point in the point cloud can be defined as $D_s(p_u, p_v) = \frac{1}{m_{uv}}$.

In the optimal transportation energy equation, when parameter ε increases, the transportation strategy gets closer to the classical optimal transportation, and the segmentation result using optimal transportation distance D_s is also closer to that using Euclidean distance. The same results can be derived using the two distances when the ε is greater than 100. When ε is smaller, the solution becomes smoother, and the nearest neighbour calculated under the D_s distance tends to the region with higher point density. Compared with the Euclidean distance, using the optimal transportation distance to estimate the distance between points can better deal with the challenge of big leaves wrapping on leaflets than using the Euclidean distance (Figure 4A and B). When the adhesion area of the two organs is not significantly large, the segmentation results using the optimal transportation distance is better than that of the Euclidean distance (Figure 4C and D).

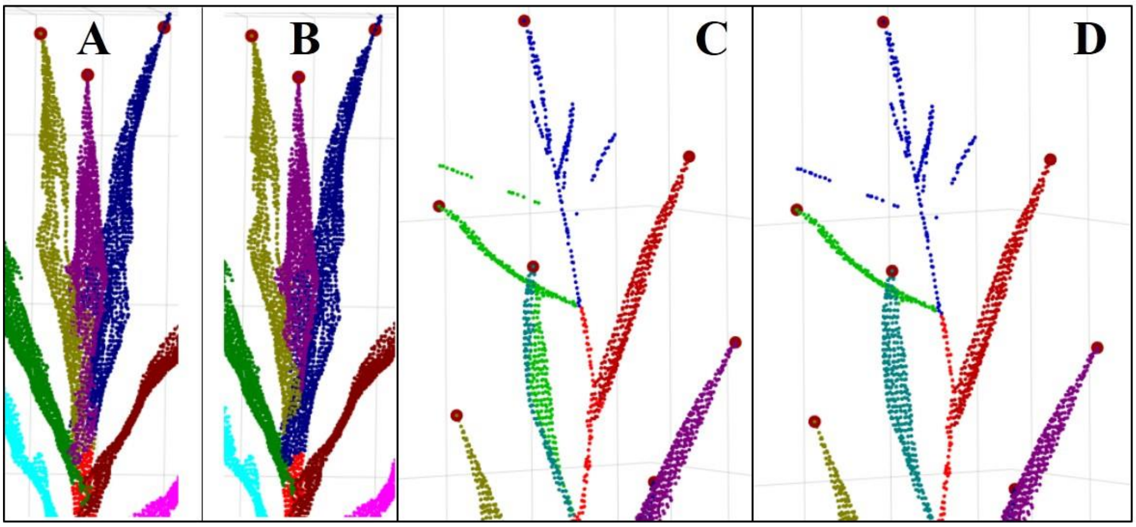

Figure 4: Organ segmentation Comparison using optimal transportation distance and Euclidean distance. Point cloud segmentation result for big leaf wrapping small leaf base case using Euclidean distance (A) and optimal transportation distance (B). Point cloud segmentation result for close or slight organ adhesion case using Euclidean distance (C) and optimal transportation distance (D).

2.6 Fine segmentation of organs

Coarse segmentation can provide preliminary results but false segmentation is frequently observed in the intersecting regions of organs. To obtain more precise segmentation results, this study developed a fine segmentation module for organs in Label3DMaize, which included the following processes:

Step 1: n (n>1) organ instances to be fine segmented were selected, and $\emptyset_{s'}^{i}$ represents the ith instance.

Step 2: The region of interest was selected among the above instance point cloud, represented by $\emptyset_{s'}^{i}$.

Step 3: The seed point for the ith instance $\emptyset_{s'}^{i}$ was selected from region $\emptyset_{u'}$. The selected points were

removed from $\emptyset_{u'}$ and stored in $\emptyset^{i}_{s'}$.

Step 4: The points in $\emptyset_{u'}$ were re-segmented using Markov Random Fields (MRF).

The re-segment algorithm was detailed using MRF in step 4, as explained in the following. The fine segmentation of the interest region mentioned above is a multi-classification problem. It allocates $p_u \in \emptyset_{u'}$ into *n* organ instances $\emptyset^{i}_{s'}$, i.e. search for the right organ tag for point p_u. Hence a mapping function $f_n(p_u)$ is defined for any point p_u. When a point p_u is mapped to the ith instance, $f_n(p_u) = i$, the energy function is defined as:

$$E(f_n) = \gamma \sum_{p_u \in \emptyset_{u'}} D_{p_u}\big(f_n(p_u)\big) + \sum_{(p_u,q_u) \in \aleph(p_u)} V(f_n(p_u), f_n(q_u))$$

$$D_{p_u}(i) = D\big(p_u, \emptyset^{i}_{s'}\big) \quad i = [1,2 \ldots\ldots n]$$

$$V\big(f_n(p_u), n(q_u)\big) = \left(\frac{d(p_u,q_u)}{d'}\right)^{\tau}\left(\frac{a(n_p,n_u)}{\pi}\right)^{\varphi}$$

In this function, $\aleph(p_u)$ is the *k*-neighborhood of $p_u \in \emptyset_{u'}$. The data item $D_{p_u}\big(f_n(p_u)\big)$ measures the loss of classifying p_u to *n* instances $\emptyset^{i}_{s'}$. $D(p_u, \emptyset^{i}_{s'})$ represents the distance from point p_u to instance $\emptyset^{i}_{s'}$, which is the distance from p_u to the nearest point in $\emptyset^{i}_{s'}$. γ is a weight parameter that controls the proportion of distance term in the energy function. The smooth item $V(f_n(p_u), f_n(q_u))$ quantifies the corresponding loss when assigning the tag $f_n(p_u)$ and $f_n(q_u)$ for point p_u and q_u, respectively. This smooth term encourages spatial consistency; that is, the probability that adjacent points belong to the same class is higher. The smooth term is composed of the product of the distance term on the left and the angle term on the right. Meanwhile, $d(p_u, q_u)$ is the Euclidean distance of the two points and d' is the maximum Euclidean distance between all points and their neighbourhood points, regulating the distance term in the range of (0, 1]. n_p and n_u are the normal vectors of points p_u and q_u, respectively. $a(n_p, n_u)$ is the angle between the two normals. τ and φ are the weight parameters for the distance and angle term, respectively, both with a default value of 1.0. The minimum solution of the energy function is solved by α-expansion MRF [41].

2.7 Sample-based segmentation

It is suggested that the number of points per shoot should be less than 15000 to ensure data processing efficiency. Therefore, Label3DMaize provides point cloud simplification and sample-based segmentation modules. Voxel-based simplification is adopted in the toolkit. Sample-based segmentation refers to the automatic segmentation of dense point cloud via the segmentation result of the corresponding simplified point cloud. Specifically, suppose that point cloud *A* is the simplification of dense point cloud *B*, and *A* has already been segmented while *B* is to be segmented. Calculating the *k*-nearest neighbors in *A* of any point $p \in B$, and then counts how many points of these *k*-nearest neighbors belong to each instance. The instance with the maximum neighbour points is determined as the instance of point *p*.

3 Results

3.1 Interface and operations of Label3DMaize

The Label3DMaize toolkit interface is composed of the main interface and multiple sub-interfaces, including stem segmentation, coarse segmentation, fine segmentation, and sample-based segmentation (Figure 5). Each sub-interface is popped up after the corresponding button on the main interface is triggered. The main interface and each sub-interface are composed of an embedded dialog and an interactive visual window (only the embedded dialog in each sub-interface is shown in Figure 5). The interactive visual window enables the users to rotate, zoom, translate, select interested points in the view, and improve the segmentation

effect visually and interactively. The input of the toolkit includes point cloud files in text format, such as txt or ply. According to the operational process shown in Figure 5, segmentation results can be refined step by step by inputting parameters and manually selecting points. The output of the toolkit is a text file with annotation information; that is, each 3D coordinate point in the text has a classification identification number, and the points with the same identification number belong to the same instance. This format files are applicable for 3D deep learning of maize shoots. The executable program of Label3DMaize can be found in the attachment.

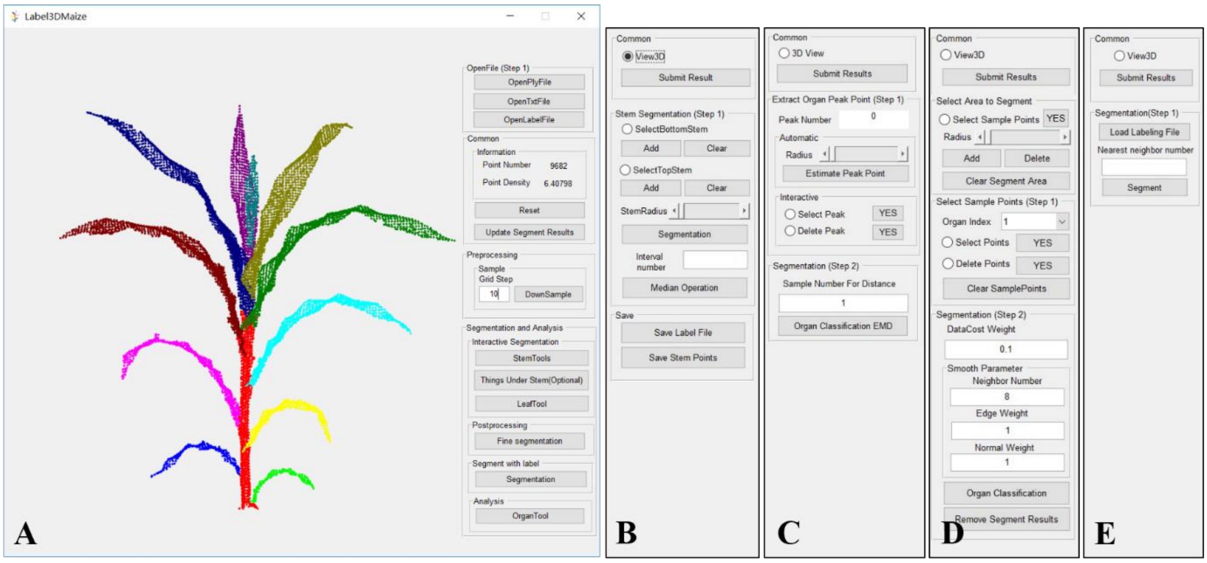

Figure 5: Interfaces of Label3DMaize. (A) The main interface of the toolkit, composed of a visualization window and an embedded dialog. (B)-(E) Dialog of stem segmentation, coarse segmentation, fine segmentation, and sample-based segmentation. The visualization window is not shown in these sub-interfaces.

3.2 Visualization and accuracy evaluation

To evaluate coarse and fine segmentation accuracy, the point clouds of three varieties in four different growth stages of maize shoots are segmented using Label3DMaize. Figure 6 shows the visualization results. According to the visualization results, no significant differences were observed between the coarse and fine segmentation. Yet, fine segmentation improved the segmentation effect of the details, especially near the connection region of organs.

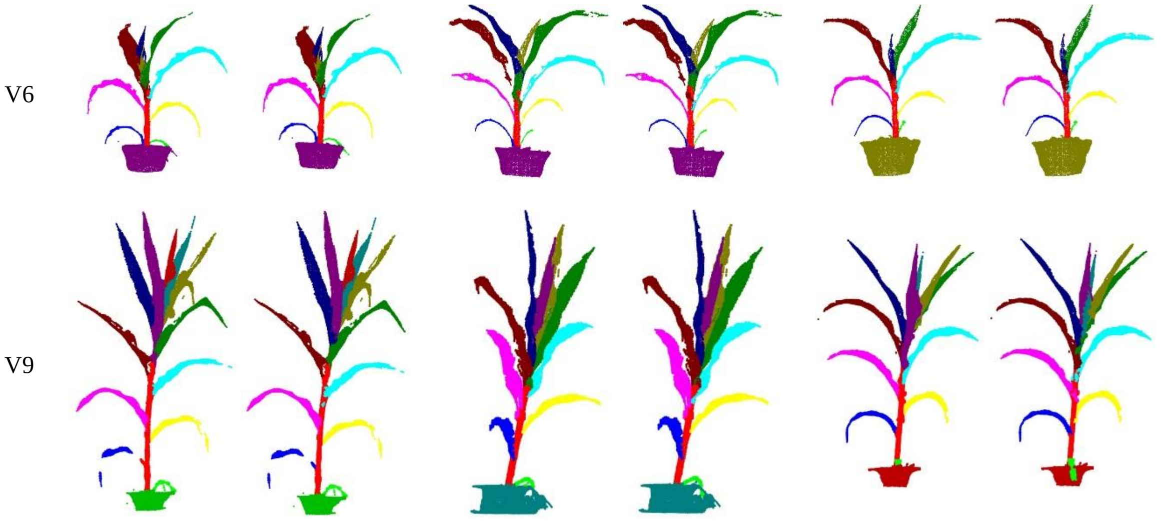

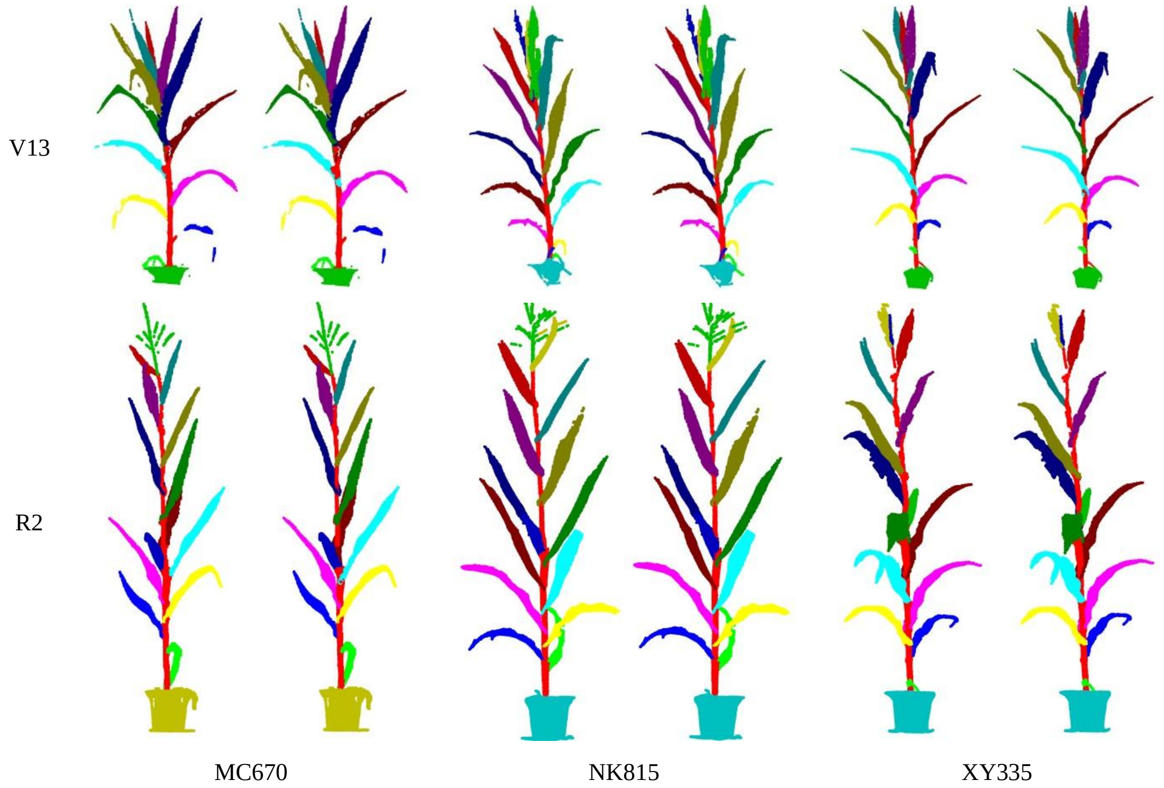

Figure 6 Visualization of maize shoot segmentation results of three cultivars at four growth stages. In each sub-figure, the left and right are coarse and corresponding fine segmentation results, respectively.

This study has further provided numerical accuracy results to quantitatively evaluate the difference between coarse and fine segmentation (Table 1). The precision, recall and F1-score of each organ were estimated based on fine segmentation as the ground truth. The averaged precision and recall of all shoot organs were taken as the precision and recall. Macro-F1 and micro-F1 are calculated using the precision and recall of the shoot and organs averaged value, respectively. It can be seen from Table 1 that although the accuracy of coarse and fine segmentation differed, the overall difference was not significant.

Table 1: Accuracy evaluation of coarse and fine segmentation

	Overall accuracy	Precision	Recall	Micro-F1	Macro-F1
Mean	0.972099	0.967282	0.956173	0.961458	0.955593
Min	0.897683	0.91954	0.841063	0.878553	0.853139
Max	0.993867	0.991753	0.991315	0.991534	0.991175

3.3 Segmentation efficiency

The efficiency of plant point cloud segmentation is an essential indicator for the practicality for training data annotation tools for deep learning. Table 2 shows the time consumed in the different steps for maize shoot segmentation at four growth stages using Label3DMaize on a workstation (Intel Core i7 processor, 3.2GHz CPU, 32GB of memory, Windows 10 operating system), including the interactive manual operations and segmentation computations. It can be seen that point cloud segmentation takes about 4-10 minutes per shoot, in which coarse segmentation takes about 10%-20% of the total time. In the whole segmentation process, the manual interaction time cost is significantly higher than that of automated computation. The segmentation efficiency is positively related to the number of leaves.

This study also analyzed the detailed time costs. (1) The time cost of stem segmentation. In the early growth stages of a maize shoot, the stem is relatively upright, so users only need to select the bottom and

upper points of the stem and specify a suitable radius. However, in the late growth stages, the maize shoot height becomes higher, and the stem becomes thinner from bottom to top. Meanwhile, the upper part curves, so interactive median segmentation is needed, which increases the segmentation time. (2) The time cost of coarse segmentation. The major interactive operation of coarse segmentation is that the user selects or adjusts the highest organ points. As the maize shoot grows, the number of organs gradually increases, so the time costs for the interactive operation of picking points also increases. Meanwhile, the growth of shoot organs significantly increases the occlusion among organs. Thus, the appropriate angles of view for users have to be found to determine the highest organ points, which is time-consuming. (3) The time cost of fine segmentation. An increase in the number of organs causes false segmentation of more organs at the connection regions. Therefore, the fine segmentation of maize shoots with more organs would take more time. Besides, the segmentation efficiency is related to the shoot architecture; the spatial distances between adjacent organs are much larger in flattened shoots than that of relatively compact ones, which increases the segmentation efficiency of flattened shoots.

Table 2: Segmentation time of different steps on maize shoots at four growth stages using Label3DMaize

Growth period	Point number of a maize shoot		Time cost (s)									
	Input	After simplification	$\underline{t_1}$	t_2	$\underline{t_3}$	t_4	$\underline{t_5}$	$\underline{t_6}$	t_7	t_8	$\underline{t_9}$	T
V6	45833	13196	10	0.2	16	4	30.2	120	0.05	0.5	100	**250.75**
V9	62523	13953	10	0.2	21	4	35.2	220	0.05	0.6	100	**355.85**
V13	70873	12102	14	0.2	32	5	51.2	400	0.05	0.6	100	**551.85**
R2	71909	13224	14	0.2	35	5	54.2	400	0.05	0.6	100	**554.85**

* t_1: Time for stem point selection and radius setting. t_2: Time for segmentation computation of stem points. t_3: Time for seed points selection of organ instances. t_4: Time for organ segment computation. t_5: Time for coarse segmentation, where $t_5 = t_1+t_2+t_3+t_4$. t_6: Time for fine segmentation operations. t_7: Time for fine segmentation computation. t_8: Time for sample-based segmentation. t_9: Time for other operations, e.g., the alternation between main and sub-interfaces. T: Total time costs. Underlined and un-underlined identifiers indicates the time cost for manual interactions and automated computation respectively.

4 Discussion

4.1 Shoot-organ point cloud segmentation

Most non-destructive 3D data acquisition of plants focus on individual plant scale. Thus point cloud segmentation from shoot to organ is of significance. Representative shoot-organ point cloud segmentation is realized by region growing combined with adjusting leaf number and stem diameter parameters according to the shoot architecture and stem morphological features [31]. Leaf overlap challenges shoot segmentation, especially for upper leaves in compact shoot architecture. Besides, the robustness of the segmentation algorithm also needs to be verified when processing many point clouds. Once the segmentation is complete, it is difficult to correct the false segmentation points. Although commercial software, such as Geomagic Studio, can solve this problem, it is quite complicated and time-consuming. In contrast, the Label3DMaize toolkit integrates a top-to-down segmentation algorithm and interactive operations according to the morphological structure of maize shoots, which can realize semi-automatic fine point cloud segmentation. The top-to-down coarse segmentation ensures topological accuracy, and the interactive operations improve the segmentation accuracy and details. Although coarse segmentation can meet the basic demand for phenotype extraction, it is not satisfactory for high-precision phenotypic analysis and 3D reconstruction

based on point clouds. In contrast, fine segmentation is more satisfactory for the latter demands. The toolkit can solve the point cloud segmentation problem of compact architecture or organ overlapping shoots. Although skeleton extraction methods [29, 30] also provide an interactive way to improve the segmentation accuracy, they offer skeleton interaction, which hardly improves the segmentation point details.

Since 3D point cloud annotation tools for plants are lacking, researchers segment plants through multi-view image labelling, deep learning-based image segmentation, MVS reconstruction, and a voting strategy [42]. However, these methods cause a lot of organ occlusion from different view angles; thus, it is hard to segment plants with multiple organs through image labelling and MVS reconstruction. Jin et al. [32] transformed point cloud data into a voxel format, constructed a training set containing 3000 maize shoots via data enhancement, and proposed a convolutional neural network (VCNN) to segment stem and leaf point cloud of maize shoots. Label3DMaize enables researchers to directly handle 3D point cloud segmentation and data annotation without transforming point cloud data into the voxel form. Meanwhile, using the acquired data directly improves the diversity of training set data, rather than by data enhancement, and can thus improve the robustness of the learned model. In addition, label3DMaize can separate the tassel and ear except for the stem and leaf, facilitating phenotype extraction of the tassel (such as the number of tassel branches, the compactness of tassel, etc.) and ears (such as the ear height).

4.2 Practicability of Label3DMaize

In our recent works, the MVS-Pheno platform [16] has been used to obtain high-throughput 3D point cloud data of maize shoots at different ecological sites for various genotypes and growth stages. However, the underlying knowledge about genotypes and the differences in cultivation management have not been fully explored, indicating that high-throughput phenotypic acquisition is far from practical application. Therefore, it is urgent to establish automatic and online data analysis approaches [43]. However, due to the complexity of plant morphological structure, it is difficult to realize automatic 3D segmentation from the plant morphological characteristics and regional growth method only. Deep learning is a feasible way to realize automatic segmentation by mining deep features of plant morphology. The greatest challenge in 3D point cloud segmentation by deep learning is the lack of high precision and efficient data annotation tools. Most of the existing 3D data annotation methods are for voxel data [32, 44], not 3D point clouds. Thus, Label3DMaize provides a practical tool for 3D point cloud data annotation for maize and could be a reference for other plants.

Unlike RGB image data annotation [35], data enhancement does not that significantly improve the model robustness of 3D point cloud segmentation models. Thus high-quality data annotation is important. It takes 4-10 minutes to label a maize shoot point cloud by Label3DMaize, and this labeling efficiency can meet the needs of constructing a training dataset for deep learning. The fine segmentation module in Label3DMaize ensures accurate segmentation of detailed features at the organ connections, and is thus satisfactory for organ-level 3D reconstruction. If high precision of the annotation is not required, coarse segmentation results can be used as the annotation data, thus saving a lot of time.

Point clouds with less noise are required when using Label3DMaize, so the toolkit is more suitable for segmenting point clouds derived by MVS reconstruction. For shoots with much random noise obtained by 3D scanners [30], point cloud denoising should be performed first, and then set as input of the toolkit for segmentation. Compared with image annotation, the data annotation efficiency of Label3DMaize is still lower, and fine segmentation requires more manual interaction, which has higher requirements for user experience and concentration. Thus the algorithm for Label3DMaize needs improvement to raise the automation level of point cloud segmentation.

4.3 Future work

At present, a large amount of 3D point cloud data of maize shoots has been obtained using MVS-Pheno. In our future study, representative data will be selected and annotated by Label3DMaize, then a 3D maize shoot annotation dataset will be constructed. A deep learning-based point cloud segmentation model will then be developed to realize the automatic segmentation of maize shoots. Subsequently, online phenotypic extraction and 3D reconstruction of maize shoots algorithms will be studied using the well-segmented point clouds. The segmentation algorithm and this toolkit will be extended to other crops according to their morphological characteristics, which will promote the automatic 3D point cloud segmentation of plants.

Additional files

All the additional files can be found at https://github.com/syau-miao/Label3DMaize.git
Supplementary Program. Executable program of Label3DMaize, which requires that Matlab runtime (Version 9.2 or above) installed.
Supplementary Data S1. The acquired point clouds of maize shoots described in Section "Field experiment and data acquisition", also as the input of the program.
Supplementary Data S2. Coarse segmentation results of the input shoots.
Supplementary Data S3. Fine segmentation results, derived based on the coarse ones.
Supplementary Data S4. Sample based segmentation results, derived from the fine segmentation results.

Acknowledgements

This work was partially supported by Construction of Collaborative Innovation Center of Beijing Academy of Agricultural and Forestry Sciences (KJCX201917), Science and Technology Innovation Special Construction Funded Program of Beijing Academy of Agriculture and Forestry Sciences, the National Natural Science Foundation of China (31871519), Reform and Development Project of Beijing Academy of agricultural and Forestry Sciences, China Agriculture Research System (CARS-02), and the Construction of Scientific Research and Innovation Platform in Beijing Academy of Agriculture and Forestry Sciences (PT2020-24). We would like to thank Tianjun Xu, in Maize Research Center of Beijing Academy of agricultural and Forestry Sciences, for providing experimental materials.

References

1. Bucksch A, Atta-Boateng A, Azihou AF, Battogtokh D, Baumgartner A, Binder BM, et al. Morphological Plant Modeling: Unleashing Geometric and Topological Potential within the Plant Sciences. Front Plant Sci. 2017;8:16. doi:10.3389/fpls.2017.00900.

2. Gibbs JA, Pound M, French AP, Wells DM, Murchie E and Pridmore T. Approaches to three-dimensional reconstruction of plant shoot topology and geometry. Funct Plant Biol. 2017;44 1:62-75. doi:10.1071/FP16167.

3. Lin Y. LiDAR: An important tool for next-generation phenotyping technology of high potential for plant phenomics? Comput Electron Agric. 2015;119:61-73. doi:10.1016/j.compag.2015.10.011.

4. Zhao C, Zhang Y, Du J, Guo X, Wen W, Gu S, et al. Crop Phenomics: Current Status and Perspectives. Front Plant Sci. 2019;10:714. doi:10.3389/fpls.2019.00714.

5. Perez-Sanz F, Navarro PJ and Egea-Cortines M. Plant phenomics: an overview of image acquisition technologies and image data analysis algorithms. GigaScience. 2017;6 11:18. doi:10.1093/gigascience/gix092.

6. Rahman A, Mo C and Cho B-K. 3-D Image Reconstruction Techniques for Plant and Animal Morphological Analysis-A Review. Journal of Biosystems Engineering. 2017;42 4:339-49.

7. Vos J, Evers JB, Buck-Sorlin GH, Andrieu B, Chelle M and de Visser PHB. Functional-structural plant modelling: a new versatile tool in crop science. J Exp Bot. 2010;61 8:2101-15. doi:10.1093/jxb/erp345.

8. Louarn G and Song Y. Two decades of functional–structural plant modelling: now addressing fundamental questions in systems biology and predictive ecology. Ann Bot. 2020;126 4:501-9. doi:10.1093/aob/mcaa143.

9. Ziamtsov I and Navlakha S. Machine Learning Approaches to Improve Three Basic Plant Phenotyping Tasks Using Three-Dimensional Point Clouds. Plant Physiol. 2019;181 4:1425-40. doi:10.1104/pp.19.00524.

10. Rist F, Herzog K, Mack J, Richter R, Steinhage V and Töpfer R. High-Precision Phenotyping of Grape Bunch Architecture Using Fast 3D Sensor and Automation. Sensors. 2018;18 3:763.

doi:10.3390/s18030763.

11. Thapa S, Zhu F, Walia H, Yu H and Ge Y. A Novel LiDAR-Based Instrument for High-Throughput, 3D Measurement of Morphological Traits in Maize and Sorghum. Sensors. 2018;18 4:1187. doi:10.3390/s18041187.

12. Hu Y, Wang L, Xiang L, Wu Q and Jiang H. Automatic Non-Destructive Growth Measurement of Leafy Vegetables Based on Kinect. Sensors. 2018;18 3:806. doi:10.3390/s18030806.

13. Chaivivatrakul S, Tang L, Dailey MN and Nakarmi AD. Automatic morphological trait characterization for corn plants via 3D holographic reconstruction. Comput Electron Agric. 2014;109:109-23. doi:j.compag.2014.09.005.

14. Elnashef B, Filin S and Lati RN. Tensor-based classification and segmentation of three-dimensional point clouds for organ-level plant phenotyping and growth analysis. Comput Electron Agric. 2019;156:51-61. doi:https://doi.org/10.1016/j.compag.2018.10.036.

15. Duan T, Chapman SC, Holland E, Rebetzke GJ, Guo Y and Zheng B. Dynamic quantification of canopy structure to characterize early plant vigour in wheat genotypes. J Exp Bot. 2016;67 15:4523-34. doi:10.1093/jxb/erw227.

16. Wu S, Wen W, Wang Y, Fan J, Wang C, Gou W, et al. MVS-Pheno: A Portable and Low-Cost Phenotyping Platform for Maize Shoots Using Multiview Stereo 3D Reconstruction. Plant Phenomics. 2020;2020:17. doi:10.34133/2020/1848437.

17. Zhang XH, Huang CL, Wu D, Qiao F, Li WQ, Duan LF, et al. High-Throughput Phenotyping and QTL Mapping Reveals the Genetic Architecture of Maize Plant Growth. Plant Physiol. 2017;173

3:1554-64. doi:10.1104/pp.16.01516.

18. Cabrera-Bosquet L, Fournier C, Brichet N, Welcker C, Suard B and Tardieu F. High-throughput estimation of incident light, light interception and radiation-use efficiency of thousands of plants in a phenotyping platform. New Phytol. 2016;212 1:269-81. doi:10.1111/nph.14027.

19. Jin S, Su Y, Song S, Xu K, Hu T, Yang Q, et al. Non-destructive estimation of field maize biomass using terrestrial lidar: an evaluation from plot level to individual leaf level. Plant Methods. 2020;16 1:69. doi:10.1186/s13007-020-00613-5.

20. Zermas D, Morellas V, Mulla D and Papanikolopoulos N. 3D model processing for high throughput phenotype extraction – the case of corn. Comput Electron Agric. 2019:105047. doi:https://doi.org/10.1016/j.compag.2019.105047.

21. Jin SC, Su YJ, Gao S, Wu FF, Hu TY, Liu J, et al. Deep Learning: Individual Maize Segmentation From Terrestrial Lidar Data Using Faster R-CNN and Regional Growth Algorithms. Front Plant Sci. 2018;9:10. doi:10.3389/fpls.2018.00866.

22. Zhan Q, Liang Y and Xiao Y. Color-based segmentation of point clouds. *Laser scanning*. 2009, p. 248-52.

23. Itakura K and Hosoi F. Automatic Leaf Segmentation for Estimating Leaf Area and Leaf Inclination Angle in 3D Plant Images. Sensors. 2018;18 10:3576.

24. Sun SP, Li CY, Chee PW, Paterson AH, Jiang Y, Xu R, et al. Three-dimensional photogrammetric mapping of cotton bolls in situ based on point cloud segmentation and clustering. ISPRS-J Photogramm Remote Sens. 2020;160:195-207.

doi:10.1016/j.isprsjprs.2019.12.011.

25. Li D, Shi G, Kong W, Wang S and Chen Y. A Leaf Segmentation and Phenotypic Feature Extraction Framework for Multiview Stereo Plant Point Clouds. IEEE Journal of Selected Topics in Applied Earth Observations and Remote Sensing. 2020;13:2321-36. doi:10.1109/JSTARS.2020.2989918.

26. Paulus S, Dupuis J, Mahlein AK and Kuhlmann H. Surface feature based classification of plant organs from 3D laserscanned point clouds for plant phenotyping. BMC Bioinformatics. 2013;14:12. doi:http://dx.doi.org/10.1186/1471-2105-14-238.

27. Wahabzada M, Paulus S, Kersting K and Mahlein AK. Automated interpretation of 3D laserscanned point clouds for plant organ segmentation. BMC Bioinformatics. 2015;16:11. doi:10.1186/s12859-015-0665-2.

28. Li YY, Fan XC, Mitra NJ, Chamovitz D, Cohen-Or D and Chen BQ. Analyzing Growing Plants from 4D Point Cloud Data. ACM Trans Graph. 2013;32 6:10. doi:10.1145/2508363.2508368.

29. Xiang LR, Bao Y, Tang L, Ortiz D and Salas-Fernandez MG. Automated morphological traits extraction for sorghum plants via 3D point cloud data analysis. Comput Electron Agric. 2019;162:951-61. doi:10.1016/j.compag.2019.05.043.

30. Wu S, Wen W, Xiao B, Guo X, Du J, Wang C, et al. An Accurate Skeleton Extraction Approach From 3D Point Clouds of Maize Plants. Front Plant Sci. 2019;10:248. doi:10.3389/fpls.2019.00248.

31. Jin SC, Su YJ, Wu FF, Pang SX, Gao S, Hu TY, et al. Stem-Leaf Segmentation and Phenotypic

Trait Extraction of Individual Maize Using Terrestrial LiDAR Data. IEEE Trans Geosci Remote Sensing. 2019;57 3:1336-46. doi:10.1109/tgrs.2018.2866056.

32. Jin S, Su Y, Gao S, Wu F, Xu K, Ma Q, et al. Separating the Structural Components of Maize for Field Phenotyping Using Terrestrial LiDAR Data and Deep Convolutional Neural Networks. IEEE Trans Geosci Remote Sensing. 2019;58 4:2644 - 58. doi:10.1109/TGRS.2019.2953092.

33. Griffiths D and Boehm J. A Review on Deep Learning Techniques for 3D Sensed Data Classification. Remote Sensing. 2019;11 12:29. doi:10.3390/rs11121499.

34. Bernotas G, Scorza LCT, Hansen MF, Hales IJ, Halliday KJ, Smith LN, et al. A photometric stereo-based 3D imaging system using computer vision and deep learning for tracking plant growth. GigaScience. 2019;8 5:15. doi:10.1093/gigascience/giz056.

35. Russell BC, Torralba A, Murphy KP and Freeman WT. LabelMe: A database and web-based tool for image annotation. Int J Comput Vis. 2008;77 1-3:157-73. doi:10.1007/s11263-007-0090-8.

36. Dutagaci H, Rasti P, Galopin G and Rousseau D. ROSE-X: an annotated data set for evaluation of 3D plant organ segmentation methods. Plant Methods. 2020;16 1:14. doi:10.1186/s13007-020-00573-w.

37. Gené-Mola J, Sanz-Cortiella R, Rosell-Polo JR, Morros JR, Ruiz-Hidalgo J, Vilaplana V, et al. Fuji-SfM dataset: A collection of annotated images and point clouds for Fuji apple detection and location using structure-from-motion photogrammetry. Data in Brief. 2020;30:105591.

38. Abendroth LJ, Elmore RW, Matthew J. Boyer and Marlay SK. *Corn Growth and Development*.

Report no. PMR 1009, 2011. Ames, Iowa: Iowa State University Extension.

39. Cuturi M. Sinkhorn Distances: Lightspeed Computation of Optimal Transportation Distances. Advances in Neural Information Processing Systems. 2013;26:2292-300.

40. Richard S. Diagonal equivalence to matrices with prescribed row and column sums. The American Mathematical Monthly. 1967;74 4:402-5.

41. Boykov Y and Kolmogorov V. An experimental comparison of min-cut/max-flow algorithms for energy minimization in vision. IEEE Trans Pattern Anal Mach Intell. 2004;26 9:1124-37. doi:10.1109/tpami.2004.60.

42. Shi WN, van de Zedde R, Jiang HY and Kootstra G. Plant-part segmentation using deep learning and multi-view vision. Biosys Eng. 2019;187:81-95. doi:10.1016/j.biosystemseng.2019.08.014.

43. Artzet S, Chen T-W, Chopard J, Brichet N, Mielewczik M, Cohen-Boulakia S, et al. Phenomenal: An automatic open source library for 3D shoot architecture reconstruction and analysis for image-based plant phenotyping. bioRxiv. 2019:805739. doi:10.1101/805739.

44. Liu Z, Tang H, Lin Y and Han S. Point-Voxel CNN for Efficient 3D Deep Learning. *33rd Conference on Neural Information Processing Systems*. Vancouver, Canada: Curran Associates, Inc., 2019, p. 965-75.

Click here to access/download
Supplementary Material
Supplementary Material.txt

Personal Cover

Click here to access/download;Personal Cover;Cover letter.docx

Dear Editors,

We would like to submit the enclosed manuscript entitled "Label3DMaize: toolkit for 3D point cloud data annotation of maize shoots " to the Collection of "Plant Phenomics: Data Integration and Analyses ".

The work described has not been submitted elsewhere for publication, in whole or in part, and all the authors listed have approved the manuscript that is enclosed. We believe that the contents of this manuscript will interest the general readers of your journal. This paper reports the development of a 3D point cloud annotation toolkit, Label3DMaize, which integrates a proposed point cloud segmentation algorithm and interactive operations. The toolkit aims to provide practical means to solve numerous challenges of point cloud data annotation and construct training datasets to study 3D deep leaning models for plants. To our knowledge, this is the first 3D point cloud annotation tool for plants. Label3DMaize is a practical 3D point cloud tool for maize data annotation and is a reference for other plants. It provides technical support to improve the automation level of plant point cloud segmentation, even for high-throughput 3D plant phenotyping.

Thank you very much for your time and consideration.

Sincerely yours,

Xinyu Guo
