# Supplementary material for: Label3DMaize: toolkit for 3D point cloud data annotation of maize shoots
Source: Gigascience. 2021 May 7;10(5):giab031. doi: 10.1093/gigascience/giab031 (PMC8105162; doi:10.1093/gigascience/giab031)
Supplement: giab031_GIGA-D-20-00335_Revision_1 [file giab031_giga-d-20-00335_revision_1.pdf]

GigaScience

Label3DMaize: toolkit for 3D point cloud data annotation of maize shoots

--Manuscript Draft--

**Manuscript Number:**	GIGA-D-20-00335R1
**Full Title:**	Label3DMaize: toolkit for 3D point cloud data annotation of maize shoots
**Article Type:**	Research

Funding Information:

Construction of Collaborative Innovation Center of Beijing Academy of Agricultural and Forestry Sciences (KJCX201917)	prof. Xinyu Guo
Science and Technology Innovation Special Construction Funded Program of Beijing Academy of Agriculture and Forestry Sciences (KJCX20210413)	Dr. Weiliang Wen
National Natural Science Foundation of China (31871519)	prof. Xinyu Guo
National Natural Science Foundation of China (32071891)	Dr. Weiliang Wen
Reform and Development Project of Beijing Academy of agricultural and Forestry Sciences	Not applicable
China Agriculture Research System (CARS-02)	Not applicable

Abstract:

Background

Three-dimensional (3D) point cloud is the most direct and effective data form for studying plant structure and morphology. In point cloud studies, the point cloud segmentation of individual plants to organs directly determines the accuracy of organ-level phenotype estimation and the 3D plant reconstruction reliability. However, highly accurate, automatic, and robust point cloud segmentation approaches for plants are unavailable. Thus, the high-throughput segmentation of many shoots is challenging. Although deep learning can feasibly solve this issue, software tools for 3D point cloud annotation to construct the training dataset are lacking.

Results

In this paper, a top-to-down point cloud segmentation algorithm using optimal transportation distance for maize shoots is proposed. On this basis, a point cloud annotation toolkit, Label3DMaize, for maize shoot is developed. Further, the toolkit was applied to achieve semi-automatic point cloud segmentation and annotation of maize shoots at different growth stages, through a series of operations, including stem segmentation, coarse segmentation, fine segmentation, and sample-based segmentation. The toolkit takes about 4 to 10 minutes to segment a maize shoot, and consumes 10%-20% of the total time if only coarse segmentation is required. Fine segmentation is more detailed than coarse segmentation, especially at the organ connection regions. The accuracy of coarse segmentation can reach 97.2% of the fine segmentation.

Conclusion

Label3DMaize integrates point cloud segmentation algorithms and manual interactive operations, realizing semi-automatic point cloud segmentation of maize shoots at different growth stages. The toolkit provides a practical data annotation tool for further online segmentation research based on deep learning and is expected to promote automatic point cloud processing of various plants.

Corresponding Author: Xinyu Guo, Ph.D.
Beijing Research Center for Information Technology in Agriculture: National

Powered by Editorial Manager® and ProduXion Manager® from Aries Systems Corporation

	Engineering Research Center for Information Technology in Agriculture<br>Beijing, CHINA
**Corresponding Author Secondary Information:**	
**Corresponding Author's Institution:**	Beijing Research Center for Information Technology in Agriculture: National Engineering Research Center for Information Technology in Agriculture
**Corresponding Author's Secondary Institution:**	
**First Author:**	Teng Miao
**First Author Secondary Information:**	
**Order of Authors:**	Teng Miao
	Weiliang Wen
	Sheng Wu
	Chao Zhu
	Yinglun Li
	Xinyu Guo, Ph.D.
**Order of Authors Secondary Information:**	
**Response to Reviewers:**	Response to Editor<br>Your manuscript "Label3DMaize: toolkit for 3D point cloud data annotation of maize shoots" (GIGA-D-20-00335) has been assessed by our reviewers. Based on these reports, and my own assessment as Editor, I am pleased to inform you that it is potentially acceptable for publication in GigaScience, once you have carried out some essential revisions suggested by our reviewers.<br>** We very much appreciate your careful reading of our manuscript and valuable suggestions. We have carefully considered the comments and have revised the manuscript accordingly.<br>Some descriptions of the steps involved are not clear and need better clarifications and details.<br>** We have simplified the seed point determination section (section 2.5.1), and supplemented pseudocode of optimal transportation matrix in section 2.5.2.<br>Comparison with at least 1-2 state of the art methods is also required. Another concern is the dependency of Label3DMaize on data generated through MVS-Pheno - which is commercial software - as we are an open science journal, work must be reproducible and all open and available for reuse without the need to access proprietary software. We'd need you to clarify this further and also provide tests to prove that it is not just dependent on MVS-Pheno.<br>** We conducted comparison of coarse segmentation with region growing and PointNet-based model (Section 3.4).<br>Label3DMaize does not depends on data generated through MVS-Pheno. Any point cloud data of maize shoot could be the input of this toolkit, such as data acquired using any 3D scanner, or point clouds reconstructed through manually captured multi-view images. Data used in the new supplemented section 3.4 were point cloud acquired using 3D scanner. Tomato point cloud data used in section 3.5 was downloaded from a literature. This have been clarified at the end of the first paragraph in section 4.2.<br>“Specifically, Label3DMaize does not depend on data generated through MVS-Pheno. Any point cloud of maize shoot can be the toolkit input, including data acquired using 3D scanners (Figure 7), or reconstructed from multi-view images acquired by handheld cameras.”<br>In addition, please register any new software application in the bio.tools and SciCrunch.org databases to receive RRID (Research Resource Identification Initiative ID) and biotoolsID identifiers, and include these in your manuscript. This will facilitate tracking, reproducibility and re-use of your tool.<br>** the biotoolsID is label3dmaize, which have been supplemented in the last paragraph in Introduction.<br>** We have uploaded all new data files and updated the latest version of Label3DMaize in GitHub.

Powered by Editorial Manager® and ProduXion Manager® from Aries Systems Corporation

Response to Reviewer #1:
This very interesting Research Article describes Label3DMaize, which is a toolkit that utilises a top-to-down segmentation algorithm to deliver fine point cloud segmentation of maize shoots. Multi-view images of maize cultivars were captured using the MVS-Pheno platform, which has been described previously (Wu et al., 2020, doi:10.34133/2020/1848437). The top-to-down coarse segmentation ensures topological accuracy. Additional morphological operations then improve the segmentation of maize plants, with the resultant 3D points being structured on connectivity and morphological features. A detailed workflow is described in the article. Label3DMaize is publicly available on GitHub and has been ascribed an OSI-approved GPL-3.0 License. The software is easy to run, simply requiring MATLAB Runtime 9.2. Label3DMaize is straightforward and easy-to-use, and the interface enables users to rotate, zoom, translate, select points, and improve the segmentation. A pop-up window allows users to select points and to generate segmentations. Test data of individual maize plants are available from the GitHub archive.
This is a very easy-to-use toolkit and I recommend this paper for publication in GigaScience.
** We very much appreciate your careful reading of our manuscript and comments.

Response to Reviewer #2:
The authors give a practical tool for maize organ segmentation by an interactive way. The ideas and methods presented in this paper are straightforward and make sense for me. The Figs. and language are also sufficiently good and the obtained segmented results of the maize shoots show a clear, practical applicability as well. I recommend the paper to be accepted after minor revision. Below are some detail suggestion:
** We very much appreciate your careful reading of our manuscript and valuable suggestions. We have carefully considered the comments and have revised the manuscript accordingly.
1) The authors need to provide the contributions of the work at the end of the introduction to enhance the innovations of the paper.
** A brief contribution of the work have been provided at the end of the introduction. “The toolkit integrates clustering approaches and computer interactions supported through maize structural knowledge. Optimal transportation based coarse segmentation is satisfactory for basic segmentation tasks, and fine segmentation offers users way to calibrate the segmentation details. This plant-oriented tool could be used to segment point cloud data of various maize growth periods, and provide practical data labeling tool for segmentation research based on deep learning.”
2) In the workflow of the segmentation, the initial segmentation is the key step, however, the descriptions of this part (pages 9 and 10) are not clear. I suggest the authors rephrase this part. It is enough to introduce the general ideas of the algorithm based on the transportation distances. The details of the algorithm can be put into the pseudocode.
** Section 2.5.1 has been rephrased and the pseudocode has been supplemented in section 2.5.2.
3) In some cases, the multiple maize shoots are hardly to separate. Does the Label3DMaize have the capability to segment multiple instances simultaneously? I mean whether the tool support multiple maize shoots as inputs? If you confront this situation, how do you process?
** Label3DMaize could not segment point cloud containing multiple instances. For this situation, the point cloud have to be preprocessed into separated individual shoots. Corresponding descriptions have been supplemented at the beginning of the last paragraph in section 4.2. “Label3DMaize is designed for individual shoots and does not support segmentation of multiple maize shoots. Thus, point clouds containing multiple shoots have to be preprocessed into individual shoot point clouds first, through spatial connection property of points, or interactively separated using commercial software (such as CloudCompare, Geomagic Studio, etc.). This shoot separation preprocess is easy for scenarios without cross organs. Thus, point cloud data acquisition is important

Powered by Editorial Manager® and ProduXion Manager® from Aries Systems Corporation

for subsequent segmentation."
4) The authors mentioned that point clouds scanned by 3d scanners have much random noise than MVS point cloud. To the best of our knowledge, I think the maize shoot point clouds derived by the TLS or other similar scanners should have higher accuracy, precision, and less noise than MVS point clouds. The related sentences need to be reconsidered.
** Indeed, 3D scanners have higher accuracy and precision. In our practice, point clouds obtained using 3D scanner have many random noise for maize shoots, especially near the boundary of leaves (We use FARO X130 scanner). Point cloud reconstructed using mutli-view images through commercial software (such as PhotoScan) have less noise. However, I think this depends the 3D scanner or MVS reconstruction software. We modified the related description from "For shoots with much random noise obtained by 3D scanners, point cloud denoising should be performed first" into "For shoots with much random noise, point cloud denoising should be performed first".
5) In the experiments, the authors should conduct comparisons with at least one or two state-of-the-art methods.
** A new section 3.4 was supplemented in our manuscript. We conducted comparison of coarse segmentation with region growing and PointNet-based model.

Response to Reviewer #3:
Point cloud is the most important representation for shape information acquisition, and that is a useful form of shape information processing. Maize is one of the most important crops. Each maize has its own special shape, but it follow a strong rule of structures so that learning based tool could be developed and be useful.
High-precision segmentation tool is an important basis for mathematical modeling of crop growth, for crop measurement and for crop yield estimation. Deep learning method becomes effective for the classification and segmentation of point clouds, but it is difficult to deal directly with the problem of maize object segmentation. Therefore, the topic of the paper has important research value and technical challenges.
Based on point cloud shape analysis, a point cloud interactive segmentation method and a point cloud interactive segmentation labeling tool are developed. The tool Label3DMaize is based on the basic knowledge of maize morphology, and that is effective for 3D point cloud data annotation/labeling of maize shoots. Experimental results show that segmentations of maize shoot are effective.
The contribution of this paper is in the use of the optimal transmission and point cloud clustering methods. Human computer interaction is included with maize morphological structure knowledge supported. This developed plant-oriented point cloud segmentation annotation/labeling tool could be used to point data of different reproductive period of the maize, and could be used to deep learning-based point cloud segmentation annotation of other crops also.
** We very much appreciate your careful reading of our manuscript and valuable suggestions. We have carefully considered the comments and have revised the manuscript accordingly.
Disadvantages and modification recommendations:
1. Since MRF method is used for precise segmentation of point cloud, complex interactive operations are needed. Segmentation results of the approach are not intuitive, it is recommended to introduce a more direct way to assist the MRF segmentation.
** This is a very practical suggestion for us to improve this toolkit. We added a new function to assist the MRF segmentation and the description was supplemented at the end of section 2.6. "In addition, users cloud assign organ label to the region of interest points after the above mentioned step 2, which offers a more direct way for fine segmentation.". As a result, this improvement directly reduced the segmentation time, see Table 3.
2. The annotation tool described in the article is mainly designed for the maize, authors should mention whether or not it could be properly extended to other specific crops. It is suggested to specify this extendibility of segmentation and related interactions, hoping to provide better reference for other crop researchers.
** We supplemented section 3.5 to demonstrate the extendibility of the toolkit on other plants, including tomato, cucumber, and wheat shoot.

Powered by Editorial Manager® and ProduXion Manager® from Aries Systems Corporation

3. It is suggested to further strengthen the use of maize morphology knowledge, and to promote the use of knowledge and data joint driven for segmentation and annotation.
** Thanks for this suggestion. The current version have integrated the structural knowledge of maize shoot for segmentation. In our further study, we will integrate the morphology knowledge through deep learning models.
4. It is suggested to further consider using the existing three-dimensional shape model of maize to guide the annotation/ labeling of new maize data.
** Thanks for this suggestion. Corresponding modifications have been made in section 4.3. "What's more, well segmented maize organ data could be used to build a 3D shape model of maize. All the above technologies or data will conversely simplify the segmentation and labeling processes of the toolkit."
5. Typos should be carefully checked
1) Line 55: Therefor === > Therefore
2) Line 127: update === > updates
3) Line 197: distance === > distances
4) Line 361: indicates === > indicate
5) Labelling (Line 383) or labeling (Line 68 and 409)? These should be consistent.
** Thanks for your careful reading and corresponding modifications have been made in the manuscript.
6. More very new papers on point cloud segmentation in the field computer vision and computer graphics should be cited, just like the following:
[1] F. Engelmann, M. Bokeloh, A. Fathi, B. Leibe and M. Niessner, "3D-MPA: Multi-Proposal Aggregation for 3D Semantic Instance Segmentation," 2020 IEEE/CVF Conference on Computer Vision and Pattern Recognition (CVPR), Seattle, WA, USA, pp. 9028-9037, 2020.
[2] Tao Ku, Remco C. Veltkamp, etc, SHREC 2020: 3D point cloud semantic segmentation for street scenes, Computers & Graphics, Volume 93, Pages 13-24, 2020.
** We have cited five related papers on point cloud segmentation in the field computer vision and computer graphics:
39.Engelmann F, Bokeloh M, Fathi A, Leibe B and Nießner M. 3D-MPA: Multi-Proposal Aggregation for 3D Semantic Instance Segmentation. In: 2020 IEEE/CVF Conference on Computer Vision and Pattern Recognition (CVPR) 13-19 June 2020 2020, pp.9028-37.
41.Chang AX, Funkhouser T, Guibas L, Hanrahan P, Huang Q, Li Z, et al. ShapeNet: An Information-Rich 3D Model Repository. Computer ence. 2015.
42.Hackel T, Savinov N, Ladicky L, Wegner JD, Schindler K and Pollefeys M. Semantic3D.net: A new Large-scale Point Cloud Classification Benchmark. 2017.
43.Behley J, Garbade M, Milioto A, Quenzel J, Behnke S, Stachniss C, et al. SemanticKITTI: A Dataset for Semantic Scene Understanding of LiDAR Sequences. In: 2019 IEEE/CVF International Conference on Computer Vision (ICCV) 27 Oct.-2 Nov. 2019 2019, pp.9296-306.
44.Ku T, Veltkamp RC, Boom B, Duque-Arias D, Velasco-Forero S, Deschaud JE, et al. SHREC 2020: 3D point cloud semantic segmentation for street scenes. Comput Graph-UK. 2020;93:13-24. doi:10.1016/j.cag.2020.09.006.

Response to Reviewer #4:
The authors describe a software called Label3DMaize to segment and annotate point cloud data. The software offers different types of segmentation methods, some of them faster than others. In terms of processing speed, Label3DMaize is faster than other platforms. The subject is of clear importance since highly precise phenotypes can be computed from 3D representations. Moreover, 3D representation allows the measurement of hard-to-measure or complex phenotypes.
** We very much appreciate your careful reading of our manuscript and valuable suggestions. We have carefully considered the comments and have revised the manuscript accordingly.
Some comments:
1) This software runs in Matlab, which is vaguely mentioned in the Additional files section. I consider it is important to mention this in the main text because this will impact the accessibility of the software for many potential users.
** Indeed, this should be mentioned in the main text. We supplemented this at the

	beginning of section 3.1: “The Label3DMaize toolkit was developed using Matlab”.<br>2) The 3D point cloud data used in this study was generated by a system (MVS-Pheno) that the same authors developed just recently (the paper was published early 2020). MVS-Pheno is a commercial software it seems. My main concern is the dependency of Label3DMaize on data generated through MVS-Pheno. Is this going to work on data generated on a different system? If so, additional tests showing that must be included in the present study.<br>** MVS-Pheno facilitates high-throughput data acquisition and data management of individual plants. However, Label3DMaize does not depends on this platform. Any point cloud of maize shoot can be the input of the toolkit, including data acquired using 3D scanners, or reconstructed point cloud from multi-view images acquired by handheld cameras. To demonstrate this, data used for comparison with other methods in section 3.4 were acquired using a 3D scanner. And we supplemented this explanation at the end of the first paragraph in section 4.2: “Specifically, Label3DMaize does not depend on data generated through MVS-Pheno. Any point cloud of maize shoot can be the toolkit input, including data acquired using 3D scanners (Figure 7), or reconstructed from multi-view images acquired by handheld cameras.”<br>3) Both the introduction and the discussion sections are a little heavy; there is a constant reuse of ideas, which becomes repetitive and tedious to read. I suggest reducing the text considerably.<br>** Indeed, there is a constant reuse of ideas. We simplified some of the contents. See the beginning of section 4.1.<br>4) Lines 48-49. The authors say that multiple MVS platforms have been developed, but they just mention one reference (reference 16).<br>** Three references about other MVS platforms have been supplemented:<br>19.Nguyen CV, Fripp J, Lovell DR, Furbank R, Kuffner P, Daily H, et al. 3D Scanning System for Automatic High-Resolution Plant Phenotyping. 2016 International Conference on Digital Image Computing: Techniques and Applications (DICTA). 2016, p. 1-8.<br>20.Cao W, Zhou J, Yuan Y, Ye H, Nguyen HT, Chen J, et al. Quantifying Variation in Soybean Due to Flood Using a Low-Cost 3D Imaging System. Sensors. 2019;19 12:2682. doi:10.3390/s19122682.<br>21.Bernotas G, Scorza LCT, Hansen MF, Hales IJ, Halliday KJ, Smith LN, et al. A photometric stereo-based 3D imaging system using computer vision and deep learning for tracking plant growth. GigaScience. 2019;8 5:15. doi:10.1093/gigascience/giz056.<br>5) Examples of previous work regarding 3D point cloud data are presented in the Introduction section, which provides the reader a good context. However, it seems that the examples are presented one after another without a proper link.<br>** These data acquisition technologies are well-known for researchers in this area. Simple listed these key technologies and citing representative references is enough. Detailed explanation about their proper link will make the introduction more heavy. We supplement a review article that describe the link of these technologies.<br>10.Jin SC, Sun XL, Wu FF, Su YJ, Li YM, Song SL, et al. Lidar sheds new light on plant phenomics for plant breeding and management: Recent advances and future prospects. ISPRS-J Photogramm Remote Sens. 2021;171:202-23. doi:10.1016/j.isprsjprs.2020.11.006.<br>6) Clearly, this is a relevant tool that could benefit researchers studying plant morphology as well as breeders. However, I don't think this study fits well within the scope and requirements of GigaScience (e.g. lack of novelty, more tests are required, why not using this in a different species with similar plant structure?, high dependency in other tools, etc…).<br>** We have supplemented application on tomato, cucumber, and wheat. And we have clarified the toolkit did not depends on other tools.<br>7) Section 4.3 could be integrated within other Discussion subsections.<br>** This subsection (section 4.3) describes the work we might conduct in the future, which is different from the other parts of the discussion. Thus, in our opinion, this is clearer than merging into other subsections.
**Additional Information:**	
**Question**	**Response**
Are you submitting this manuscript to a special series or article collection?	No

Powered by Editorial Manager® and ProduXion Manager® from Aries Systems Corporation

**Experimental design and statistics**<br><br>Full details of the experimental design and statistical methods used should be given in the Methods section, as detailed in our Minimum Standards Reporting Checklist. Information essential to interpreting the data presented should be made available in the figure legends.<br><br>Have you included all the information requested in your manuscript?	Yes
**Resources**<br><br>A description of all resources used, including antibodies, cell lines, animals and software tools, with enough information to allow them to be uniquely identified, should be included in the Methods section. Authors are strongly encouraged to cite Research Resource Identifiers (RRIDs) for antibodies, model organisms and tools, where possible.<br><br>Have you included the information requested as detailed in our Minimum Standards Reporting Checklist?	Yes
**Availability of data and materials**<br><br>All datasets and code on which the conclusions of the paper rely must be either included in your submission or deposited in publicly available repositories (where available and ethically appropriate), referencing such data using a unique identifier in the references and in the "Availability of Data and Materials" section of your manuscript.<br><br>Have you have met the above requirement as detailed in our Minimum Standards Reporting Checklist?	Yes

Manuscript-R1

Click here to access/download;Manuscript;Label3DMaize-R1.docx

Label3DMaize: toolkit for 3D point cloud data annotation of maize shoots

Teng Miao[1, †], Weiliang Wen[2, 3, 4, †], Sheng Wu[2, 3, 4], Chao Zhu[1], Yinglun Li[3, 4], Xinyu Guo[2, 3, 4, *]

[1] *College of Information and Electrical Engineering, Shenyang Agricultural University, Shenyang 110161, China.*

[2] *Beijing Research Center for Information Technology in Agriculture, Beijing 100097, China*.

[3] *National Engineering Research Center for Information Technology in Agriculture, Beijing 100097, China*.

[4] *Beijing Key Lab of Digital Plant, Beijing 100097, China*.

* For correspondence. Email: guoxy73@163.com (Xinyu Guo)

† Co-first authors.

Abstract

Background: Three-dimensional (3D) point cloud is the most direct and effective data form for studying plant structure and morphology. In point cloud studies, the point cloud segmentation of individual plants to organs directly determines the accuracy of organ-level phenotype estimation and the 3D plant reconstruction reliability. However, highly accurate, automatic, and robust point cloud segmentation approaches for plants are unavailable. Thus, the high-throughput segmentation of many shoots is challenging. Although deep learning can feasibly solve this issue, software tools for 3D point cloud annotation to construct the training dataset are lacking. **Results**: In this paper, a top-to-down point cloud segmentation algorithm using optimal transportation distance for maize shoots is proposed. On this basis, a point cloud annotation toolkit, Label3DMaize, for maize shoot is developed. Further, the toolkit was applied to achieve semi-automatic point cloud segmentation and annotation of maize shoots at different growth stages, through a series of operations, including stem segmentation, coarse segmentation, fine segmentation, and sample-based segmentation. The toolkit takes about 4 to 10 minutes to segment a maize shoot, and consumes 10%-20% of the total time if only coarse segmentation is required. Fine segmentation is more detailed than coarse segmentation, especially at the organ connection regions. The accuracy of coarse segmentation can reach 97.2% of the fine segmentation. **Conclusion**: Label3DMaize integrates point cloud segmentation algorithms and manual interactive operations, realizing semi-automatic point cloud segmentation of maize shoots at different growth stages. The toolkit provides a practical data annotation tool for further online segmentation researches based on deep learning and is expected to promote automatic point cloud processing of various plants.

Key-words: Label3DMaize, three-dimensional point cloud, segmentation, maize shoot, data annotation.

1 Introduction

The plant structure and morphology are important features for expressing growth and development. At present many research studies underpin the significance of integrating the three-dimensional (3D) morphological characteristics of plants when conducting genetic mapping, adaptability evaluation, and crop yield analysis [1, 2]. Using the 3D data acquisition technology to obtain a 3D point cloud is the most effective way to perceive the plant structure and morphology digitally. However, 3D point clouds are initially obtained in an unordered, unstructured manner and with little semantic information. Therefore, it is critical to use computer graphics technologies and plant morphology knowledge to convert the unstructured 3D point clouds into well-organized and structured data that contains rich morphological features with semantic information. Therefore, plant morphology research based on measured point clouds forms a critical

component of 3D plant phenomics [3-6], 3D plant reconstruction [2, 7], and functional-structural plant models (FSPMs) [8, 9].

The development of 3D data acquisition technology [10] has significantly enriched approaches for fine-scale 3D data acquisition of individual plants, including 3D scanning [11, 12], LiDAR [13], depth camera [14], time of flight (ToF) reconstruction [15], and multi-view stereo (MVS) reconstruction [16, 17]. Owing to the low cost of sensors and better quality of reconstructed point clouds, MVS reconstruction have been widely adopted in many applications. Recently, multi-view image acquisition platforms that can realize semi-automatic and high-throughput 3D data acquisition for individual plants have been developed [18-21] and enable 3D data acquisition for the phenotypic analysis of large-scale breeding materials [22, 23]. However, how to efficiently and automatically process the acquired big data of 3D point clouds is a bottleneck in 3D plant phenotyping.

The key technologies for 3D point cloud data processing include data registration, the region of interest extraction, denoising, segmentation, feature extraction, and mesh generation. Among these tasks, point cloud segmentation is challenging. Therefore, automatic and accurate point cloud segmentation could significantly impact subsequent results of phenotype extraction and 3D reconstruction. Point cloud segmentation can be classified as population-shoot or shoot-organ segmentation. Population-shoot segmentation allows for automatic segmentation of maize population under low density [24] or at early growth stages [25, 26] with little overlap, which can be realized via the spatial distance between shoots. However, it is difficult to achieve automatic segmentation of high density populations or with many overlapping organs in late growth stages. Comparatively, more attention has been paid to shoot-organ segmentation. Though high-quality input point clouds and restricted connections between organs are required, color-based [27] and point clustering [28-30] approaches have also been widely used. For instance, Elnashef et al. [16] used the local geometric features of the organs to segment maize leaves and stems at six-leaf stage. Paulus et al. [31, 32] segmented the grape shoot organs by integrating fast point feature histograms (FPFH), support vector machine (SVM), and region growing approaches. However, these methods can only segment plant shoots with clear connection characteristics between stems and leaves [11] and can hardly solve leaf wrapping stem segmentation problems. For time-series 3D point clouds, the leaf multi-labeling segmentation method was used for organ segmentation and plant growth monitoring [33]. While plant organs could also be segmented through skeleton extraction and hierarchical clustering [34, 35], these methods need interactive manual correction for complex plants to guarantee the segmentation accuracy. Jin et al. [36] proposed a median normalized vector growth algorithm that can segment the stems and leaves of maize shoots. On this basis, an annotation dataset of maize shoots was constructed, and the deep learning method was introduced to improve the automatic segmentation level [37]. However, few parameter interactions are still needed for different shoot architecture and cannot meet the needs of high realistic 3D reconstruction.

Due to the complexity of plant morphology and structure, almost all 3D point cloud segmentation methods for plants need certain manual interaction, which is inconvenient for huge amounts of point cloud data processing, and substantially decreases the efficiency. Therefore, it is necessary to improve the automation of segmentation and increase the throughput of 3D point cloud data processing for plants. Deep learning approaches can effectively solve this problem [21, 38, 39], among which the construction of high-quality training data set is a prerequisite. For example, LabelMe [40] can realize high-quality data annotation for image segmentation. However, 3D point cloud tools for data annotation are rare, especially for plants. Besides, current datasets used for point cloud segmentation are oriented to general segmentation tasks [41-44]. tThe existing datasets for 3D plant segmentation contain only little data [21, 45, 46], which cannot meet the data requirements for high-quality deep learning models.

Since point cloud annotation of plants is labor-intensive and time-consuming, deep learning approaches can be applied to segment plant point clouds. Hence, how to improve the efficiency of high-quality data annotation and develop supporting software tools is the key to automatic point cloud segmentation of plants by deep learning. To meet this data annotation demand, this study used maize as an example and proposes a top-to-down point cloud segmentation algorithm. Besides, a toolkit Label3DMaize (biotoolsID: label3dmaize) for point cloud annotation of maize shoots is developed, which could provide technical support for automatic and high-throughput processing of plant point clouds. The toolkit integrates clustering approaches and computer interactions supported through maize structural knowledge. Optimal transportation based coarse segmentation is satisfactory for basic segmentation tasks, and fine segmentation offers users way to calibrate the segmentation details. This plant-oriented tool could be used to segment point cloud data of various maize growth periods, and provide practical data labeling tool for segmentation research based on deep learning.

2 Materials and Methods

2.1 Field experiment and data acquisition

Three maize cultivars, including MC670, Xianyu 335 (XY335), and NK815, were planted on May 20th, 2019, at the Tongzhou experimental field of Beijing Academy of Agriculture and Forestry Sciences (116.70°E, 39.71°N). The planting density of all the plots was six plants/m^2 with a row spacing of 60 cm. Morphological representative shoots of each cultivar at 6th leaf (V6), 9th leaf (V9), 13th leaf (V13), and blister (R2) stages [47], were selected and transplanted into pots. Then multi-view images were acquired using the MVS-Pheno platform [18], after which 3D point clouds of the shoots were reconstructed. For validation, twelve shoot point clouds at four growth stages (V3, V6, V9, and V12) were acquired using a 3D scanner (FreeScan X3, Tianyuan Inc., China), to test the segmentation performance of a different data source.

2.2 Overview of the segmentation pipeline

The point cloud of a maize shoot can be segmented into five ~~kinds of~~ instances: stem, leaf, tassel, ear, and pot. The stem, tassel, and pot on a shoot can be regarded as an instance for each. For each transplanted shoot at stage R2, assuming that it contains n_1 ears and n_2 leaves, the point cloud of this shoot can thus be segmented into N=3+n_1+n_2 instances. $\emptyset_{\mathrm{u}}$ represents the point cloud to be segmented, and $\emptyset_{\mathrm{s}}^{i}$ $(i = 1, 2, \ldots\ldots, N)$ represent the ith point cloud instance. In particular, $\emptyset_{\mathrm{s}}^{1}$ and $\emptyset_{\mathrm{s}}^{N}$ refer to the stem and pot (if exists) instance, respectively. Before the segmentation begins, $\emptyset_{\mathrm{u}}$ contains all the points of the shoot, and $\emptyset_{\mathrm{s}}^{i}$ are all empty. With the progression of segmentation, the points in $\emptyset_{\mathrm{u}}$ are gradually assigned to $\emptyset_{\mathrm{s}}^{i}$. The segmentation completes when $\emptyset_{\mathrm{u}}$ is empty.

The segmentation pipeline includes five parts (Figure 1): point cloud down-sampling, stem segmentation, coarse segmentation, fine segmentation, and sample-based segmentation. (1) Point cloud down-sampling. The original input point cloud is down sampled to maintain the shoot morphological features, which improves the segmentation efficiency and quickens the entire segmentation process. (2) Stem segmentation. The top and bottom points of the stem are interactively selected, and the corresponding radius parameters are interactively adjusted. Subsequently, the median region growing is applied to segment the stem points from the shoot automatically. (3) Coarse segmentation. The highest points of each organ instance, except the stem, are obtained via ~~automatic calculation or~~ manual interaction, after which all organ instances are segmented automatically based on the optimal transportation distances. (4) Fine segmentation. The unsatisfactory segmentation point regions are selected interactively, and the seed points of organ instances are selected. Organs are then segmented by Markov random fields (MRF). (5) Sample-based segmentation.

Maize shoots with high-resolution point clouds are segmented based on the fine segmentation result of low-resolution point clouds.

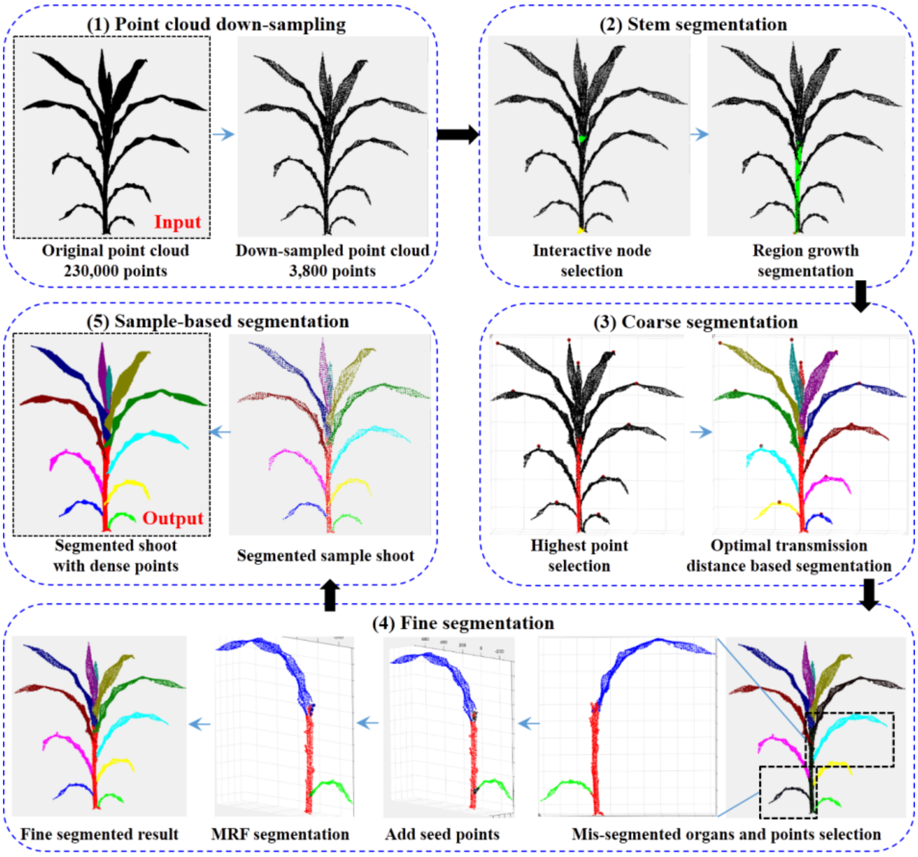

Figure 1: workflow of the segmentation

2.3 Stem segmentation

Two seed points s_0 and s_n at the bottom and top of each stem, were selected interactively. Then, a median-based region growing algorithm [36] was applied to segment the stem points. This segmentation procedure updates the seed point iteratively along the direction from s_0 to s_n. Points around the seed points were classified into stem points. Suppose the algorithm is currently at the kth iteration, and the seed point is s_k, the segmentation process was evaluated as follows:

Step1: Points lying in a sphere were classified as stem points, where s_k is the center of the sphere, r_1 is its radius, and r_1 is a user-specified parameter.

Step 2: The growth direction $\overrightarrow{v_k}$ was determined according to:

$$\overrightarrow{v_k} = (\alpha\overrightarrow{v_1} + \beta\vec{v})/\|\alpha\overrightarrow{v_1} + \beta\vec{v}\|_2$$

$$\overrightarrow{v_1} = median\{(p_A - s_k)/\|p_A - s_k\|_2, p_A \in A\}$$

$$\vec{v} = (s_n - s_k)/\|s_n - s_k\|_2$$

In this formula, $\| \quad \|_2$ is L_2 normal form, and median_{} represents the median operation. α and β

are weight parameters set by users and $\overrightarrow{v_1}$ is the normalized vector from the median of already segmented points of the stem to the seed point s_k. Meanwhile, $\vec{v}$ is the normalized vector from s_k to s_n, which corrects the growth direction to coincide with the stem. In practice, $\alpha = 0.2$ while $\beta = 0.8$. This parameter setting ensures that the stem points can be correctly segmented under different r_1 values, during the entire growing process.

Step 3: A new seed point s_{k+1} for the next iteration was estimated according to $s_{k+1} = s_k + r_1\overrightarrow{v_k}$.

Step 4: Region growing finish condition judgement. Supposing *L* represents the line segment from s_0 to s_n, then project s_{k+1} on *L*. If the projection point was not on *L*, it indicated that the current regional growth was beyond the stem region, and the iteration should be stopped. Otherwise, continue the *k*+1 times iteration and execute step 1.

Because the maize stem gradually thins from bottom to top, a uniform radius r_1 may generate over segmentation, i.e., classifying the points of other organs into the stem. Besides, the region growing algorithm also over segments points in some regions at the bending of the stem. Therefore, a simple median operation was adopted to eliminate the over segmented points. First, the already segmented stem points were evenly divided into *M* segments along the direction of $(s_n - s_0)/\|s_n - s_0\|_2$, and the median axis of each segment was fitted using the least squares. The average distance from each point to the central axis was then calculated. If the distance from a point to the central axis was less than the average distance, it was retained as the stem point; otherwise it was removed from the stem to the unsegmented point set. Users can perform the median operation several times in the toolkit to reduce the over-segmentation problem. Although multiple median operations cause an under segmentation of stem point cloud, the issue is resolved in the subsequent organ segmentation processes. $\emptyset_s^1$ represents the segmented stem points, and these points are removed from $\emptyset_u$. Subsequent organ segmentation is performed in the remaining point cloud. Stem point cloud segmentation is illustrated in Figure 2.

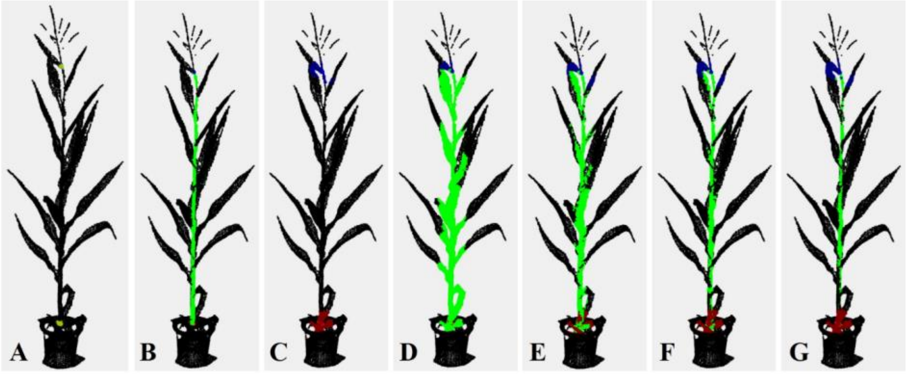

Figure 2: Stem point cloud segmentation. (A) Seed points at the bottom and top of the stem are interactively selected, and an appropriate segmentation radius is set. (B) Stem segmentation result based on (A). (C) A big radius is set. (D) Segmentation result based on (C). (E)-(G) Stem segmentation results with 1, 2, and 3 median operations based on (D).

2.4 Shoot alignment

The shoot points were transformed into a regular coordinate system to access the position of each point in the cloud conveniently. The midpoint of the already segmented stem point cloud was taken as the origin *O* of the new shoot coordinate system. In contrast, the Z-axis of the new coordinate system was the middle axis estimated by the least squares method from the stem point cloud. Then, the shoot point cloud was

projected onto the plane using the Z-axis as its normal vector. The first and second principal component vectors of the projection points were determined by principal component analysis (PCA) and assigned as the X and Y-axis of the new shoot coordinate system, respectively. Subsequently, the original point cloud coordinates were transformed into the new shoot coordinate system, and the coordinates of their *z* value judged the height of points in the shoot. Points are higher with greater *z* values.

2.5 Coarse segmentation of organs

A top-to-down point cloud segmentation algorithm for maize organs from a shoot was applied. The highest point of each organ was taken as the seed point of the organ (Figure 3A). The other shoot points after stem segmentation were classified into corresponding organ instances from top to down by the optimal transportation distances (Figure 3B).

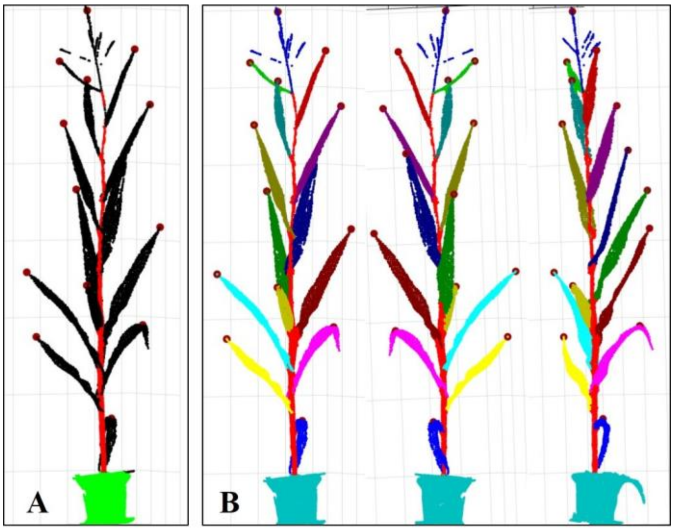

Figure 3: Illustration of coarse segmentation. (A) Highest point determination of each organ. (B) Visualization of segmented shoot from different angles of view.

2.5.1 Organ seed points determination

After stem segmentation, the point cloud of maize shoots was spatially divided into several relatively discrete ~~organs~~ point clouds (excluding the stem). However, the exact organ number is always bigger than the discrete point cloud number, due to the spatial organ connection, especially near the upper leaves. Thus, seed point for each organ has to be determined for the next step segmentation. The highest point of each organ was regarded as the seed point (Figure 3A). If a pot was involved in the point cloud, all points with a *z* value less than the lowest point of the stem were directly classified as pot points. Usually, the highest point of a new leaf appears at the tip region; the middle and lower fully unfolded leaves are mostly curved. Meanwhile, the highest point lies in the middle of the leaf, and the highest points of a tassel or ear are at the top. Therefore, it was assumed that the distance between the highest points of any two organs was more than 5 cm. ~~Therefore~~On this basis, the highest point of each organ was determined by searching for the point with the maximum *z* value within the point cloud of the organ.

Due to the complicated spatial points

For any point p of an organ, we searched its neighbors within a radius of r_z. If the z value of point p was greater than that of its neighboring points, the point was regarded as the highest in the organ. The parameter r_z actually affects the recognition of the highest point of an organ. Too small r_z may cause the highest point found being the local highest point, rather than the global highest point of the current organ. Therefore, r_z was set 1.5 times of the leaf width by default, which allows users to set interactively according to the morphological characteristics of the target shoot.

Numerical experiments show that at the organ connection areas, automatic estimation of the highest points of most organs can be derived by setting the appropriate r_z. However, the algorithm stillinstances has two problems. (1) When the distance between the highest points of adjacent organs in the shoot vary significantly, it is difficult to find a suitable r_z to calculate all complete and accurate highest points. For example, in some shoots, the highest points distance between new emerging leaves is relatively close, while this distance between other leaves is relatively far. (2) Due to the tassel branching structure, each branch has the highest point; multiple highest points of a tassel will be detected using the same algorithm and settings, to ensure the highest points are correctly estimated in other organs. If to ensure only one highest point is calculated in the tassel, the highest point of other organs may be lost.

To solve the problem that the calculation of the highest point of organs may not be accurate.,, Label3DMaize provides a manual interaction module to modify determine the highest seed point of each organ. Simultaneously, this operation can also assign a serial number to each organ for further output. Because the number of maize organs is relatively small, this interactive correction operation is convenient and acceptable. The derived seed points of each organ are set into the corresponding instance point cloud $\emptyset_s^i$. At this time, each leaf, tassel, and ear instance point cloud only contains the highest point, and there are multiple points in the pot and stem instances.

2.5.2 Coarse segmentation based on optimal transportation distances

After obtaining the seed points of all the instances, the left points in $\emptyset_u$ were traversed one by one to determine the instance to which they belong. For each point to $\emptyset_u$, the distance between the point and each other point cloud instance were evaluated, and it was classified into the nearest instance. The classified points were evaluated from top to bottom; that is, the points with bigger z coordinates were evaluated preferentially. The process was as follows:

Step 1: The points in the point set $\emptyset_u$ were reordered from big to small according to their z values.

Step 2: For point $p \in \emptyset_u$, the organ instance it belongs to was determined. The distance d^i from point p to the ith instance was defined as

$$d^i = D_s(p, \widetilde{p^i})$$

Where D_s is the optimal transportation distance between any two points calculated based on the sinkhorn algorithm [48] . Then point p is assigned into the organ instance with the lowest d^i. $\widetilde{p^i}$, in the ith instance, is the nearest neighbor of point p under the optimal transportation distance.

Step 3: Move point p from $\emptyset_u$ into the corresponding $\emptyset_s^i$. Continue traversing the next point in $\emptyset_u$, and perform step 2 until $\emptyset_u$ is empty.

Detailed description of D_s in step 2 is explained here. The optimal transportation strategy of point cloud Q to its identical set Q' is that transmit all the quality of any point $p \in Q$ to the same point $p' \in Q'$. The Sinkhorn algorithm [48] was used here to calculate the optimal transportation distances. It allocates the quality of any point $p \in Q$ to all points in Q'. A point with higher allocation quality suggests the point is closer to p than any other points under the optimal transportation strategy. Suppose that point cloud Q

contains N_Q points. Q' represents the same point set of Q. p_u is the uth point in Q, and M_u indicates the quality of point p_u. Similarly, p'_v is the vth point in Q', and M'_v indicates the quality of point p'_v. m_{uv} represents the transported quality from $p_u \in Q$ to $p'_v \in Q'$. Then the optimal transportation energy from point cloud Q to point cloud Q' can be described as:

$$\underset{m}{\operatorname{argmin}} \sum_{u=1}^{N_Q} \sum_{v=1}^{N_Q} m_{uv} \|p_u - p'_v\| + \frac{1}{\varepsilon} \sum_{u=1}^{N_Q} \sum_{v=1}^{N_Q} m_{uv} \log m_{uv}$$

$$\text{s.t. } m_{uv} > 0; \sum_{v=1}^{N_Q} m_{uv} = M_u; \sum_{u=1}^{N_Q} m_{uv} = M'_v$$

In this equation, ε is the adjusting parameter, which was set to 5 in this paper, and $\| \quad \|$ is the L_2 normal form. The above equation can be solved by Sinkhorn's matrix scaling algorithm [49], and the optimal transportation from Q to Q' can be derived, that is, an $N_Q \times N_Q$ optimal transportation matrix M is obtained. The element m_{uv} at *u* row and *v* column in the matrix is the transported quality from the uth to the vth point. A larger m_{uv} indicates that the two points are closer. After obtaining the optimal transportation solution, the optimal transportation distance from the uth to the vth point in the point cloud can be defined as $D_s(p_u, p_v) = \frac{1}{m_{uv}}$. The pseudocode for calculating the optimal transportation distance *M* is shown in Table 1.

Table 1: The pseudocode for calculating optimal transportation matrix.

Algorithm 1. Computation of optimal transportation matrix *M*, using Matlab syntax.

Input: Parameter ε; Point cloud matrix $Q_{N_Q \times 3}$; % N_Q is the point number of the point cloud

```
n=N_Q;
H_{n×n}=pdist2(Q, Q);  H=H./max(H(:));
K_{n×n}=exp(-εH);
U_{n×n}=K.*H;
a_{n×1}=ones(n,1)/ n;
h_{n×1}=a;
J_{n×n}= diag(1./a)*K;
    while h changes or any other relevant stopping criterion do
   h=1./(J*(a./(h'*K)'));
    end while
z_{n×1}=a./ ((h'*K)');
M_{n×n} = diag(h(:,1)) * K * diag(z(:,1));
```

In the optimal transportation energy equation, when parameter ε increases, the transportation strategy gets closer to the classical optimal transportation, and the segmentation result using optimal transportation distance D_s is also closer to that using Euclidean distance. The same results can be derived using the two distances when the ε is greater than 100. When ε is smaller, the solution becomes smoother, and the nearest neighbour calculated under the D_s distance tends to the region with higher point density. Compared with the Euclidean distance, using the optimal transportation distance to estimate the distance between points can better deal with the challenge of big leaves wrapping on leaflets than using the Euclidean distance (Figure 4A and B). When the adhesion area of the two organs is not significantly large, the segmentation results using the optimal transportation distance is better than that of the Euclidean distance (Figure 4C and D).

Formatted: Font: Italic

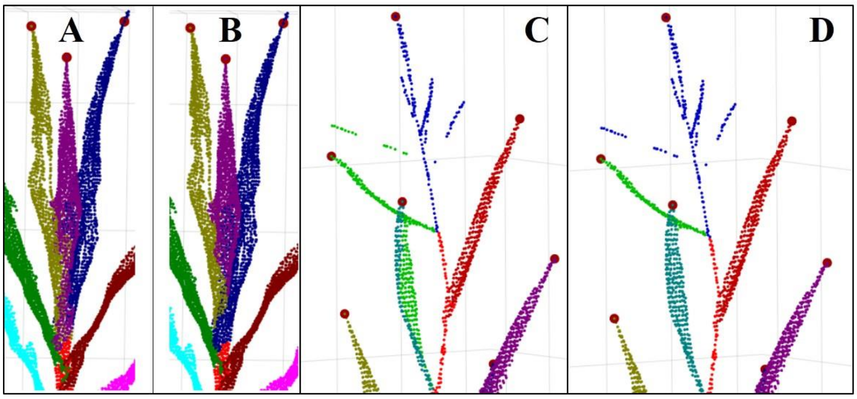

Figure 4: Organ segmentation Comparison using optimal transportation distance and Euclidean distance. Point cloud segmentation result for big leaf wrapping small leaf base case using Euclidean distance (A) and optimal transportation distance (B). Point cloud segmentation result for close or slight organ adhesion case using Euclidean distance (C) and optimal transportation distance (D).

2.6 Fine segmentation of organs

Coarse segmentation can provide preliminary results but false segmentation is frequently observed in the intersecting regions of organs. To obtain more precise segmentation results, this study developed a fine segmentation module for organs in Label3DMaize, which included the following processes:

Step 1: n (n>1) organ instances to be fine segmented were selected, and $\emptyset_{\mathrm{s}'}^{i}$ represents the ith instance.

Step 2: The region of interest was selected among the above instance point cloud, represented by $\emptyset_{\mathrm{us}'}^{i}$.

Step 3: The seed point for the ith instance $\emptyset_{\mathrm{s}'}^{i}$ was selected from region $\emptyset_{\mathrm{u}'}$. The selected points were removed from $\emptyset_{\mathrm{u}'}$ and stored in $\emptyset_{\mathrm{s}'}^{i}$.

Step 4: The points in $\emptyset_{\mathrm{u}'}$ were re-segmented using Markov Random Fields (MRF).

The re-segment algorithm was detailed using MRF in step 4, as explained in the following. The fine segmentation of the interest region mentioned above is a multi-classification problem. It allocates $p_u \in \emptyset_{\mathrm{u}'}$ into n organ instances $\emptyset_{\mathrm{s}'}^{i}$, i.e. search for the right organ tag for point p_u. Hence a mapping function $f_n(p_u)$ is defined for any point p_u. When a point p_u is mapped to the ith instance, $f_n(p_u) = i$, the energy function is defined as:

$$E(f_{\mathrm{n}}) = \gamma \sum_{p_u \in \emptyset_{\mathrm{u}'}} D_{p_u}\big(f_{\mathrm{n}}(p_u)\big) + \sum_{(p_u, q_u) \in \aleph(p_u)} V(f_{\mathrm{n}}(p_u), f_{\mathrm{n}}(q_u))$$

$$D_{p_u}(i) = D\big(p_u, \emptyset_{\mathrm{s}'}^{i}\big) \quad \mathrm{i} = [1,2 \dots \dots \mathrm{n}]$$

$$V\big(f_{\mathrm{n}}(p_u), \mathrm{n}(q_u)\big) = \Big(\frac{d(p_u, q_u)}{d'}\Big)^{\tau}\Big(\frac{a(n_p, n_u)}{\pi}\Big)^{\varphi}$$

In this function, $\aleph(p_u)$ is the k-neighborhood of $p_u \in \emptyset_{\mathrm{u}'}$. The data item $D_{p_u}\big(f_{\mathrm{n}}(p_u)\big)$ measures the loss of classifying p_u to n instances $\emptyset_{\mathrm{s}'}^{i}$. $D(p_u, \emptyset_{\mathrm{s}'}^{i})$ represents the distance from point p_u to instance $\emptyset_{\mathrm{s}'}^{i}$, which is the distance from p_u to the nearest point in $\emptyset_{\mathrm{s}'}^{i}$. γ is a weight parameter that controls the proportion of distance term in the energy function. The smooth item $V(f_{\mathrm{n}}(p_u), f_{\mathrm{n}}(q_u))$ quantifies the corresponding loss when assigning the tag $f_{\mathrm{n}}(p_u)$ and $f_{\mathrm{n}}(q_u)$ for point p_u and q_u, respectively. This smooth term encourages spatial consistency; that is, the probability that adjacent points belong to the same class is higher. The smooth term is composed of the product of the distance term on the left and the angle term on the right. Meanwhile, $d(p_u, q_u)$ is the Euclidean distance of the two points and d' is the maximum

Euclidean distance between all points and their neighbourhood points, regulating the distance term in the range of (0, 1]. n_p and n_u are the normal vectors of points p_u and q_u, respectively. $a(n_p, n_u)$ is the angle between the two normals. τ and φ are the weight parameters for the distance and angle term, respectively, both with a default value of 1.0. The minimum solution of the energy function is solved by α-expansion MRF [50].

In addition, users cloud assign organ label to the region of interest points after the above mentioned step 2, which offers a more direct way for fine segmentation.

2.7 Sample-based segmentation

It is suggested that the number of points per shoot should be less than 15000 to ensure data processing efficiency. Therefore, Label3DMaize provides point cloud simplification and sample-based segmentation modules. Voxel-based simplification is adopted in the toolkit. Sample-based segmentation refers to the automatic segmentation of dense point cloud via the segmentation result of the corresponding simplified point cloud. Specifically, suppose that point cloud *A* is the simplification of dense point cloud *B*, and *A* has already been segmented while *B* is to be segmented. Calculating the *k*-nearest neighbors in *A* of any point $p \in B$, and then counts how many points of these *k*-nearest neighbors belong to each instance. The instance with the maximum neighbour points is determined as the instance of point *p*.

3 Results

3.1 Interface and operations of Label3DMaize

The Label3DMaize toolkit was developed using Matlab. The interface is composed of the main interface and multiple sub-interfaces, including stem segmentation, coarse segmentation, fine segmentation, and sample-based segmentation (Figure 5). Each sub-interface is popped up after the corresponding button on the main interface is triggered. The main interface and each sub-interface are composed of an embedded dialog and an interactive visual window (only the embedded dialog in each sub-interface is shown in Figure 5). The interactive visual window enables the users to rotate, zoom, translate, select interested points in the view, and improve the segmentation effect visually and interactively. The input of the toolkit includes point cloud files in text format, such as txt or ply. According to the operational process shown in Figure 5, segmentation results can be refined step by step by inputting parameters and manually selecting points. The output of the toolkit is a text file with annotation information; that is, each 3D coordinate point in the text has a classification identification number, and the points with the same identification number belong to the same instance. This format files are applicable for 3D deep learning of maize shoots. The executable program of Label3DMaize can be found in the attachment.

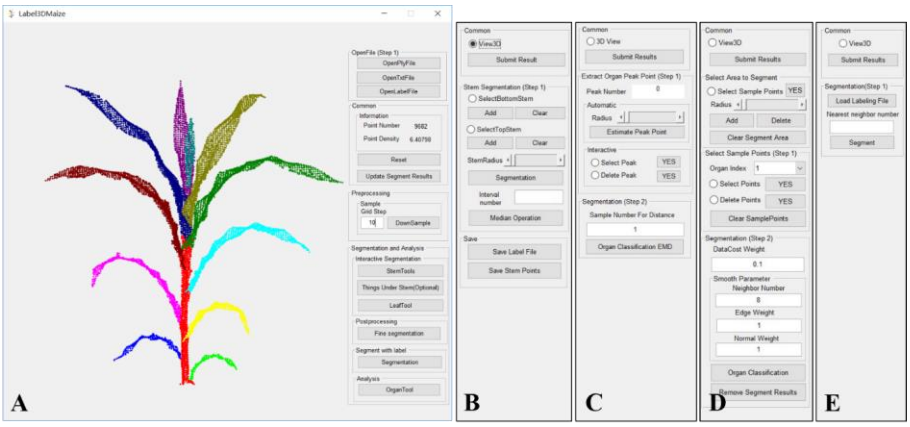

Figure 5: Interfaces of Label3DMaize. (A) The main interface of the toolkit, composed of a visualization window and an embedded dialog. (B)-(E) Dialog of stem segmentation, coarse segmentation, fine segmentation, and sample-based segmentation. The visualization window is not shown in these sub-interfaces.

3.2 Visualization and accuracy evaluation

To evaluate coarse and fine segmentation accuracy, the point clouds of three varieties in four different growth stages of maize shoots are segmented using Label3DMaize. Figure 6 shows the visualization results. According to the visualization results, no significant differences were observed between the coarse and fine segmentation. Yet, fine segmentation improved the segmentation effect of the details, especially near the connection region of organs.

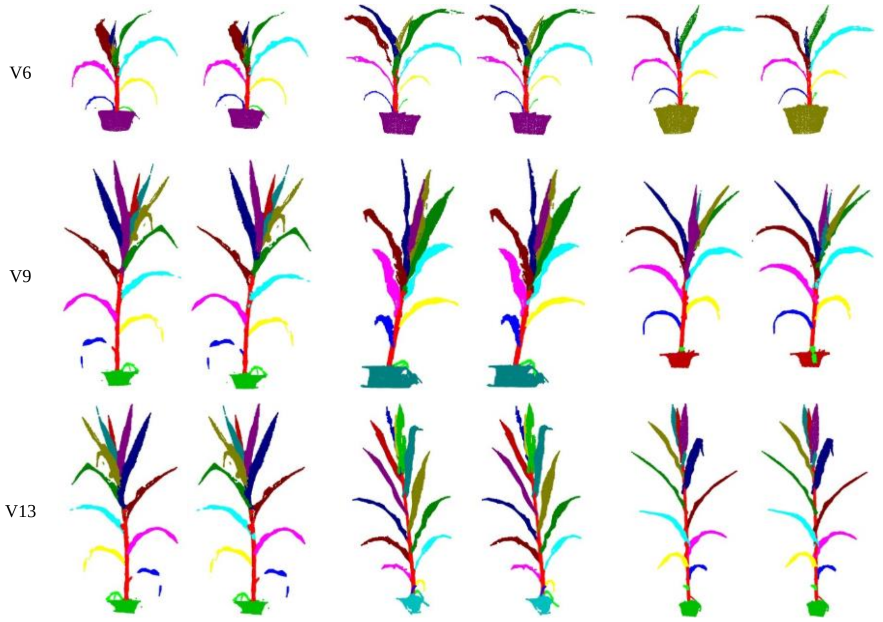

R2

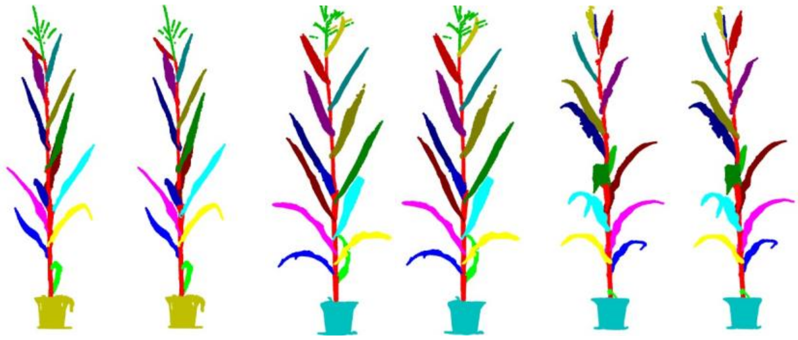

MC670 NK815 XY335

Figure 6 Visualization of maize shoot segmentation results of three cultivars at four growth stages. In each sub-figure, the left and right are coarse and corresponding fine segmentation results, respectively.

This study has further provided numerical accuracy results to quantitatively evaluate the difference between coarse and fine segmentation (Table 2). The precision, recall and F1-score of each organ were estimated based on fine segmentation as the ground truth. The averaged precision and recall of all shoot organs were taken as the precision and recall. Macro-F1 and micro-F1 are calculated using the precision and recall of the shoot and organs averaged value, respectively. It can be seen from Table 2 that although the accuracy of coarse and fine segmentation differed, the overall difference was not significant.

Table 2: Accuracy evaluation of coarse and fine segmentation

	Overall accuracy	Precision	Recall	Micro-F1	Macro-F1
Mean	0.972099	0.967282	0.956173	0.961458	0.955593
Min	0.897683	0.91954	0.841063	0.878553	0.853139
Max	0.993867	0.991753	0.991315	0.991534	0.991175

3.3 Segmentation efficiency

The efficiency of plant point cloud segmentation is an essential indicator for the practicality for training data annotation tools for deep learning. Table 3 shows the time consumed in the different steps for maize shoot segmentation at four growth stages using Label3DMaize on a workstation (Intel Core i7 processor, 3.2GHz CPU, 32GB of memory, Windows 10 operating system), including the interactive manual operations and segmentation computations. It can be seen that point cloud segmentation takes about 4-10 minutes per shoot, in which coarse segmentation takes about 10%-20% of the total time. In the whole segmentation process, the manual interaction time cost is significantly higher than that of automated computation. The segmentation efficiency is positively related to the number of leaves.

This study also analyzed the detailed time costs. (1) The time cost of stem segmentation. In the early growth stages of a maize shoot, the stem is relatively upright, so users only need to select the bottom and upper points of the stem and specify a suitable radius. However, in the late growth stages, the maize shoot height becomes higher, and the stem becomes thinner from bottom to top. Meanwhile, the upper part ~~curves~~is curved, so interactive median segmentation is needed, which increases the segmentation time. (2) The time cost of coarse segmentation. The major interactive operation of coarse segmentation is that the user selects or adjusts the highest organ points. As the maize shoot grows, the number of organs gradually increases, so the time costs for the interactive operation of picking points also increases. Meanwhile, the growth of shoot organs significantly increases the occlusion among organs. Thus, the appropriate angles of view for users

have to be found to determine the highest organ points, which is time-consuming. (3) The time cost of fine segmentation. An increase in the number of organs causes false segmentation of more organs at the connection regions. Therefore, the fine segmentation of maize shoots with more organs would take more time. Besides, the segmentation efficiency is related to the shoot architecture; the spatial distances between adjacent organs are much larger in flattened shoots than that of relatively compact ones, which increases the segmentation efficiency of flattened shoots.

Table 3: Segmentation time of different steps on maize shoots at four growth stages using Label3DMaize

Growth period	Point number of a maize shoot		Time cost (s)									
	Input	After simplification	t_1	t_2	t_3	t_4	t_5	t_6	t_7	t_8	t_9	T
V6	45833	13196	10	0.2	16	4	30.2	~~120~~60	0.05	0.5	100	**~~250~~190.75**
V9	62523	13953	10	0.2	21	4	35.2	~~220~~140	0.05	0.6	100	**~~355~~275.85**
V13	70873	12102	14	0.2	32	5	51.2	~~400~~260	0.05	0.6	100	**~~551~~411.85**
R2	71909	13224	14	0.2	35	5	54.2	~~400~~268	0.05	0.6	100	**~~554~~422.85**

* t_1: Time for stem point selection and radius setting. t_2: Time for segmentation computation of stem points. t_3: Time for seed points selection of organ instances. t_4: Time for organ segment computation. t_5: Time for coarse segmentation, where $t_5 = t_1+t_2+t_3+t_4$. t_6: Time for fine segmentation operations. t_7: Time for fine segmentation computation. t_8: Time for sample-based segmentation. t_9: Time for other operations, e.g., the alternation between main and sub-interfaces. T: Total time costs. Underlined and un-underlined identifiers indicate~~s~~ the time cost for manual interactions and automated computation respectively.

3.4 Comparison with other methods

Method comparison PointNet-based segmentation and , was conducted to evaluate the algorithm performance in coarse segmentation. The point cloud data used here consisted of twelve shoots obtained from the 3D scanner (mentioned in the data acquisition section). Region growing in Point Cloud Library (PCL) [51] and PointNet-based segmentation, are considered as the state-of-the-art methods for comparison. ~~[51]~~The best segmentation result was obtained through parameter exhaustion for each shoot using region growing. For PointNet-based segmentation [52], a training dataset containing 1000 labeled maize shoots was built using Label3DMaize. The PointNet model was then trained, and the segmentation model was derived. The segmentation accuracy is shown in Table 4, and representative results of each growth stage are shown in Figure 7. The fine segmentation results derived using Label3DMaize were regarded as the well-segmented reference for comparison. Results showed that Label3DMaize could deal with MVS reconstructed point clouds and also handle the point cloud derived using 3D scanner. Region growing is oriented to solve general segmentation problems; the segmentation effect is obviously different from the other two methods in maize point cloud segmentation. Thus, the efficiency of region growing is less than that of PointNet and coarse segmentation. The segmentation result of coarse segmentation presented in this paper is more accurate than that of PointNet. A~~Figure 7~~ lthough the PointNet model can realize automatic segmentation compared with the rough segmentation containing interaction in this paper, dealing with many details could be challenging. For instance, it is difficult to accurately extract the point cloud at the stem and leaf boundary, segmenting big leaf wrapping small leaf at the shoot top could be challenging, and it always ignores the newly emerged leaves.

Table 4: ~~–~~Accuracy comparison of region growing, PointNet, and coarse segmentation.

	Overall accuracy	Precision	Recall	Micro-F1	Macro-F1

Formatted: Indent: First line: 2 ch

Formatted Table

Region growing	0.7910	0.7472	0.7530	0.7679	0.8053
PointNet	0.9264	0.9261	0.9261	0.9186	0.9074
Coarse segmentation	0.9924	0.9896	0.9906	0.9901	0.9898

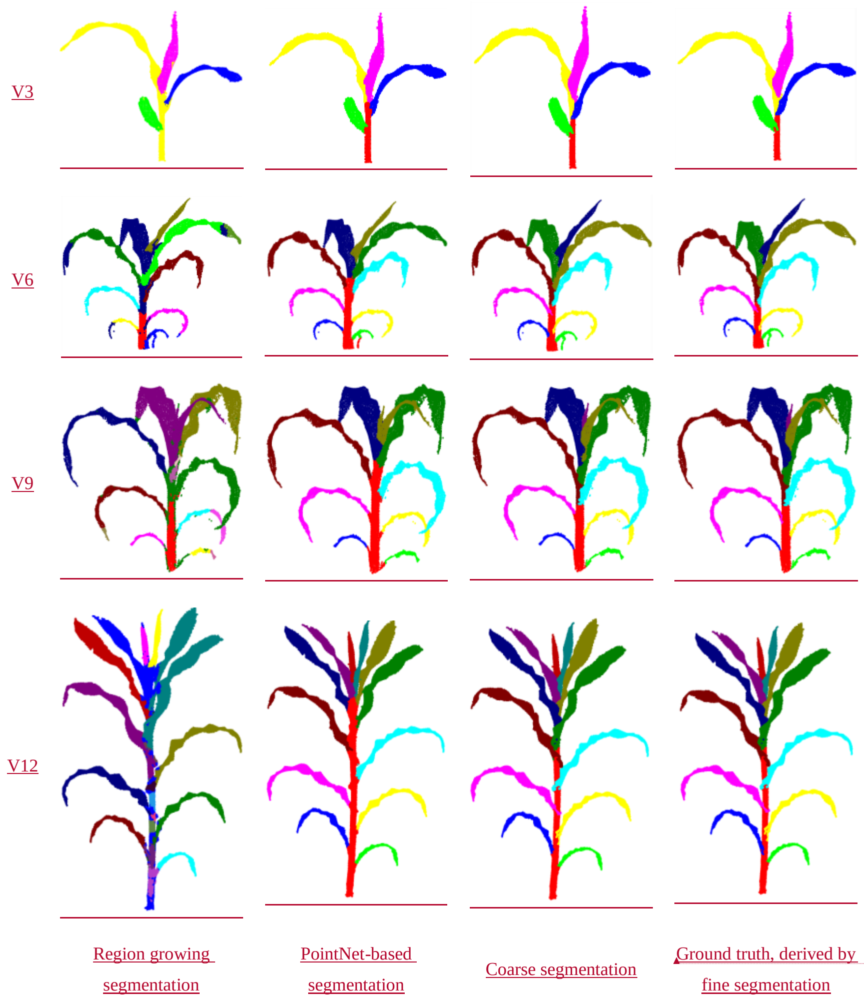

Figure 7 Visualization of segmentation results using region growing, PointNet, coarse segmentation, and fine segmentation.

3.5 Performance on other plants

This study determined the performance of Label3DMaize in segmenting other plants with only one main stem, including tomato, cucumber, and wheat.

Two types of segmentation have been conducted on tomato in literature [11]; the first (Type I) treats a big leaf with several small leaves as a cluster leaf, while the second (Type II) treats each big or small leaf as independent. This study aimed to realize these two type forms using Label3DMaize. The type I segmentation result (Figure 8B) was derived by selecting the highest point of each leaf cluster (Figure 8A) in the coarse

Formatted

Formatted

Formatted

Formatted

Formatted: Font: 9 pt, Font color: Auto

Formatted

Formatted: Indent: First line: 2 ch, Space Before: 0 pt, After: 0 pt

segmentation procedure and details were enhanced by fine segmentation (Figure 8C). For type II segmentation, the highest points of all the leaves have to be specified (Figure 8D). Consequently, coarse and fine segmentation could be derived (Figure 8E and F). The segmentation method used in [11] is based on a machine learning model; thus, it can only segment trained plants. In contrast, Label3DMaize has better generality.

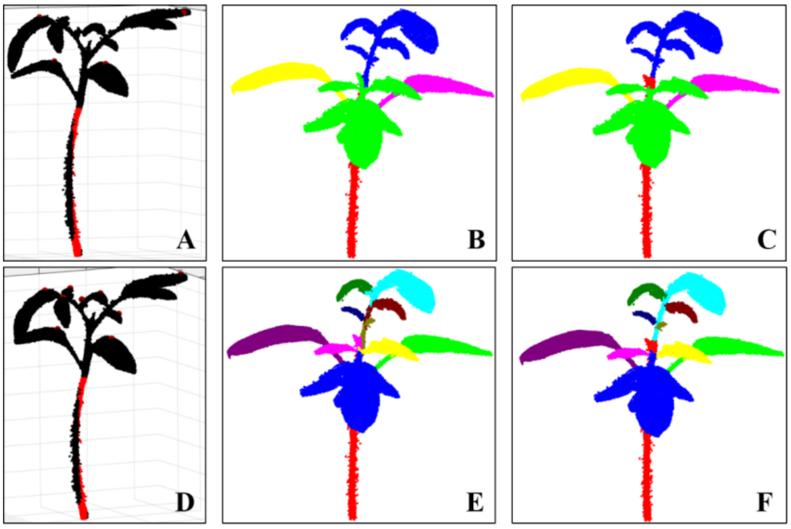

Figure 8 Performance evaluation of Label3DMaize on tomato for two types of segmentation. Type I: leaf cluster segmentation (A-C). Type II: individual leaf segmentation (D-F). A and D illustrate the highest point selection in the two forms of coarse segmentation. B and E show the coarse segmentation results. C and F are fine segmentation results.

Cucumber was selected as a plant representative to test the segmentation performance of Label3DMaize on plants with a soft stem. Different from the topological structure of maize, cucumber has larger stem curvature and has leaf petioles. Thus, the interactive endpoints selection for stem segmentation of cucumber differs from maize. Selection of the highest point of cucumber stem is similar to maize. When selecting the other stem endpoint, we could find the lowest point that coincides with the straight-line direction from the stem top to bottom (Figure 9A). Although the unselected stem part will be missing, it can be completed in the subsequent coarse segmentation (Figure 9B). The coarse segmentation and directly fine segmentation tend to segment each leaf and corresponding petiole into an individual organ (Figure 9C). Separated petiole and leaf can be obtained by fine segmentation, which segments all the petioles and a single stem as a whole (Figure 9D).

Formatted: Indent: First line: 2 ch

Formatted: Indent: First line: 2 ch, Space Before: 0 pt, After: 0 pt

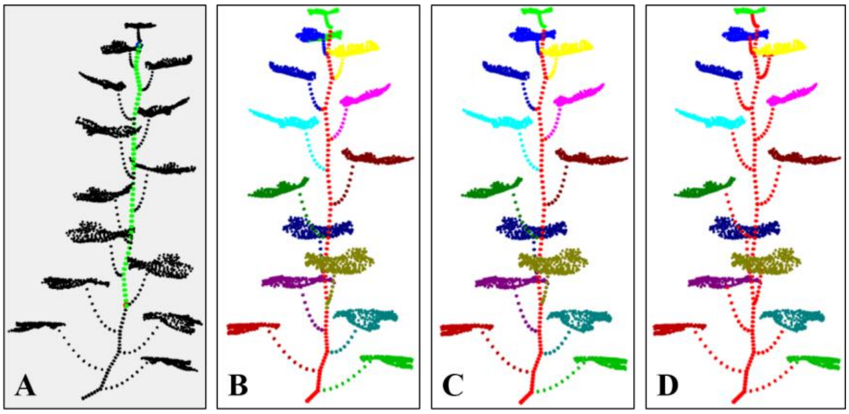

Figure 9 Visualization of cucumber point cloud segmentation. A: Illustration of the lowest and highest selection points in stem segmentation. B: Coarse segmentation result. C: Fine segmentation. Each leaf and corresponding petiole are classified as an instance. D: Fine segmentation. All the petioles and the main stem are classified as an instance.

A point cloud of wheat shoot at the early growth stage was acquired using the MVS-Pheno platform. Because the wheat shoot is small with a thin stem, the tiller points are fused together near the shoot base. However, the tiller tops could be identified, which enables segmentation of the wheat shoot by Label3DMaize. For plants with tillers, only one stem is selected in the stem segmentation procedure (Figure 10A). When selecting the organ's highest points in coarse segmentation, not only the highest point of each leaf but also the highest point of each tiller has to be selected (Figure 10B). Coarse segmentation can ensure a better effect of leaf segmentation (Figure 10C). However, tillers and stem are prone to under segmentation, which need to be adjusted by fine segmentation (Figure 10D).

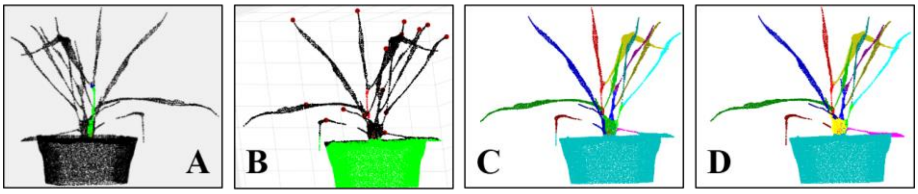

Figure 10 Visualization of wheat shoot segmentation using Label3DMaize. A: Stem points selection. B: Selection of highest points in leaves and tillers. C: Coarse segmentation. D: Fine segmentation.

4 Discussion

4.1 Shoot-organ point cloud segmentation

Most non-destructive 3D data acquisition of plants focus on individual plant scale. Thus point cloud segmentation from shoot to organ is of significance. Representative shoot-organ point cloud segmentation is realized by region growing combined with adjusting leaf number and stem diameter parameters according to the shoot architecture and stem morphological features. In representative shoot-organ segmentation approaches [36], Lleaf overlap challenges shoot segmentation, especially for upper leaves in compact shoot

architecture. ~~Besides, the robustness of the segmentation algorithm also needs to be verified when processing many point clouds.~~ Once the segmentation is complete, it is difficult to correct the false segmentation points. Although commercial software, such as Geomagic Studio, can solve this problem, it is quite complicated and time-consuming. In contrast, the Label3DMaize toolkit integrates a top-to-down segmentation algorithm and interactive operations according to the morphological structure of maize shoots, which can realize semi-automatic fine point cloud segmentation. The top-to-down coarse segmentation ensures topological accuracy, and the interactive operations improve the segmentation accuracy and details. Although coarse segmentation can meet the basic demand for phenotype extraction, it is not satisfactory for high-precision phenotypic analysis and 3D reconstruction based on point clouds. In contrast, fine segmentation is more satisfactory for the latter demands. The toolkit can solve the point cloud segmentation problem of compact architecture or organ overlapping shoots. Although skeleton extraction methods [34, 35] also provide an interactive way to improve the segmentation accuracy, they offer skeleton interaction, which hardly improves the segmentation point details.

Since 3D point cloud annotation tools for plants are lacking, researchers segment plants through multi-view image label~~l~~ing, deep learning-based image segmentation, MVS reconstruction, and a voting strategy [53]. However, these methods cause a lot of organ occlusion from different view angles; thus, it is hard to segment plants with multiple organs through image labe~~l~~ling and MVS reconstruction. Jin et al. [37] transformed point cloud data into a voxel format, constructed a training set containing 3000 maize shoots via data enhancement, and proposed a convolutional neural network (VCNN) to segment stem and leaf point cloud of maize shoots. Label3DMaize enables researchers to directly handle 3D point cloud segmentation and data annotation without transforming point cloud data into the voxel form. Meanwhile, using the acquired data directly improves the diversity of training set data, rather than by data enhancement, and can thus improve the robustness of the learned model. In addition, label3DMaize can separate the tassel and ear except for the stem and leaf, facilitating phenotype extraction of the tassel (such as the number of tassel branches, the compactness of tassel, etc.) and ears (such as the ear height).

4.2 Practicability of Label3DMaize

In our recent works, the MVS-Pheno platform [18] ~~has been~~was used to obtain high-throughput 3D point cloud data of maize shoots at different ecological sites for various genotypes and growth stages. However, the underlying knowledge about genotypes and the differences in cultivation management have not been fully explored, indicating that high-throughput phenotypic acquisition is far from practical application. Therefore, it is urgent to establish automatic and online data analysis approaches [54]. However, due to the complexity of plant morphological structure, it is difficult to realize automatic 3D segmentation from the plant morphological characteristics and regional growth method only. Deep learning is a feasible way to realize automatic segmentation by mining deep features of plant morphology. The greatest challenge in 3D point cloud segmentation by deep learning is the lack of high precision and efficient data annotation tools. Most of the existing 3D data annotation methods are for voxel data [37, 55], not 3D point clouds. Thus, Label3DMaize provides a practical tool for 3D point cloud data annotation for maize and could be a reference for other plants. It has been demonstrated that the toolkit can segment or label other plants, such as tomato, cucumber, and wheat. Coarse segmentation, i.e., the top-to-down point cloud segmentation algorithm using optimal transportation distance, suits plants with a single stem. Meanwhile, if a plant has too many organs, selecting all the highest points of each organ is rather complicated. Above all, interactive operations in fine segmentation enable extension of the toolkit to other specific plants. Specifically, Label3DMaize does not depend on data generated through MVS-Pheno. Any point cloud of maize shoot can be the toolkit input, including data acquired using 3D scanners (Figure 7), or reconstructed from multi-view images acquired by

handheld cameras.

Unlike RGB image data annotation [40], data enhancement does not that significantly improve the model robustness of 3D point cloud segmentation models. Thus high-quality data annotation is important. It takes 4-10 minutes to label a maize shoot point cloud by Label3DMaize, and this labeling efficiency can meet the needs of constructing a training dataset for deep learning. The fine segmentation module in Label3DMaize ensures accurate segmentation of detailed features at the organ connections, and is thus satisfactory for organ-level 3D reconstruction. Of noteIf high precision of the annotation is not required, coarse segmentation results can be used as the annotation data if high precision of the annotation is not required, thus saving a lot of time.

Label3DMaize is designed for individual shoots and does not support segmentation of multiple maize shoots. Thus, point clouds containing multiple shoots have to be preprocessed into individual shoot point clouds first, through spatial connection property of points, or interactively separated using commercial software (such as CloudCompare, Geomagic Studio, etc.). This shoot separation preprocess is easy for scenarios without cross organs. Thus, point cloud data acquisition is important for subsequent segmentation. Point clouds with less noise are required when using Label3DMaize, so the toolkit is more suitable for segmenting point clouds derived by MVS reconstruction. For shoots with much random noise obtained by 3D scanners [35], point cloud denoising should be performed first, and then set as the toolkit input of the toolkit for segmentation. Compared with image annotation, the data annotation efficiency of Label3DMaize is still lower, and fine segmentation requires more manual interaction, which has higher requirements for user experience and concentration. Thus the algorithm for Label3DMaize needs improvement to raise the automation level of point cloud segmentation.

4.3 Future work

At present, a large amount of 3D point cloud data of maize shoots has been obtained using MVS-Pheno. In our future study, representative data will be selected and annotated by Label3DMaize, then a 3D maize shoot annotation dataset will be constructed. A deep learning-based point cloud segmentation model will then be developed to realize the automatic segmentation of maize shoots. What's more, well segmented maize organ data could be used to build a 3D shape model of maize. All the above technologies or data will conversely simplify the segmentation and labeling processes of the toolkit. Subsequently, online phenotypic extraction and 3D reconstruction of maize shoots algorithms will be studied using the well-segmented point clouds. The segmentation algorithm and this toolkit will be extended to other crops according to their morphological characteristics, which will promote the automatic 3D point cloud segmentation of plants.

Additional files

Supplementary Program. Executable program of Label3DMaize, which requires that Matlab runtime (Version 9.2 or above) installed.

Supplementary Data S1. The acquired point clouds of maize shoots described in Section "Field experiment and data acquisition", also as the input of the programFigure 6, including the point clouds acquired using MVS-Pheno, coarse segmentation results, fine segmentation results, and sample-based segmentation results.

Supplementary Data S2. Coarse segmentation results of the input shootsPoint cloud data described in Figure 7. These point clouds are acquired using a 3D scanner.

Supplementary Data S3. Fine segmentation results, derived based on the coarse onesSegmentation results on other plants, including tomato data described in Figure 8, cucumber data described in Figure 9, and wheat data described in Figure 10.

Supplementary Data S4. Sample based segmentation results, derived from the fine segmentation results.

Acknowledgements

This work was partially supported by Construction of Collaborative Innovation Center of Beijing Academy of Agricultural and Forestry Sciences (KJCX201917), Science and Technology Innovation Special Construction Funded Program of Beijing Academy of Agriculture and Forestry Sciences(KJCX20210413), the National Natural Science Foundation of China (31871519, 32071891), Reform and Development Project of Beijing Academy of agricultural and Forestry Sciences, China Agriculture Research System (CARS-02). We would like to thank Tianjun Xu, in Maize Research Center of Beijing Academy of agricultural and Forestry Sciences, for providing experimental materials.

References

1. Bucksch A, Atta-Boateng A, Azihou AF, Battogtokh D, Baumgartner A, Binder BM, et al. Morphological Plant Modeling: Unleashing Geometric and Topological Potential within the Plant Sciences. Front Plant Sci. 2017;8:16. doi:10.3389/fpls.2017.00900.

2. Gibbs JA, Pound M, French AP, Wells DM, Murchie E and Pridmore T. Approaches to three-dimensional reconstruction of plant shoot topology and geometry. Funct Plant Biol. 2017;44 1:62-75. doi:10.1071/FP16167.

3. Lin Y. LiDAR: An important tool for next-generation phenotyping technology of high potential for plant phenomics? Comput Electron Agric. 2015;119:61-73. doi:10.1016/j.compag.2015.10.011.

4. Zhao C, Zhang Y, Du J, Guo X, Wen W, Gu S, et al. Crop Phenomics: Current Status and Perspectives. Front Plant Sci. 2019;10:714. doi:10.3389/fpls.2019.00714.

5. Perez-Sanz F, Navarro PJ and Egea-Cortines M. Plant phenomics: an overview of image acquisition technologies and image data analysis algorithms. GigaScience. 2017;6 11:18. doi:10.1093/gigascience/gix092.

6. Pieruschka R and Schurr U. Plant Phenotyping: Past, Present, and Future. Plant Phenomics. 2019;2019:6. doi:10.1155/2019/7507131.

7. Rahman A, Mo C and Cho B-K. 3-D Image Reconstruction Techniques for Plant and Animal Morphological Analysis-A Review. Journal of Biosystems Engineering. 2017;42 4:339-49.

8. Vos J, Evers JB, Buck-Sorlin GH, Andrieu B, Chelle M and de Visser PHB. Functional-structural plant modelling: a new versatile tool in crop science. J Exp Bot. 2010;61 8:2101-15. doi:10.1093/jxb/erp345.

9. Louarn G and Song Y. Two decades of functional–structural plant modelling: now addressing fundamental questions in systems biology and predictive ecology. Ann Bot. 2020;126 4:501-9. doi:10.1093/aob/mcaa143.

10. Jin SC, Sun XL, Wu FF, Su YJ, Li YM, Song SL, et al. Lidar sheds new light on plant phenomics for plant breeding and management: Recent advances and future prospects. ISPRS-J Photogramm Remote Sens. 2021;171:202-23. doi:10.1016/j.isprsjprs.2020.11.006.

11. Ziamtsov I and Navlakha S. Machine Learning Approaches to Improve Three Basic Plant Phenotyping Tasks Using Three-Dimensional Point Clouds. Plant Physiol. 2019;181 4:1425-40. doi:10.1104/pp.19.00524.

12. Rist F, Herzog K, Mack J, Richter R, Steinhage V and Töpfer R. High-Precision Phenotyping of Grape Bunch Architecture Using Fast 3D Sensor and Automation. Sensors. 2018;18 3:763. doi:10.3390/s18030763.

13. Thapa S, Zhu F, Walia H, Yu H and Ge Y. A Novel LiDAR-Based Instrument for High-Throughput, 3D Measurement of Morphological Traits in Maize and Sorghum. Sensors. 2018;18 4:1187. doi:10.3390/s18041187.

14. Hu Y, Wang L, Xiang L, Wu Q and Jiang H. Automatic Non-Destructive Growth Measurement of Leafy Vegetables Based on Kinect. Sensors. 2018;18 3:806. doi:10.3390/s18030806.

15. Chaivivatrakul S, Tang L, Dailey MN and Nakarmi AD. Automatic morphological trait characterization for corn plants via 3D holographic reconstruction. Comput Electron Agric. 2014;109:109-23. doi:j.compag.2014.09.005.

16. Elnashef B, Filin S and Lati RN. Tensor-based classification and segmentation of three-dimensional point clouds for organ-level plant phenotyping and growth analysis. Comput Electron Agric. 2019;156:51-61. doi:https://doi.org/10.1016/j.compag.2018.10.036.

17. Duan T, Chapman SC, Holland E, Rebetzke GJ, Guo Y and Zheng B. Dynamic quantification of canopy structure to characterize early plant vigour in wheat genotypes. J Exp Bot. 2016;67 15:4523-34. doi:10.1093/jxb/erw227.

18. Wu S, Wen W, Wang Y, Fan J, Wang C, Gou W, et al. MVS-Pheno: A Portable and Low-Cost Phenotyping Platform for Maize Shoots Using Multiview Stereo 3D Reconstruction. Plant Phenomics. 2020;2020:17. doi:10.34133/2020/1848437.

19. Nguyen CV, Fripp J, Lovell DR, Furbank R, Kuffner P, Daily H, et al. 3D Scanning System for Automatic High-Resolution Plant Phenotyping. *2016 International Conference on Digital Image Computing: Techniques and Applications (DICTA)*. 2016, p. 1-8.

20. Cao W, Zhou J, Yuan Y, Ye H, Nguyen HT, Chen J, et al. Quantifying Variation in Soybean Due to Flood Using a Low-Cost 3D Imaging System. Sensors. 2019;19 12:2682. doi:10.3390/s19122682.

21. Bernotas G, Scorza LCT, Hansen MF, Hales IJ, Halliday KJ, Smith LN, et al. A photometric stereo-based 3D imaging system using computer vision and deep learning for tracking plant growth. GigaScience. 2019;8 5:15. doi:10.1093/gigascience/giz056.

22. Zhang XH, Huang CL, Wu D, Qiao F, Li WQ, Duan LF, et al. High-Throughput Phenotyping and QTL Mapping Reveals the Genetic Architecture of Maize Plant Growth. Plant Physiol. 2017;173 3:1554-64. doi:10.1104/pp.16.01516.

23. Cabrera-Bosquet L, Fournier C, Brichet N, Welcker C, Suard B and Tardieu F. High-throughput estimation of incident light, light interception and radiation-use efficiency of thousands of plants in a phenotyping platform. New Phytol. 2016;212 1:269-81. doi:10.1111/nph.14027.

24. Jin S, Su Y, Song S, Xu K, Hu T, Yang Q, et al. Non-destructive estimation of field maize biomass using terrestrial lidar: an evaluation from plot level to individual leaf level. Plant Methods. 2020;16 1:69. doi:10.1186/s13007-020-00613-5.

25. Zermas D, Morellas V, Mulla D and Papanikolopoulos N. 3D model processing for high throughput

phenotype extraction – the case of corn. Comput Electron Agric. 2019:105047. doi:https://doi.org/10.1016/j.compag.2019.105047.

26. Jin SC, Su YJ, Gao S, Wu FF, Hu TY, Liu J, et al. Deep Learning: Individual Maize Segmentation From Terrestrial Lidar Data Using Faster R-CNN and Regional Growth Algorithms. Front Plant Sci. 2018;9:10. doi:10.3389/fpls.2018.00866.

27. Zhan Q, Liang Y and Xiao Y. Color-based segmentation of point clouds. *Laser scanning*. 2009, p. 248-52.

28. Itakura K and Hosoi F. Automatic Leaf Segmentation for Estimating Leaf Area and Leaf Inclination Angle in 3D Plant Images. Sensors. 2018;18 10:3576.

29. Sun SP, Li CY, Chee PW, Paterson AH, Jiang Y, Xu R, et al. Three-dimensional photogrammetric mapping of cotton bolls in situ based on point cloud segmentation and clustering. ISPRS-J Photogramm Remote Sens. 2020;160:195-207. doi:10.1016/j.isprsjprs.2019.12.011.

30. Li D, Shi G, Kong W, Wang S and Chen Y. A Leaf Segmentation and Phenotypic Feature Extraction Framework for Multiview Stereo Plant Point Clouds. IEEE Journal of Selected Topics in Applied Earth Observations and Remote Sensing. 2020;13:2321-36. doi:10.1109/JSTARS.2020.2989918.

31. Paulus S, Dupuis J, Mahlein AK and Kuhlmann H. Surface feature based classification of plant organs from 3D laserscanned point clouds for plant phenotyping. BMC Bioinformatics. 2013;14:12. doi:http://dx.doi.org/10.1186/1471-2105-14-238.

32. Wahabzada M, Paulus S, Kersting K and Mahlein AK. Automated interpretation of 3D laserscanned point clouds for plant organ segmentation. BMC Bioinformatics. 2015;16:11. doi:10.1186/s12859-015-0665-2.

33. Li YY, Fan XC, Mitra NJ, Chamovitz D, Cohen-Or D and Chen BQ. Analyzing Growing Plants from 4D Point Cloud Data. ACM Trans Graph. 2013;32 6:10. doi:10.1145/2508363.2508368.

34. Xiang LR, Bao Y, Tang L, Ortiz D and Salas-Fernandez MG. Automated morphological traits extraction for sorghum plants via 3D point cloud data analysis. Comput Electron Agric. 2019;162:951-61. doi:10.1016/j.compag.2019.05.043.

35. Wu S, Wen W, Xiao B, Guo X, Du J, Wang C, et al. An Accurate Skeleton Extraction Approach From 3D Point Clouds of Maize Plants. Front Plant Sci. 2019;10:248. doi:10.3389/fpls.2019.00248.

36. Jin SC, Su YJ, Wu FF, Pang SX, Gao S, Hu TY, et al. Stem-Leaf Segmentation and Phenotypic Trait Extraction of Individual Maize Using Terrestrial LiDAR Data. IEEE Trans Geosci Remote Sensing. 2019;57 3:1336-46. doi:10.1109/tgrs.2018.2866056.

37. Jin S, Su Y, Gao S, Wu F, Xu K, Ma Q, et al. Separating the Structural Components of Maize for Field Phenotyping Using Terrestrial LiDAR Data and Deep Convolutional Neural Networks. IEEE Trans Geosci Remote Sensing. 2019;58 4:2644 - 58. doi:10.1109/TGRS.2019.2953092.

38. Griffiths D and Boehm J. A Review on Deep Learning Techniques for 3D Sensed Data Classification. Remote Sensing. 2019;11 12:29. doi:10.3390/rs11121499.

39. Engelmann F, Bokeloh M, Fathi A, Leibe B and Nießner M. 3D-MPA: Multi-Proposal Aggregation for 3D Semantic Instance Segmentation. In: *2020 IEEE/CVF Conference on Computer Vision and Pattern*

Recognition (CVPR) 13-19 June 2020 2020, pp.9028-37.

40. Russell BC, Torralba A, Murphy KP and Freeman WT. LabelMe: A database and web-based tool for image annotation. Int J Comput Vis. 2008;77 1-3:157-73. doi:10.1007/s11263-007-0090-8.

41. Chang AX, Funkhouser T, Guibas L, Hanrahan P, Huang Q, Li Z, et al. ShapeNet: An Information-Rich 3D Model Repository. Computer ence. 2015.

42. Hackel T, Savinov N, Ladicky L, Wegner JD, Schindler K and Pollefeys M. Semantic3D.net: A new Large-scale Point Cloud Classification Benchmark. 2017.

43. Behley J, Garbade M, Milioto A, Quenzel J, Behnke S, Stachniss C, et al. SemanticKITTI: A Dataset for Semantic Scene Understanding of LiDAR Sequences. In: *2019 IEEE/CVF International Conference on Computer Vision (ICCV)* 27 Oct.-2 Nov. 2019 2019, pp.9296-306.

44. Ku T, Veltkamp RC, Boom B, Duque-Arias D, Velasco-Forero S, Deschaud JE, et al. SHREC 2020: 3D point cloud semantic segmentation for street scenes. Comput Graph-UK. 2020;93:13-24. doi:10.1016/j.cag.2020.09.006.

45. Dutagaci H, Rasti P, Galopin G and Rousseau D. ROSE-X: an annotated data set for evaluation of 3D plant organ segmentation methods. Plant Methods. 2020;16 1:14. doi:10.1186/s13007-020-00573-w.

46. Gené-Mola J, Sanz-Cortiella R, Rosell-Polo JR, Morros JR, Ruiz-Hidalgo J, Vilaplana V, et al. Fuji-SfM dataset: A collection of annotated images and point clouds for Fuji apple detection and location using structure-from-motion photogrammetry. Data in Brief. 2020;30:105591.

47. Abendroth LJ, Elmore RW, Matthew J. Boyer and Marlay SK. *Corn Growth and Development*. Report no. PMR 1009, 2011. Ames, Iowa: Iowa State University Extension.

48. Cuturi M. Sinkhorn Distances: Lightspeed Computation of Optimal Transportation Distances. Advances in Neural Information Processing Systems. 2013;26:2292-300.

49. Richard S. Diagonal equivalence to matrices with prescribed row and column sums. The American Mathematical Monthly. 1967;74 4:402-5.

50. Boykov Y and Kolmogorov V. An experimental comparison of min-cut/max-flow algorithms for energy minimization in vision. IEEE Trans Pattern Anal Mach Intell. 2004;26 9:1124-37. doi:10.1109/tpami.2004.60.

51. Rusu RB and Cousins S. 3D is here: Point Cloud Library (PCL). In: *IEEE International Conference on Robotics and Automation* 2011, pp.1-4.

52. Charles RQ, Su H, Kaichun M and Guibas LJ. PointNet: Deep Learning on Point Sets for 3D Classification and Segmentation. In: *2017 IEEE Conference on Computer Vision and Pattern Recognition (CVPR)* 21-26 July 2017 2017, pp.77-85.

53. Shi WN, van de Zedde R, Jiang HY and Kootstra G. Plant-part segmentation using deep learning and multi-view vision. Biosys Eng. 2019;187:81-95. doi:10.1016/j.biosystemseng.2019.08.014.

54. Artzet S, Chen T-W, Chopard J, Brichet N, Mielewczik M, Cohen-Boulakia S, et al. Phenomenal: An automatic open source library for 3D shoot architecture reconstruction and analysis for image-based plant phenotyping. bioRxiv. 2019:805739. doi:10.1101/805739.

55. Liu Z, Tang H, Lin Y and Han S. Point-Voxel CNN for Efficient 3D Deep Learning. *33rd Conference on Neural Information Processing Systems*. Vancouver, Canada: Curran Associates, Inc., 2019, p. 965-75.
