# Supplementary material for: Label3DMaize: toolkit for 3D point cloud data annotation of maize shoots
Source: Gigascience. 2021 May 7;10(5):giab031. doi: 10.1093/gigascience/giab031 (PMC8105162; doi:10.1093/gigascience/giab031)
Supplement: giab031_Response_to_Reviewer_Comments_Original_Submission [file giab031_response_to_reviewer_comments_original_submission.pdf]

Author's Response To Reviewer Comments

Close

Response to Editor
Your manuscript "Label3DMaize: toolkit for 3D point cloud data annotation of maize shoots" (GIGA-D-20-00335) has been assessed by our reviewers. Based on these reports, and my own assessment as Editor, I am pleased to inform you that it is potentially acceptable for publication in GigaScience, once you have carried out some essential revisions suggested by our reviewers.
** We very much appreciate your careful reading of our manuscript and valuable suggestions. We have carefully considered the comments and have revised the manuscript accordingly.
Some descriptions of the steps involved are not clear and need better clarifications and details.
** We have simplified the seed point determination section (section 2.5.1), and supplemented pseudocode of optimal transportation matrix in section 2.5.2.
Comparison with at least 1-2 state of the art methods is also required. Another concern is the dependency of Label3DMaize on data generated through MVS-Pheno - which is commercial software - as we are an open science journal, work must be reproducible and all open and available for reuse without the need to access proprietary software. We'd need you to clarify this further and also provide tests to prove that it is not just dependent on MVS-Pheno.
** We conducted comparison of coarse segmentation with region growing and PointNet-based model (Section 3.4).
Label3DMaize does not depends on data generated through MVS-Pheno. Any point cloud data of maize shoot could be the input of this toolkit, such as data acquired using any 3D scanner, or point clouds reconstructed through manually captured multi-view images. Data used in the new supplemented section 3.4 were point cloud acquired using 3D scanner. Tomato point cloud data used in section 3.5 was downloaded from a literature. This have been clarified at the end of the first paragraph in section 4.2.
“Specifically, Label3DMaize does not depend on data generated through MVS-Pheno. Any point cloud of maize shoot can be the toolkit input, including data acquired using 3D scanners (Figure 7), or reconstructed from multi-view images acquired by handheld cameras.”
In addition, please register any new software application in the bio.tools and SciCrunch.org databases to receive RRID (Research Resource Identification Initiative ID) and biotoolsID identifiers, and include these in your manuscript. This will facilitate tracking, reproducibility and re-use of your tool.
** the biotoolsID is label3dmaize, which have been supplemented in the last paragraph in Introduction.
** We have uploaded all new data files and updated the latest version of Label3DMaize in GitHub.

Response to Reviewer #1:
This very interesting Research Article describes Label3DMaize, which is a toolkit that utilises a top-to-down segmentation algorithm to deliver fine point cloud segmentation of maize shoots. Multi-view images of maize cultivars were captured using the MVS-Pheno platform, which has been described previously (Wu et al., 2020, doi:10.34133/2020/1848437). The top-to-down coarse segmentation ensures topological accuracy. Additional morphological operations then improve the segmentation of maize plants, with the resultant 3D points being structured on connectivity and morphological features. A detailed workflow is described in the article.
Label3DMaize is publicly available on GitHub and has been ascribed an OSI-approved GPL-3.0 License. The software is easy to run, simply requiring MATLAB Runtime 9.2. Label3DMaize is straightforward and easy-to-use, and the interface enables users to rotate, zoom, translate, select points, and improve the segmentation. A pop-up window allows users to select points and to generate segmentations. Test data of individual maize plants are available from the GitHub archive.
This is a very easy-to-use toolkit and I recommend this paper for publication in GigaScience.
** We very much appreciate your careful reading of our manuscript and comments.

Response to Reviewer #2:
The authors give a practical tool for maize organ segmentation by an interactive way. The ideas and methods presented in this paper are straightforward and make sense for me. The Figs. and language are also sufficiently good and the obtained segmented results of the maize shoots show a clear, practical applicability as well. I recommend the paper to be accepted after minor revision. Below are some detail suggestion:
** We very much appreciate your careful reading of our manuscript and valuable suggestions. We have carefully considered the comments and have revised the manuscript accordingly.
1) The authors need to provide the contributions of the work at the end of the introduction to enhance the innovations of the paper.
** A brief contribution of the work have been provided at the end of the introduction. "The toolkit integrates clustering approaches and computer interactions supported through maize structural knowledge. Optimal transportation based coarse segmentation is satisfactory for basic segmentation tasks, and fine segmentation offers users way to calibrate the segmentation details. This plant-oriented tool could be used to segment point cloud data of various maize growth periods, and provide practical data labeling tool for segmentation research based on deep learning."
2) In the workflow of the segmentation, the initial segmentation is the key step, however, the descriptions of this part (pages 9 and 10) are not clear. I suggest the authors rephrase this part. It is enough to introduce the general ideas of the algorithm based on the transportation distances. The details of the algorithm can be put into the pseudocode.
** Section 2.5.1 has been rephrased and the pseudocode has been supplemented in section 2.5.2.
3) In some cases, the multiple maize shoots are hardly to separate. Does the Label3DMaize have the capability to segment multiple instances simultaneously? I mean whether the tool support multiple maize shoots as inputs? If you confront this situation, how do you process?
** Label3DMaize could not segment point cloud containing multiple instances. For this situation, the point cloud have to be preprocessed into separated individual shoots. Corresponding descriptions have been supplemented at the beginning of the last paragraph in section 4.2. "Label3DMaize is designed for individual shoots and does not support segmentation of multiple maize shoots. Thus, point clouds containing multiple shoots have to be preprocessed into individual shoot point clouds first, through spatial connection property of points, or interactively separated using commercial software (such as CloudCompare, Geomagic Studio, etc.). This shoot separation preprocess is easy for scenarios without cross organs. Thus, point cloud data acquisition is important for subsequent segmentation."
4) The authors mentioned that point clouds scanned by 3d scanners have much random noise than MVS point cloud. To the best of our knowledge, I think the maize shoot point clouds derived by the TLS or other similar scanners should have higher accuracy, precision, and less noise than MVS point clouds. The related sentences need to be reconsidered.
** Indeed, 3D scanners have higher accuracy and precision. In our practice, point clouds obtained using 3D scanner have many random noise for maize shoots, especially near the boundary of leaves (We use FARO X130 scanner). Point cloud reconstructed using mutli-view images through commercial software (such as PhotoScan) have less noise. However, I think this depends the 3D scanner or MVS reconstruction software. We modified the related description from "For shoots with much random noise obtained by 3D scanners, point cloud denoising should be performed first" into "For shoots with much random noise, point cloud denoising should be performed first".
5) In the experiments, the authors should conduct comparisons with at least one or two state-of-the-art methods.
** A new section 3.4 was supplemented in our manuscript. We conducted comparison of coarse segmentation with region growing and PointNet-based model.

Response to Reviewer #3:
Point cloud is the most important representation for shape information acquisition, and that is a useful form of shape information processing. Maize is one of the most important crops. Each maize has its own special shape, but it follow a strong rule of structures so that learning based tool could be developed and be useful.
High-precision segmentation tool is an important basis for mathematical modeling of crop growth, for crop measurement and for crop yield estimation. Deep learning method becomes effective for the classification and segmentation of point clouds, but it is difficult to deal directly with the problem of maize object segmentation. Therefore, the topic of the paper has important research value and technical challenges.
Based on point cloud shape analysis, a point cloud interactive segmentation method and a point cloud

interactive segmentation labeling tool are developed. The tool Label3DMaize is based on the basic knowledge of maize morphology, and that is effective for 3D point cloud data annotation/labeling of maize shoots. Experimental results show that segmentations of maize shoot are effective.
The contribution of this paper is in the use of the optimal transmission and point cloud clustering methods. Human computer interaction is included with maize morphological structure knowledge supported. This developed plant-oriented point cloud segmentation annotation/labeling tool could be used to point data of different reproductive period of the maize, and could be used to deep learning-based point cloud segmentation annotation of other crops also.
** We very much appreciate your careful reading of our manuscript and valuable suggestions. We have carefully considered the comments and have revised the manuscript accordingly.
Disadvantages and modification recommendations:
1. Since MRF method is used for precise segmentation of point cloud, complex interactive operations are needed. Segmentation results of the approach are not intuitive, it is recommended to introduce a more direct way to assist the MRF segmentation.
** This is a very practical suggestion for us to improve this toolkit. We added a new function to assist the MRF segmentation and the description was supplemented at the end of section 2.6. "In addition, users cloud assign organ label to the region of interest points after the above mentioned step 2, which offers a more direct way for fine segmentation.". As a result, this improvement directly reduced the segmentation time, see Table 3.
2. The annotation tool described in the article is mainly designed for the maize, authors should mention whether or not it could be properly extended to other specific crops. It is suggested to specify this extendibility of segmentation and related interactions, hoping to provide better reference for other crop researchers.
** We supplemented section 3.5 to demonstrate the extendibility of the toolkit on other plants, including tomato, cucumber, and wheat shoot.
3. It is suggested to further strengthen the use of maize morphology knowledge, and to promote the use of knowledge and data joint driven for segmentation and annotation.
** Thanks for this suggestion. The current version have integrated the structural knowledge of maize shoot for segmentation. In our further study, we will integrate the morphology knowledge through deep learning models.
4. It is suggested to further consider using the existing three-dimensional shape model of maize to guide the annotation/ labeling of new maize data.
** Thanks for this suggestion. Corresponding modifications have been made in section 4.3. "What's more, well segmented maize organ data could be used to build a 3D shape model of maize. All the above technologies or data will conversely simplify the segmentation and labeling processes of the toolkit."
5. Typos should be carefully checked
1) Line 55: Therefor === > Therefore
2) Line 127: update === > updates
3) Line 197: distance === > distances
4) Line 361: indicates === > indicate
5) Labelling (Line 383) or labeling (Line 68 and 409)? These should be consistent.
** Thanks for your careful reading and corresponding modifications have been made in the manuscript.
6. More very new papers on point cloud segmentation in the field computer vision and computer graphics should be cited, just like the following:
[1] F. Engelmann, M. Bokeloh, A. Fathi, B. Leibe and M. Niessner, "3D-MPA: Multi-Proposal Aggregation for 3D Semantic Instance Segmentation," 2020 IEEE/CVF Conference on Computer Vision and Pattern Recognition (CVPR), Seattle, WA, USA, pp. 9028-9037, 2020.
[2] Tao Ku, Remco C. Veltkamp, etc, SHREC 2020: 3D point cloud semantic segmentation for street scenes, Computers & Graphics, Volume 93, Pages 13-24, 2020.
** We have cited five related papers on point cloud segmentation in the field computer vision and computer graphics:
39. Engelmann F, Bokeloh M, Fathi A, Leibe B and Nießner M. 3D-MPA: Multi-Proposal Aggregation for 3D Semantic Instance Segmentation. In: 2020 IEEE/CVF Conference on Computer Vision and Pattern Recognition (CVPR) 13-19 June 2020 2020, pp.9028-37.
41. Chang AX, Funkhouser T, Guibas L, Hanrahan P, Huang Q, Li Z, et al. ShapeNet: An Information-Rich 3D Model Repository. Computer ence. 2015.
42. Hackel T, Savinov N, Ladicky L, Wegner JD, Schindler K and Pollefeys M. Semantic3D.net: A new Large-scale Point Cloud Classification Benchmark. 2017.
43. Behley J, Garbade M, Milioto A, Quenzel J, Behnke S, Stachniss C, et al. SemanticKITTI: A Dataset for Semantic Scene Understanding of LiDAR Sequences. In: 2019 IEEE/CVF International Conference on Computer Vision (ICCV) 27 Oct.-2 Nov. 2019 2019, pp.9296-306.

44. Ku T, Veltkamp RC, Boom B, Duque-Arias D, Velasco-Forero S, Deschaud JE, et al. SHREC 2020: 3D point cloud semantic segmentation for street scenes. Comput Graph-UK. 2020;93:13-24. doi:10.1016/j.cag.2020.09.006.

Response to Reviewer #4:
The authors describe a software called Label3DMaize to segment and annotate point cloud data. The software offers different types of segmentation methods, some of them faster than others. In terms of processing speed, Label3DMaize is faster than other platforms. The subject is of clear importance since highly precise phenotypes can be computed from 3D representations. Moreover, 3D representation allows the measurement of hard-to-measure or complex phenotypes.
** We very much appreciate your careful reading of our manuscript and valuable suggestions. We have carefully considered the comments and have revised the manuscript accordingly.
Some comments:
1) This software runs in Matlab, which is vaguely mentioned in the Additional files section. I consider it is important to mention this in the main text because this will impact the accessibility of the software for many potential users.
** Indeed, this should be mentioned in the main text. We supplemented this at the beginning of section 3.1: “The Label3DMaize toolkit was developed using Matlab”.
2) The 3D point cloud data used in this study was generated by a system (MVS-Pheno) that the same authors developed just recently (the paper was published early 2020). MVS-Pheno is a commercial software it seems. My main concern is the dependency of Label3DMaize on data generated through MVS-Pheno. Is this going to work on data generated on a different system? If so, additional tests showing that must be included in the present study.
** MVS-Pheno facilitates high-throughput data acquisition and data management of individual plants. However, Label3DMaize does not depends on this platform. Any point cloud of maize shoot can be the input of the toolkit, including data acquired using 3D scanners, or reconstructed point cloud from multi-view images acquired by handheld cameras. To demonstrate this, data used for comparison with other methods in section 3.4 were acquired using a 3D scanner. And we supplemented this explanation at the end of the first paragraph in section 4.2: “Specifically, Label3DMaize does not depend on data generated through MVS-Pheno. Any point cloud of maize shoot can be the toolkit input, including data acquired using 3D scanners (Figure 7), or reconstructed from multi-view images acquired by handheld cameras.”
3) Both the introduction and the discussion sections are a little heavy; there is a constant reuse of ideas, which becomes repetitive and tedious to read. I suggest reducing the text considerably.
** Indeed, there is a constant reuse of ideas. We simplified some of the contents. See the beginning of section 4.1.
4) Lines 48-49. The authors say that multiple MVS platforms have been developed, but they just mention one reference (reference 16).
** Three references about other MVS platforms have been supplemented:
19. Nguyen CV, Fripp J, Lovell DR, Furbank R, Kuffner P, Daily H, et al. 3D Scanning System for Automatic High-Resolution Plant Phenotyping. 2016 International Conference on Digital Image Computing: Techniques and Applications (DICTA). 2016, p. 1-8.
20. Cao W, Zhou J, Yuan Y, Ye H, Nguyen HT, Chen J, et al. Quantifying Variation in Soybean Due to Flood Using a Low-Cost 3D Imaging System. Sensors. 2019;19 12:2682. doi:10.3390/s19122682.
21. Bernotas G, Scorza LCT, Hansen MF, Hales IJ, Halliday KJ, Smith LN, et al. A photometric stereo-based 3D imaging system using computer vision and deep learning for tracking plant growth. GigaScience. 2019;8 5:15. doi:10.1093/gigascience/giz056.
5) Examples of previous work regarding 3D point cloud data are presented in the Introduction section, which provides the reader a good context. However, it seems that the examples are presented one after another without a proper link.
** These data acquisition technologies are well-known for researchers in this area. Simple listed these key technologies and citing representative references is enough. Detailed explanation about their proper link will make the introduction more heavy. We supplement a review article that describe the link of these technologies.
10. Jin SC, Sun XL, Wu FF, Su YJ, Li YM, Song SL, et al. Lidar sheds new light on plant phenomics for plant breeding and management: Recent advances and future prospects. ISPRS-J Photogramm Remote Sens. 2021;171:202-23. doi:10.1016/j.isprsjprs.2020.11.006.
6) Clearly, this is a relevant tool that could benefit researchers studying plant morphology as well as breeders. However, I don't think this study fits well within the scope and requirements of GigaScience (e.g. lack of novelty, more tests are required, why not using this in a different species with similar plant

structure?, high dependency in other tools, etc…).
** We have supplemented application on tomato, cucumber, and wheat. And we have clarified the toolkit did not depends on other tools.
7) Section 4.3 could be integrated within other Discussion subsections.
** This subsection (section 4.3) describes the work we might conduct in the future, which is different from the other parts of the discussion. Thus, in our opinion, this is clearer than merging into other subsections.

Close
