# Supplementary material for: Label3DMaize: toolkit for 3D point cloud data annotation of maize shoots
Source: Gigascience. 2021 May 7;10(5):giab031. doi: 10.1093/gigascience/giab031 (PMC8105162; doi:10.1093/gigascience/giab031)
Supplement: giab031_Response_to_Reviewer_Comments_Revision_1 [file giab031_response_to_reviewer_comments_revision_1.pdf]

Author's Response To Reviewer Comments

Close

** Dear editor, we are pleased to receive this letter and have revised the comments accordingly. The revised contents are in red fonts in the manuscript. Detailed responses are as follows:
AU: Please add Availability of supporting data here. Where is the training dataset associated with this work? We need all supporting data openly available – this can be hosted in GigaDB if not openly hosted elsewhere.
The final sentence should say the following "Other supporting data are openly available in the GigaScience repository, GigaDB [xx]".
** Availability of supporting data, supporting source code and requirements have been supplemented.
** The training dataset was used in PointNet-based segmentation for comparison, which was supplemented in R1 version as requested by reviewer 2. Producing the dataset was labor-intensive and time-consuming. The dataset is the competitive content in one of our subsequent paper. Thus, we are afraid that if the dataset is openly available here, the subsequent paper might be affected for publication. What's more, the supporting data and code in this manuscript are openly available, and this dataset is not the core data of this manuscript. So, please consider if it is possible that we do not open this dataset.
AU: Please list all abbreviations used in the main text here in alphabetical order.
** Corresponding modifications have been made in the manuscript.
AU: All financial and non-financial competing interests must be declared in this section. See our editorial policies for a full explanation of competing interests. Where an author gives no competing interests, the listing will read 'The author(s) declare that they have no competing interests'. If you are unsure whether you or any of your co-authors have a competing interest please contact the editorial office.
** Corresponding modifications have been made in the manuscript.
AU: All sources of funding for the research reported should be declared. The role of the funding body in the design of the study and collection, analysis, and interpretation of data and in writing the manuscript should be declared. Please use FundRef to report funding sources and include the award/grant number, and the name of the Principal Investigator of the grant.
** Corresponding modifications have been made in the manuscript.
AU: The individual contributions of authors to the manuscript should be specified in this section. Guidance and criteria for authorship can be found in our editorial policies. We would recommend you follow some kind of standardised taxonomy like the CASRAI CRediT (Contributor Roles Taxonomy).
** Corresponding modifications have been made in the manuscript.
AU: The highlighted sentences here should be moved to the "Funding" section and FunRef format should be followed. See my comment above.
** Corresponding modifications have been made in the manuscript.
AU: please add a place holder reference for the GigaDB DOI reference. This will be finalized when your DOI is ready.
** We have added this at the end of the references:
56. Miao T, Wen WL, Wu S, et al. Supporting data for "Label3DMaize: toolkit for 3D point cloud data annotation of maize shoots." GigaScience Database 2021. http://dx.doi.org/....

Close
