# Supplementary material for: Label3DMaize: toolkit for 3D point cloud data annotation of maize shoots
Source: Gigascience. 2021 May 7;10(5):giab031. doi: 10.1093/gigascience/giab031 (PMC8105162; doi:10.1093/gigascience/giab031)
Supplement: giab031_Reviewer_1_Report_Original_Submission — Chris Armit -- 12/18/2020 Reviewed [file giab031_reviewer_1_report_original_submission.pdf]

Reviewer Report

Title: Label3DMaize: toolkit for 3D point cloud data annotation of maize shoots

Version: Original Submission **Date:** 12/18/2020

Reviewer name: Chris Armit

Reviewer Comments to Author:

This very interesting Research Article describes Label3DMaize, which is a toolkit that utilises a top-to-down segmentation algorithm to deliver fine point cloud segmentation of maize shoots. Multi-view images of maize cultivars were captured using the MVS-Pheno platform, which has been described previously (Wu et al., 2020, doi:10.34133/2020/1848437). The top-to-down coarse segmentation ensures topological accuracy. Additional morphological operations then improve the segmentation of maize plants, with the resultant 3D points being structured on connectivity and morphological features. A detailed workflow is described in the article.
Label3DMaize is publicly available on GitHub and has been ascribed an OSI-approved GPL-3.0 License. The software is easy to run, simply requiring MATLAB Runtime 9.2. Label3DMaize is straightforward and easy-to-use, and the interface enables users to rotate, zoom, translate, select points, and improve the segmentation. A pop-up window allows users to select points and to generate segmentations. Test data of individual maize plants are available from the GitHub archive.
This is a very easy-to-use toolkit and I recommend this paper for publication in GigaScience.

Methods

Are the methods appropriate to the aims of the study, are they well described, and are necessary controls included? Choose an item.

Conclusions

Are the conclusions adequately supported by the data shown? Choose an item.

Reporting Standards

Does the manuscript adhere to the journal’s guidelines on [minimum standards of reporting?] Choose an item.

Choose an item.

Statistics

Are you able to assess all statistics in the manuscript, including the appropriateness of statistical tests used? Choose an item.

Quality of Written English

Please indicate the quality of language in the manuscript: Choose an item.

Declaration of Competing Interests

Please complete a declaration of competing interests, considering the following questions:

- Have you in the past five years received reimbursements, fees, funding, or salary from an organisation that may in any way gain or lose financially from the publication of this manuscript, either now or in the future?
- Do you hold any stocks or shares in an organisation that may in any way gain or lose financially from the publication of this manuscript, either now or in the future?
- Do you hold or are you currently applying for any patents relating to the content of the manuscript?
- Have you received reimbursements, fees, funding, or salary from an organization that holds or has applied for patents relating to the content of the manuscript?
- Do you have any other financial competing interests?
- Do you have any non-financial competing interests in relation to this paper?

If you can answer no to all of the above, write 'I declare that I have no competing interests' below. If your reply is yes to any, please give details below.

I declare that I have no competing interests.

I agree to the open peer review policy of the journal. I understand that my name will be included on my report to the authors and, if the manuscript is accepted for publication, my named report including any attachments I upload will be posted on the website along with the authors' responses. I agree for my report to be made available under an Open Access Creative Commons CC-BY license (http://creativecommons.org/licenses/by/4.0/). I understand that any comments which I do not wish to be included in my named report can be included as confidential comments to the editors, which will not be published.

Choose an item.

To further support our reviewers, we have joined with Publons, where you can gain additional credit to further highlight your hard work (see: https://publons.com/journal/530/gigascience). On publication of this paper, your review will be automatically added to Publons, you can then choose whether or not to claim your Publons credit. I understand this statement.

Yes Choose an item.
