# Supplementary material for: Label3DMaize: toolkit for 3D point cloud data annotation of maize shoots
Source: Gigascience. 2021 May 7;10(5):giab031. doi: 10.1093/gigascience/giab031 (PMC8105162; doi:10.1093/gigascience/giab031)
Supplement: giab031_Reviewer_2_Report_Original_Submission — Dong Chen -- 12/30/2020 Reviewed [file giab031_reviewer_2_report_original_submission.pdf]

Reviewer Report

Title: Label3DMaize: toolkit for 3D point cloud data annotation of maize shoots

Version: Original Submission **Date:** 12/30/2020

Reviewer name: Dong Chen

Reviewer Comments to Author:

The authors give a practical tool for maize organ segmentation by an interactive way. The ideas and methods presented in this paper are straightforward and make sense for me. The Figs. and language are also sufficiently good and the obtained segmented results of the maize shoots show a clear, practical applicability as well. I recommend the paper to be accepted after minor revision. Below are some detail suggestion:.

1) The authors need to provide the contributions of the work at the end of the introduction to enhance the innovations of the paper.
2) In the workflow of the segmentation, the initial segmentation is the key step, however, the descriptions of this part (pages 9 and 10) are not clear. I suggest the authors rephrase this part. It is enough to introduce the general ideas of the algorithm based on the transportation distances. The details of the algorithm can be put into the pseudocode.
3) In some cases, the multiple maize shoots are hardly to separate. Does the Label3DMaize have the capability to segment multiple instances simultaneously? I mean whether the tool support multiple maize shoots as inputs? If you confront this situation, how do you process?
4) The authors mentioned that point clouds scanned by 3d scanners have much random noise than MVS point cloud. To the best of our knowledge, I think the maize shoot point clouds derived by the TLS or other similar scanners should have higher accuracy, precision, and less noise than MVS point clouds. The related sentences need to be reconsidered.
5) In the experiments, the authors should conduct comparisons with at least one or two state-of-the-art methods.

Methods

Are the methods appropriate to the aims of the study, are they well described, and are necessary controls included? Choose an item.

Conclusions

Are the conclusions adequately supported by the data shown? Choose an item.

Reporting Standards

Does the manuscript adhere to the journal's guidelines on [minimum standards of reporting?]() Choose an item.

Choose an item.

Statistics

Are you able to assess all statistics in the manuscript, including the appropriateness of statistical tests used? Choose an item.

Quality of Written English

Please indicate the quality of language in the manuscript: Choose an item.

Declaration of Competing Interests

Please complete a declaration of competing interests, considering the following questions:

- Have you in the past five years received reimbursements, fees, funding, or salary from an organisation that may in any way gain or lose financially from the publication of this manuscript, either now or in the future?
- Do you hold any stocks or shares in an organisation that may in any way gain or lose financially from the publication of this manuscript, either now or in the future?
- Do you hold or are you currently applying for any patents relating to the content of the manuscript?
- Have you received reimbursements, fees, funding, or salary from an organization that holds or has applied for patents relating to the content of the manuscript?
- Do you have any other financial competing interests?
- Do you have any non-financial competing interests in relation to this paper?

If you can answer no to all of the above, write 'I declare that I have no competing interests' below. If your reply is yes to any, please give details below.

I declare that I have no competing interests.

I agree to the open peer review policy of the journal. I understand that my name will be included on my report to the authors and, if the manuscript is accepted for publication, my named report including any attachments I upload will be posted on the website along with the authors' responses. I agree for my report to be made available under an Open Access Creative Commons CC-BY license (http://creativecommons.org/licenses/by/4.0/). I understand that any comments which I do not wish to be included in my named report can be included as confidential comments to the editors, which will not be published.

Choose an item.

To further support our reviewers, we have joined with Publons, where you can gain additional credit to further highlight your hard work (see: https://publons.com/journal/530/gigascience). On publication of this paper, your review will be automatically added to Publons, you can then choose whether or not to claim your Publons credit. I understand this statement.

Yes Choose an item.
