# Supplementary material for: Label3DMaize: toolkit for 3D point cloud data annotation of maize shoots
Source: Gigascience. 2021 May 7;10(5):giab031. doi: 10.1093/gigascience/giab031 (PMC8105162; doi:10.1093/gigascience/giab031)
Supplement: giab031_Reviewer_3_Report_Original_Submission — Xiaopeng Zhang -- 12/31/2020 Reviewed [file giab031_reviewer_3_report_original_submission.pdf]

Reviewer Report

Title: Label3DMaize: toolkit for 3D point cloud data annotation of maize shoots

Version: Original Submission **Date:** 12/31/2020

Reviewer name: Xiaopeng Zhang

Reviewer Comments to Author:

Point cloud is the most important representation for shape information acquisition, and that is a useful form of shape information processing. Maize is one of the most important crops. Each maize has its own special shape, but it follow a strong rule of structures so that learning based tool could be developed and be useful.
High-precision segmentation tool is an important basis for mathematical modeling of crop growth, for crop measurement and for crop yield estimation. Deep learning method becomes effective for the classification and segmentation of point clouds, but it is difficult to deal directly with the problem of maize object segmentation. Therefore, the topic of the paper has important research value and technical challenges.
Based on point cloud shape analysis, a point cloud interactive segmentation method and a point cloud interactive segmentation labeling tool are developed. The tool Label3DMaize is based on the basic knowledge of maize morphology, and that is effective for 3D point cloud data annotation/labeling of maize shoots. Experimental results show that segmentations of maize shoot are effective.
The contribution of this paper is in the use of the optimal transmission and point cloud clustering methods. Human computer interaction is included with maize morphological structure knowledge supported. This developed plant-oriented point cloud segmentation annotation/labeling tool could be used to point data of different reproductive period of the maize, and could be used to deep learning-based point cloud segmentation annotation of other crops also.

Disadvantages and modification recommendations:
1. Since MRF method is used for precise segmentation of point cloud, complex interactive operations are needed. Segmentation results of the approach are not intuitive, it is recommended to introduce a more direct way to assist the MRF segmentation.
2. The annotation tool described in the article is mainly designed for the maize, authors should mention whether or not it could be properly extended to other specific crops. It is suggested to specify this extendibility of segmentation and related interactions, hoping to provide better reference for other crop researchers.
3. It is suggested to further strengthen the use of maize morphology knowledge, and to promote the use of knowledge and data joint driven for segmentation and annotation.
4. It is suggested to further consider using the existing three-dimensional shape model of maize to guide the annotation/ labeling of new maize data.
5. Typos should be carefully checked

1) Line 55: Therefor === > Therefore
2) Line 127: update === > updates
3) Line 197: distance === > distances
4) Line 361: indicates === > indicate
5) Labelling (Line 383) or labeling (Line 68 and 409)? These should be consistent.
6. More very new papers on point cloud segmentation in the field computer vision and computer graphics should be cited, just like the following:
[1] F. Engelmann, M. Bokeloh, A. Fathi, B. Leibe and M. Niessner, "3D-MPA: Multi-Proposal Aggregation for 3D Semantic Instance Segmentation," 2020 IEEE/CVF Conference on Computer Vision and Pattern Recognition (CVPR), Seattle, WA, USA, pp. 9028-9037, 2020.
[2] Tao Ku, Remco C. Veltkamp, etc, SHREC 2020: 3D point cloud semantic segmentation for street scenes, Computers & Graphics, Volume 93, Pages 13-24, 2020.

Methods

Are the methods appropriate to the aims of the study, are they well described, and are necessary controls included? Choose an item.

Conclusions

Are the conclusions adequately supported by the data shown? Choose an item.

Reporting Standards

Does the manuscript adhere to the journal's guidelines on [minimum standards of reporting?] Choose an item.

Choose an item.

Statistics

Are you able to assess all statistics in the manuscript, including the appropriateness of statistical tests used? Choose an item.

Quality of Written English

Please indicate the quality of language in the manuscript: Choose an item.

Declaration of Competing Interests

Please complete a declaration of competing interests, considering the following questions:

- Have you in the past five years received reimbursements, fees, funding, or salary from an organisation that may in any way gain or lose financially from the publication of this manuscript, either now or in the future?
- Do you hold any stocks or shares in an organisation that may in any way gain or lose financially from the publication of this manuscript, either now or in the future?
- Do you hold or are you currently applying for any patents relating to the content of the manuscript?
- Have you received reimbursements, fees, funding, or salary from an organization that holds or has applied for patents relating to the content of the manuscript?
- Do you have any other financial competing interests?
- Do you have any non-financial competing interests in relation to this paper?

If you can answer no to all of the above, write 'I declare that I have no competing interests' below. If your reply is yes to any, please give details below.

I declare that I have no competing interests.

I agree to the open peer review policy of the journal. I understand that my name will be included on my report to the authors and, if the manuscript is accepted for publication, my named report including any attachments I upload will be posted on the website along with the authors' responses. I agree for my report to be made available under an Open Access Creative Commons CC-BY license (http://creativecommons.org/licenses/by/4.0/). I understand that any comments which I do not wish to be included in my named report can be included as confidential comments to the editors, which will not be published.

Choose an item.

To further support our reviewers, we have joined with Publons, where you can gain additional credit to further highlight your hard work (see: https://publons.com/journal/530/gigascience). On publication of this paper, your review will be automatically added to Publons, you can then choose whether or not to claim your Publons credit. I understand this statement.

Yes Choose an item.
