# Supplementary material for: Label3DMaize: toolkit for 3D point cloud data annotation of maize shoots
Source: Gigascience. 2021 May 7;10(5):giab031. doi: 10.1093/gigascience/giab031 (PMC8105162; doi:10.1093/gigascience/giab031)
Supplement: giab031_Reviewer_4_Report_Original_Submission — Luis Diaz-Garcia -- 1/4/2021 Reviewed [file giab031_reviewer_4_report_original_submission.pdf]

Reviewer Report

Title: Label3DMaize: toolkit for 3D point cloud data annotation of maize shoots

Version: Original Submission **Date:** 1/4/2021

Reviewer name: Luis Diaz-Garcia

Reviewer Comments to Author:

The authors describe a software called Label3DMaize to segment and annotate point cloud data. The software offers different types of segmentation methods, some of them faster than others. In terms of processing speed, Label3DMaize is faster than other platforms. The subject is of clear importance since highly precise phenotypes can be computed from 3D representations. Moreover, 3D representation allows the measurement of hard-to-measure or complex phenotypes.

Some comments:

1) This software runs in Matlab, which is vaguely mentioned in the Additional files section. I consider it is important to mention this in the main text because this will impact the accessibility of the software for many potential users.

2) The 3D point cloud data used in this study was generated by a system (MVS-Pheno) that the same authors developed just recently (the paper was published early 2020). MVS-Pheno is a commercial software it seems. My main concern is the dependency of Label3DMaize on data generated through MVS-Pheno. Is this going to work on data generated on a different system? If so, additional tests showing that must be included in the present study.

3) Both the introduction and the discussion sections are a little heavy; there is a constant reuse of ideas, which becomes repetitive and tedious to read. I suggest reducing the text considerably.

4) Lines 48-49. The authors say that multiple MVS platforms have been developed, but they just mention one reference (reference 16).

5) Examples of previous work regarding 3D point cloud data are presented in the Introduction section, which provides the reader a good context. However, it seems that the examples are presented one after another without a proper link.

6) Clearly, this is a relevant tool that could benefit researchers studying plant morphology as well as breeders. However, I don't think this study fits well within the scope and requirements of GigaScience (e.g. lack of novelty, more tests are required, why not using this in a different species with similar plant structure?, high dependency in other tools, etc…).

7) Section 4.3 could be integrated within other Discussion subsections.

That said, I would consider that the work is not suitable for publication in its present form.

Methods

Are the methods appropriate to the aims of the study, are they well described, and are necessary controls included? Choose an item.

Conclusions

Are the conclusions adequately supported by the data shown? Choose an item.

Reporting Standards

Does the manuscript adhere to the journal's guidelines on [minimum standards of reporting?] Choose an item.

Choose an item.

Statistics

Are you able to assess all statistics in the manuscript, including the appropriateness of statistical tests used? Choose an item.

Quality of Written English

Please indicate the quality of language in the manuscript: Choose an item.

Declaration of Competing Interests

Please complete a declaration of competing interests, considering the following questions:

- Have you in the past five years received reimbursements, fees, funding, or salary from an organisation that may in any way gain or lose financially from the publication of this manuscript, either now or in the future?
- Do you hold any stocks or shares in an organisation that may in any way gain or lose financially from the publication of this manuscript, either now or in the future?
- Do you hold or are you currently applying for any patents relating to the content of the manuscript?
- Have you received reimbursements, fees, funding, or salary from an organization that holds or has applied for patents relating to the content of the manuscript?
- Do you have any other financial competing interests?
- Do you have any non-financial competing interests in relation to this paper?

If you can answer no to all of the above, write 'I declare that I have no competing interests' below. If your reply is yes to any, please give details below.

I declare that I have no competing interests

I agree to the open peer review policy of the journal. I understand that my name will be included on my report to the authors and, if the manuscript is accepted for publication, my named report including any attachments I upload will be posted on the website along with the authors' responses. I agree for my report to be made available under an Open Access Creative Commons CC-BY license (http://creativecommons.org/licenses/by/4.0/). I understand that any comments which I do not wish to

be included in my named report can be included as confidential comments to the editors, which will not be published.

Choose an item.

To further support our reviewers, we have joined with Publons, where you can gain additional credit to further highlight your hard work (see: https://publons.com/journal/530/gigascience). On publication of this paper, your review will be automatically added to Publons, you can then choose whether or not to claim your Publons credit. I understand this statement.

Yes Choose an item.
